# Peptidomics Study of Plant-Based Meat Analogs as a Source of Bioactive Peptides

**DOI:** 10.3390/foods12051061

**Published:** 2023-03-02

**Authors:** Shuguang Wang, Mouming Zhao, Hongbing Fan, Jianping Wu

**Affiliations:** 1 Department of Agricultural, Food and Nutritional Science, University of Alberta, Edmonton, AB T6G 2P5, Canada; 2 School of Food Science and Engineering, South China University of Technology, Guangzhou 510640, China

**Keywords:** plant-based meat analogs, protein hydrolysates, nutritional property, peptide profile, bioactive assessment

## Abstract

The demand for plant-based meat analogs (PBMA) is on the rise as a strategy to sustain the food protein supply while mitigating environmental change. In addition to supplying essential amino acids and energy, food proteins are known sources of bioactive peptides. Whether protein in PBMA affords similar peptide profiles and bioactivities as real meat remains largely unknown. The purpose of this study was to investigate the gastrointestinal digestion fate of beef and PBMA proteins with a special focus on their potential as precursors of bioactive peptides. Results showed that PBMA protein showed inferior digestibility than that in beef. However, PBMA hydrolysates possessed a comparable amino acid profile to that of beef. A total of 37, 2420 and 2021 peptides were identified in the gastrointestinal digests of beef, Beyond Meat and Impossible Meat, respectively. The astonishingly fewer peptides identified from beef digest is probably due to the near-full digestion of beef proteins. Almost all peptides in Impossible Meat digest were from soy, whereas 81%, 14% and 5% of peptides in Beyond Meat digest were derived from pea, rice and mung proteins, respectively. Peptides in PBMA digests were predicted to exert a wide range of regulatory roles and were shown to have ACE inhibitory, antioxidant and anti-inflammatory activities, supporting the potential of PBMA as a source of bioactive peptides.

## 1. Introduction

A growing global population poses critical challenges in sustaining protein supply under already constrained resources and alarming concerns over climate change. Among various strategies towards sustainable protein production such as cellular agriculture (i.e., cultured meat), alternative proteins (i.e., terrestrial plant, insect and seaweed) and valorization of agricultural by-products [1,2], developing plant-based meat analogs (PBMA) is an attractive solution to replace traditional livestock production [3]. The market shares of alternative proteins remain low when compared with meat, even though governments and innovative companies increasingly advertise these alternatives to traditional meat products or dishes, such as plant-based burgers [4]. One major hurdle is consumer acceptance; in comparison, insects showed the lowest acceptance, followed by cultured meat, while terrestrial plant-based alternatives have the highest acceptance level [5]. The consumer acceptance of alternative proteins showed to be closely relevant to the drivers of taste and health, the color and aroma inherited, familiarity, food neophobia and disgust [1,2].

Since the successful launch of Beyond Meat and Impossible Meat, the market of PBMA has been on the rise; the global plant protein-based meat market is estimated to be approximately USD 21 billion by 2025 [6]. From a nutritional point of view, PBMA has unique advantages: its negligible cholesterol content, low fat content and high protein content with a well-balanced amino acids pattern [7,8]. McClements et al. reported that PBMA burgers contained fewer calories, cholesterol and fat than conventional beef burgers, despite nearly equal protein content [9]. However, there are continuous debates over the health implications of PMBA due to the addition of additives and the use of highly processed ingredients [3,8]. The health benefits of plant foods are likely compromised in PBMA. There is a need to develop clean labels and minimally processed products. For instance, the clean-labelled ProDiem™Refresh Soy is characterized by its sustainable and optimized nutrients to simulate/fulfill a protein intake similar to egg/milk [10].

A wide range of alternative proteins is explored for use in PBMA, especially those from grains and legumes, such as soy, pea, wheat, mung and lentil [11]. However, terrestrial plant proteins commonly possess inferior digestibility to that of livestock proteins, which challenges the nutritional profile of protein in meat analogs [12]. For example, Xie et al. reported that real meat (pork and beef) exhibited higher digestibility than that of PBMA during simulated gastrointestinal digestion, and the digestibility of PBMA depends on the origin and structure of proteins as well as the method of protein processing [13]. Food proteins are known as good sources of bioactive peptides. Bioactive peptides usually consist of 2–20 amino acids in length that are encrypted in their parent proteins and can exert regulatory roles once released in certain scenarios, including the gastrointestinal tract [1]. Given its increasing role in human dietary patterns, it is imperative to understand the potential of PBMA as the precursor of bioactive peptides. For instance, Chen et al. showed the formation of higher molecular weight and higher hydrophobicity in PBMA-derived peptides (soy and wheat proteins) than in chicken breast [14]. Xie et al. reported a larger number of peptides were identified from real meat than those of PBMA after simulated gastrointestinal digestion [13].

However, PBMA used in previous studies was prepared experimentally; research on commercial PBMA, especially from Beyond Meat and Impossible Meat, two major producers, are rarely reported. Simultaneously, systematic studies on the gastrointestinal fate, especially peptide profile and bioactivities after gastrointestinal digestion of PBMA, are still insufficiently understood. Meanwhile, there is no doubt that the peptide fragments released from real meat and PBMA are diverse due to their different parent protein sequences. Thus, the potential health benefits of these peptide fragments released from real meat and PBMA will also differ. Additionally, peptidomics and bioinformatics are emerging tools for identifying and predicting peptide profiling, bioavailability and bioactivity of bioactive peptides [15]. Hence, exploration of the digestibility and peptide profile after gastrointestinal digestion with the aid of peptidomics and bioinformatics will facilitate our understanding of the potential health benefits of PBMA.

The purpose of this study was to compare the in vitro gastrointestinal digestion fate of beef and PBMA (from Beyond Meat and Impossible Meat) with a special focus on their potential as precursors of bioactive peptides through assessing digestibility and peptide profiles and evaluate the relationship between peptide features and biofunctions (angiotensin-converting enzyme (ACE) inhibition, antioxidant and anti-inflammation).

## 2. Materials and Methods

### 2.1. Materials

Cooked patties of beef hamburger and Beyond Meat hamburger were bought from A&W (Edmonton, Alberta, Canada), and cooked patties of Impossible Meat burger were bought from Burger King (Edmonton, AB, Canada). ACE (from rabbit lung), hippuryl-His-Leu (HHL), pepsin (porcine gastric mucosa), pancreatin (porcine pancreas), 2,4,6-trinitrobenzenesulfonic acid (TNBS), cytochrome C, aprotinin, vitamin B12, (glycine)_3_, dithiothreitol (DTT) and angiotensin II (Ang II) were obtained from Sigma (Oakville, ON, Canada). Vascular smooth muscle A7r5 cell line was purchased from ATCC (Manassas, VA, USA). Dulbecco’s modified Eagle’s medium (DMEM), fetal bovine serum (FBS), 4-(2-68 hydroxyethyl)-1-piperazineethanesulfonic acid (HEPES) and non-essential amino acids (NEAA) were obtained from Gibco Invitrogen (Burlington, ON, Canada). Dihydroethidium (DHE) was purchased from Biotium (Fremont, CA, USA). Solvents used for UPLC were of chromatographic grade. Other chemicals applied were of analytical grade.

### 2.2. Preparation of Beef and PBMA Gastrointestinal Digests

The cooked beef patties and plant-based patties (Beyond Meat and Impossible Meat) in this study were bought from stores. Minced beef and PBMA were suspended in ddH_2_O and then exposed to two-step simulated gastrointestinal digestion [16]. Briefly, beef and PBMA (5% protein, *w*/*v*) were hydrolyzed by pepsin (1% protease/substrate, *w*/*w* protein) at pH 2.0 and 37 °C for 2.0 h, and then the digests were adjusted to pH 7.5 for another 2.0 h of hydrolysis with pancreatin (1% protease/substrate, *w/w* protein). Hydrolysis was terminated by heating the slurry at 95 °C for 10 min to inactive the proteases. Subsequently, the mixtures were centrifuged (8000× *g*, 15 min, 4 °C) to collect the supernatants, which were filtered by qualitative filter paper before being lyophilized to obtain the hydrolysates including BfP (cooked beef-pepsin), BfPP (cooked beef-pepsin-pancreatin), ByP (cooked Beyond Meat-pepsin), ByPP (cooked Beyond Meat-pepsin-pancreatin), ImP (cooked Impossible Meat-pepsin) and ImPP (cooked Impossible Meat-pepsin-pancreatin).

### 2.3. Molecular Weight Distribution

The molecular weight distribution of beef and PBMA hydrolysates were performed by sodium dodecyl sulfate-polyacrylamide gel electrophoresis (SDS-PAGE) and size exclusion chromatography according to the methods of Laemmli et al. [17] and Fan et al. [18], respectively. Briefly, for SDS-PAGE, beef and PBMA hydrolysates were initially dissolved in water at a concentration of 10 mg/mL and then diluted using a 2 × Laemmli sample buffer containing 5% *β*-mecaptoethanol at a volume ratio of 1:1. The prepared beef and PBMA hydrolysates were heated to 95 °C for 5 min before 20 µL of them were loaded to 16.5% Mini-Protean Tris-Tricine gel in a Mini-PROTEAN Tetra Cell with a PowerPac Basic electrophoresis apparatus (Bio-Rad, CA, USA) at a constant 150 V voltage. Gels were stained by Coomassie brilliant blue R250 dye and further destained by destaining buffer (ddH_2_O:methanol:acetic acid = 5:4:1, *v*/*v*/*v*), and then were scanned through an Alpha Innotech gel scanner (San Leandro, CA, USA). On the other hand, the molecular weight distribution was analyzed by size exclusion chromatography connecting with an AKTA explorer 10XT system (GE Healthcare, Uppsala, Sweden) with a Superdex peptide 10/300 GL column. Beef and PBMA hydrolysates were dissolved in 30% ACN containing 0.1% TFA. Subsequently, 100 µL beef and PBMA hydrolysates at a concentration of 1 mg/mL were injected into the Superdex peptide 10/300 GL column and eluted at an isocratic gradient with a flow rate of 0.5 mL/min. Peaks were monitored at 220 nm. The molecular weight was calibrated by a protein marker mixture in SDS-PAGE, whereas aprotinin, cytochrome C, (glycine)_3_ and vitamin B12 were used as molecular weight markers in size exclusion chromatography.

### 2.4. Degree of Hydrolysis (DH) and Amino Acid Compositions

The DH of beef and PBMA hydrolysates were evaluated using the TNBS method [19]. The amino acids analysis of beef and PBMA hydrolysates were determined according to the method of Zheng et al. [20].

### 2.5. Identification of Peptides by LC-MS/MS

The gastrointestinal-digested beef and PBMA hydrolysates were analyzed by liquid chromatography-tandem mass spectrometry (LC-MS/MS) on an Atlantis dC_18_ UPLC column (Waters, Milford, MA, USA) using a nano-Acquity RP-UPLC system, coupled with a Micromass Quadrupole Time-of-Flight (Q-TOF) premier mass spectrometer (Bruker, Bremen, Germany), as previously described [16]. Solvents were chromatographic grade acetonitrile (mobile phase B) and H_2_O (mobile phase A) containing 0.1% formic acid. The gradient program was set as 1%–60%–95% mobile phase B according to 0–2–40–55 min. Mass spectra were set in the positive-ion mode. The quadrupole ion energy was set at 4.0 eV, while the collision-inducing dissociation energy was set at 8–50 eV. The parameters for the ESI interface were as follows: 180 °C drying gas temperature, 8.0 L/min drying gas flow and 1.5 bar ESI nebulizer pressure. Data were interpreted by searching Mascot. The major parent protein sequences of beef, pea, soy, mungbean, rice and potato were obtained from the UniProtKB [21].

### 2.6. ACE Inhibition Assay

ACE inhibition was measured by referring to the method of Wu et al. [22]. ACE, HHL, beef and PBMA hydrolysates were dissolved and diluted with 100 mM potassium phosphate buffer containing 300 mM NaCl (pH 8.3). Substrate HHL (50 μL, 5 mM) and beef/PBMA hydrolysate (10 μL) were initially mixed and preincubated at 37 °C for 5 min in a 2 mL polypropylene centrifuge tube, and then 20 μL of preincubated ACE (37 °C, 2 mU) was added and reacted for another half an hour by an Eppendorf Thermomixer R (Brinkmann Instruments, NY, USA). The reaction was terminated by further adding 1 M HCl (125 μL) and then analyzed using an UPLC system combined with an Acquity BEH C18 column (1.7 μm, 2.1 mm × 50 mm). Solvents were chromatographic grade acetonitrile (mobile phase B) and H_2_O (mobile phase A) containing 0.05% formic acid. Samples (5 μL) were eluted at a flow rate of 0.245 mL/min, and the gradient program was set as 5%–60%–60%–5% B according to 0–3.5–4.2–5 min. Absorbance was monitored at 220 nm. Hippuric acid was identified and quantified through its standard curve. The IC_50_ value represents the concentration of PBMA hydrolysates when inhibiting ACE activity by 50%.

### 2.7. Desalting Protocol, Cell Culture and Cytotoxicity

Before incubation with A7r5 cells, beef and PBMA hydrolysates were desalted according to the method described previously by Fan et al. [18]. Briefly, beef and PBMA hydrolysates were dissolved in ddH_2_O and then loaded into a Sep-Pak 35cc tC18 cartridge (Waters, MA, USA). Firstly, the cartridge was washed with ddH_2_O at the volume of two column volumes for salt removal. Subsequently, ACN was added to wash the cartridge, and the ACN eluent was collected, vacuum evaporated and freeze-dried.

A7r5 cells were cultured with DMEM medium containing 10% FBS, 25 mM HEPES and 1% penicillin-streptomycin in a cell incubator at 37 °C, 5% CO_2_ and 100% humidity. The culture media were changed every two days. The cytotoxicity of beef and PBMA hydrolysates against A7r5 cells was measured through an alamarBlue assay, as depicted by Fan et al. [18]. A7r5 cells were initially sown in a 96-well plate, and cells were treated with 1.0 mg/mL of beef and PBMA hydrolysates for 24 h when reaching 80% of confluency, and then the medium was replaced with 200 µL of 10% alamarBlue solution for another 4 h. Finally, the solution (150 µL) was transferred into an opaque 96-well plate for fluorescence signal detection, with an emission wavelength at 590 nm and excitation wavelength at 560 nm.

### 2.8. Superoxide Detection

Superoxide in A7r5 cells was investigated by the Dihydroethidium (DHE) staining method [23]. A7r5 cells were pre-incubated with hydrolysates (1.0 mg/mL) for 1 h before the addition of Ang II (1 µM) for 0.5 h. Subsequently, DHE (20 µM) was added and treated for another 30 min. After that, cells were triple-washed with non-phenol-red DMEM, and the fluorescence intensity was measured by an Olympus IX81 fluorescent microscope (Olympus, Tokyo, Japan). Each data was comprised of two or three random fields. The mean fluorescence intensity was obtained using ImageJ software (National institutes of health, Bethesda, MD, USA).

### 2.9. Western Blotting

A7r5 cells were pre-incubated with beef and PBMA hydrolysates (1.0 mg/mL) for 1 h before adding Ang II (1 µM) for 24 h. After the treatment, cells were scraped and lysed in boiling Laemmle’s buffer containing 50 mM DTT and 0.2% Triton-X-100, and then cell samples were loaded onto a 9% separating gel and transferred to a nitrocellulose membrane for specific antibodies incubation. Bands of cyclooxygenase-2 (COX-2; Abcam, Toronto, ON, Canada) and inducible nitric oxide synthase (iNOS; BD Biosciences, San Jose, CA, USA) were normalized to GAPDH (ab8245, Abcam). The fluorescent bands were visualized by adding corresponding secondary antibodies, and the signals were detected using Licor Odyssey BioImager (Licor Biosciences, Lincoln, NE, USA).

### 2.10. Statistical Analysis

SPSS 17.0 (SPSS Inc., Chicago, IL, USA) was applied to statistical treatment with ANOVA analysis followed by the Duncan post hoc test. Data were expressed as mean ± standard deviation. Differences were considered statistically significant at *p* < 0.05.

## 3. Results and Discussion

### 3.1. Molecular Weight Distribution, DH and Amino Acid Compositions of Beef and PBMA Digests

**Figure 1:**
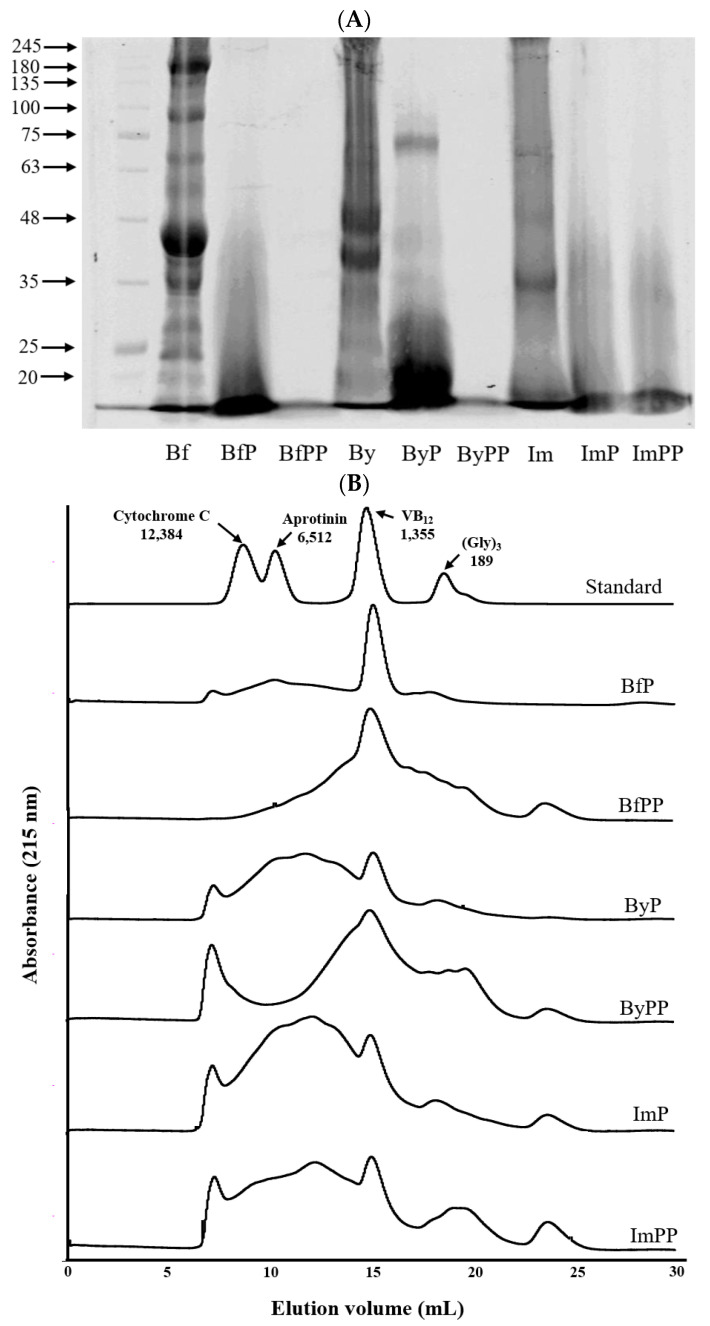
SDS-PAGE (**A**) and size exclusion chromatogram (**B**) of beef and PBMA hydrolysates. Bf = cooked beef patty; BfP = cooked beef patty after pepsin digestion; BfPP = cooked beef patty after pepsin and pancreatin digestion; By = cooked Beyond patty; ByP = cooked Beyond patty after pepsin digestion; ByPP = cooked Beyond patty after pepsin and pancreatin digestion; Im = cooked Impossible patty; ImP = cooked Impossible patty after pepsin digestion; ImPP = cooked Impossible patty after pepsin and pancreatin digestion.

Figure 1A shows that the pepsin and/or pancreatin treatments cause a substantial decrease/disappearance in the intensity of large-molecular-weight protein bands, which is due to the degradation of proteins into peptides/free amino acids. Likewise, the results of size exclusion chromatography further demonstrated that the small-molecular-weight fractions in beef and PBMA hydrolysates increased rapidly from gastric digestion to the intestinal digestion phase (Figure 1B), which dominated peptide composition in BfPP, ByPP and ImPP due to further extensive hydrolysis. Furthermore, DH data were consistent with the results shown in SDS-PAGE and size exclusion chromatography (Table 1). The DH of ByPP and ImPP increased gradually during in vitro digestion, being 4.92% and 6.09% after gastric digestion, and further increased to 7.94% and 7.48% after intestinal digestion, respectively. Beef hydrolysate had higher DH than PBMA throughout digestion. The gastrointestinal digestion fate of real meat and PBMA are hypothesized to be different due to the diversities in the structures and compositions of the raw material. Particularly, PBMA contains different sources of proteins as compared with real meat, as well as a variety of food additives which may affect protein digestion [24,25]. Moreover, the processing technologies in PBMA production may result in the formation of structures that negatively impact protein digestion. For instance, the dense mesh structure or aligned fibrils of proteins formation under the thermal–mechanical treatment largely impair the digestibility of proteins in PBMA [26]. A better swelling capacity of beef promotes penetration of gastrointestinal proteases, whereas the bulkiness of storage proteins, protein aggregates and the presence of antinutritional factors in beans limit the digestion of PBMA [27]. Our results are consistent with previous research. For instance, Xie et al. demonstrated that real pork and beef showed higher digestibility than PBMA [13], and the study of McClements et al. also reported the inferior digestibility of PBMA [8].

**Figure 2:**
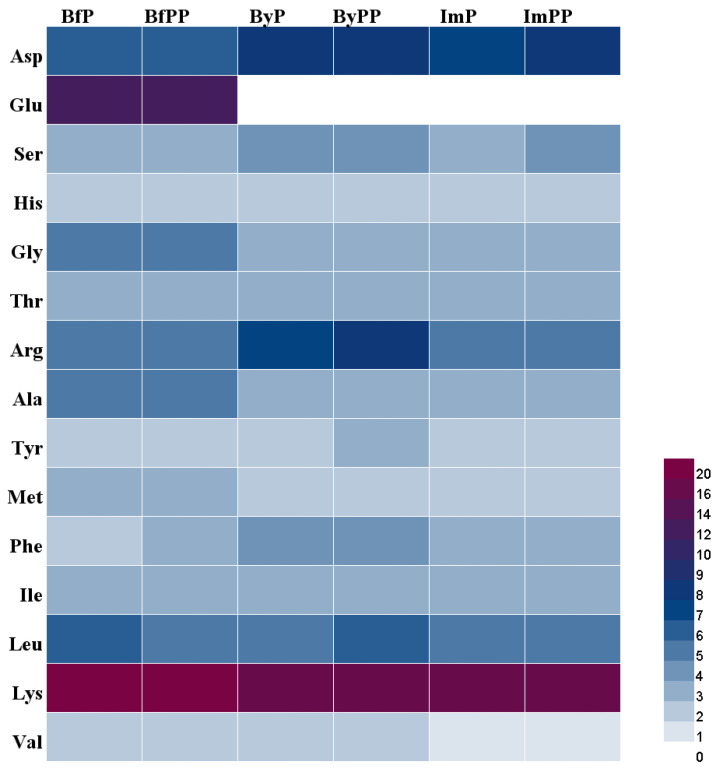
The amino acid compositions of beef and PBMA hydrolysates.

Amino acid composition is an indicator of the nutritional value of protein hydrolysates [28]. Essential amino acids (EAA) refer to amino acids which cannot be auto-synthesized by the human body, or the rate of synthesis is inadequate to meet the biological needs of the body. Thus, they need to be supplied by food protein intake. Normally, Val, Leu, Ile, Phe, Lys, His, Thr and Met are considered the eight EAA of individuals. Table 1 and Figure 2 shows that the total amino acid compositions of the three hydrolysates ranged from 67.56–87.64 g/100 g. Gastrointestinal digestion of beef had the highest content of amino acids, whereas ByPP and ImPP had a relatively low content of amino acids. However, the content of EAA in PBMA hydrolysate was comparable to the beef counterpart. The contents of EAA in ByPP and ImPP were 42.91 g/100 g and 41.60 g/100 g, whereas a higher value (46.04 g/100 g) was found in BfPP. EAA cannot be synthesized by mammals and must be obtained from food. EAA have important regulatory effects in many physiological events [29,30]. On the other hand, PBMA hydrolysates also contain a high level of non-EAA. Of which, Glu, Gly and Ala were abundant in beef hydrolysate, whereas Asp and Arg content was lower. In particular, no Glu was detected in PBMA hydrolysates. There is no compelling evidence to support that synthesis of non-EAA in the body could satisfy the requirement of physiological activities [31]. Thus, the content of non-EAA should still be taken into consideration when evaluating the nutritional value of proteins. From the amino acids profile, PBMA hydrolysates were expected to possess comparable nutritional properties to that of beef hydrolysate.

### 3.2. Effects of Gastrointestinal Digestion on Peptide Profiles of Beef and PBMA Hydrolysates

LC-MS/MS was used to identify the peptide profiles of beef and PBMA hydrolysates in this study, with the purpose of following the generation of peptides during in vitro gastrointestinal digestion and their relationship with bioactivities. To identify the potential bioactive peptides and predict their chemical properties, peptidomics and bioinformatics approaches were applied. Additionally, a peptide fragment may recur multiple times in its parent protein sequences, which can impact the theoretical content of peptides; therefore, this variation was also considered.

**Figure 3:**
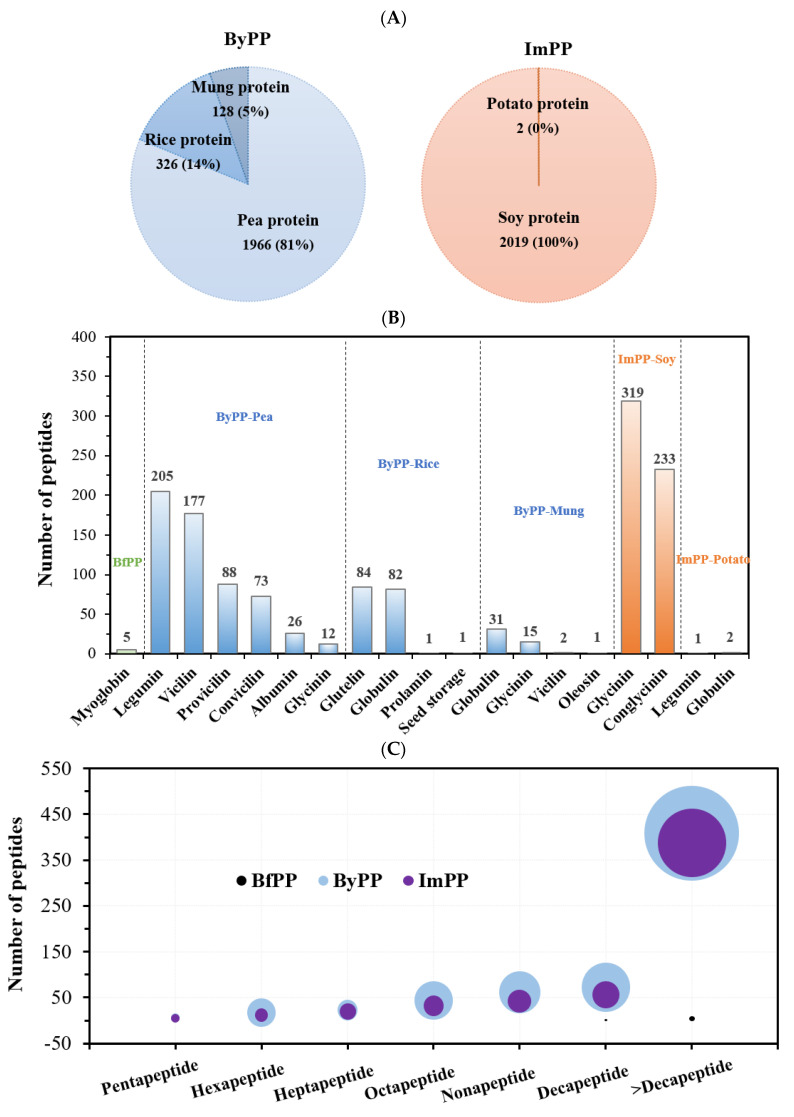

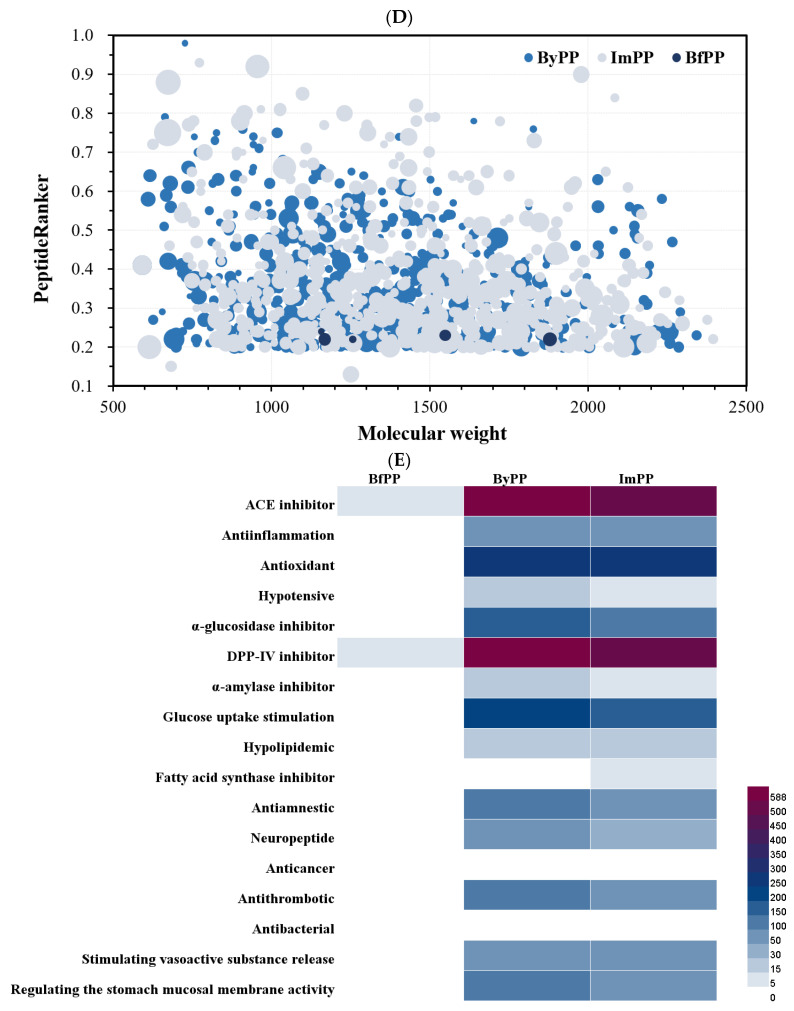
The peptide profile and virtual prediction of PBMA hydrolysates. (**A**) The distribution of peptides identified from BfPP, ByPP and ImPP. (**B**) Distribution of the potent peptides according to their origin proteins in BfPP, ByPP and ImPP. (**C**) Number of potent peptides fragment and their repetitions of each type released, and the bubble size represents the repeat numbers. (**D**) Molecular weight and PeptideRanker score of potent peptides, and the bubble size represents the CPPpred. (**E**) Heat map of the biological function of potent peptides from BfPP, ByPP and ImPP.

A total of 37, 2420 and 2021 peptides were identified in BfPP, ByPP and ImPP, respectively, indicating that gastrointestinal digestion had a significant impact on peptide release (Figure 3A). Among them, the abundant peptide fragments in ByPP were mainly derived from pea protein (81%), followed by rice protein (14%) and mung protein (5%). Almost all peptides identified in ImPP originated from soy protein. These results were consistent with the declaration of protein origins in their formulas. Even though beef hydrolysate had the highest DH, surprisingly, much fewer peptides were identified therein. This is probably because beef protein is more easily digested into free amino acids by gastrointestinal proteases or beef-derived peptides showing stronger hydrophilic properties, which were washed away from the reverse phase column prior to sequence identification. It is worth noting that the amino acid composition among proteins largely dictates the extent of digestion, such as Phe, Tyr, Trp Lys, and Arg, which are the cleavage sites of gastrointestinal proteases [32]. Unfortunately, the peptide fragments released from in silico hydrolysis (pepsin and trypsin) in Supplementary Table S1 show a weak correlation with peptides identified by LC-MS/MS, suggesting the gaps between in silico hydrolysis and actual enzymatic hydrolysis. Particularly, in silico mimic hydrolysis is performed under ideal conditions where all proteins are fully digested, whereas the food matrix and processing conditions have a major impact on the digestibility of food proteins. Similarly, discrepancies between virtual and actual hydrolysis were also reported by others [33,34].

Generally, it is normally accepted that small peptides in protein hydrolysates possess better biological activities [16,24]. PeptideRanker is widely used to predict the potential bioactivity of peptides. A total of 5, 798 and 555 potent peptides were selected from BfPP, ByPP and ImPP based on the following filter conditions: peptide length < 20, molecular weight <3 kDa and PeptideRanker scores >0.2. Parent proteins, peptide sequences, repeat numbers, PeptideRanker scores, CPPpred scores, potential bioactive peptides and biological function of these potent peptides are listed in Supplementary Tables S2–S7. Additionally, Figure 3B shows the distribution of selected peptides in each sample according to their protein origins. Globulin, including legumin and vicilin, is one of the major storage proteins in peas [10]. Almost half of the peptides that occurred in ByPP were from legumin and vicilin in peas. The remaining half was also derived from other storage proteins such as provicilin and convicilin in peas, glutelin and globulin in rice and globulin and glycinin in mung. On the other hand, peptides identified from ImPP were mostly derived from glycinin and conglycinin.

Additionally, the number of small peptides (peptide length < 10) released by gastrointestinal proteases were 222 and 166 in ByPP and ImPP, accounting for 35.24% and 29.96% of the total peptides identified, respectively. Peptides released in ByPP were repeated more frequently than those in ImPP. The potential bioactivities of peptides were predicted by calculating molecular weight, PeptideRanker scores and CPPpred scores. PeptideRanker is used to predict peptide bioactivities, and CPPpred predicts the ability of a peptide to go across the cell membrane [34]. As shown in Figure 3D, peptides in ImPP have higher PeptideRanker scores than those in ByPP. Additionally, most peptides in ByPP and ImPP had strong cell penetration capacity. These results indicated that gastrointestinal digestion could effectively release bioactive peptides from PBMA.

Recently, lifestyle-related chronic diseases have triggered a series of global public health concerns, leading to growing interest in researching food bioactives, including bioactive peptides, as alternatives for treatment. To further clarify and predict the potential biological functions of beef and PBMA hydrolysates, the screened peptides with active probability were compared to the reported active sequences in the BIOPEP database (Supplementary Tables S2–S7). Peptides shared the same sequence with the reported bioactive sequences in the BIOPEP database, implying that they exhibit the same biological functions. Bioactive peptides in PBMA hydrolysates were predicted to exert a wide range of regulatory roles, including amelioration of cardiovascular diseases (including hypertension, diabetes, obesity and hyperlipemia), antioxidation, anti-inflammation, anticancer and neuroprotection (Figure 3E). Taken together, our results suggest that PBMA is a good precursor of bioactive peptides with various biological functions.

### 3.3. ACE Inhibition, Antioxidant and Anti-Inflammation of Beef and PBMA Hydrolysates

After predicting the bioactivities of peptides identified from beef and PBMA digests, we further determined ACE inhibitory, antioxidant and anti-inflammatory activities. ACE is a target of blood pressure reduction [35], and amelioration of oxidative stress and inflammatory responses have been considered key preventive strategies against various chronic diseases [36,37,38].

**Figure 4:**
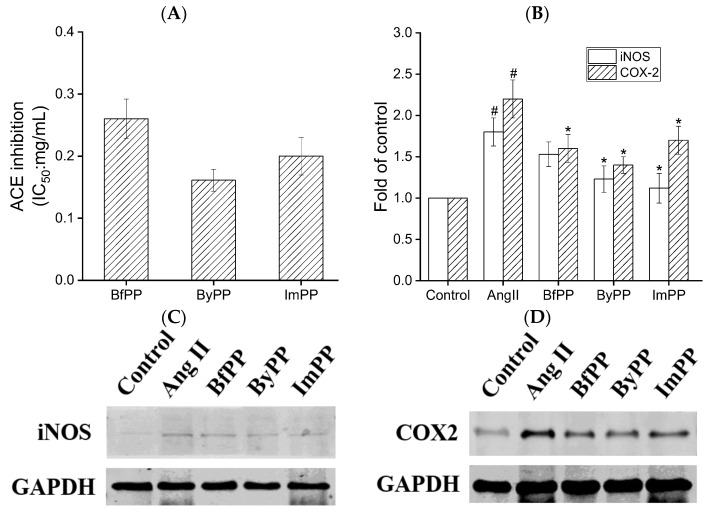
Effect of beef and PBMA hydrolysates prepared by gastrointestinal digestion on ACE inhibitory and anti-inflammatory activities. (**A**) In vitro ACE inhibition, and (**B**–**D**) the expressions of iNOS and COX-2 after co-treatment with 1 μM Ang II and hydrolysates for 24 h in A7r5 cells. The data are represented as means ± SD; *^#^* represents *p* < 0.05 vs. control group. * represents *p* < 0.05 vs. Ang II group.

Hypertension is widely known as a risk factor for cardiovascular diseases, and the renin–angiotensin system (RAS) plays a pivotal role in blood pressure regulation [39]. ACE activates the RAS and converts angiotensin (Ang) I into Ang II, which is a potent vasoconstrictor to trigger hypertension. Figure 4 shows in vitro ACE inhibition of beef and PBMA digests. ByPP showed the highest ACE inhibition, with an IC_50_ value of 0.16 ± 0.03 mg/mL, followed by that of ImPP and BfPP (IC_50_: 0.20 ± 0.05 and 0.26 ± 0.05 mg/mL, respectively). Evidently, the results of ACE inhibition were consistent with the biological function prediction by in silico approach (Figure 3E). Similarly, a previous study also showed that PBMA-derived digests showed ACE inhibitory activity [13].

**Figure 5:**
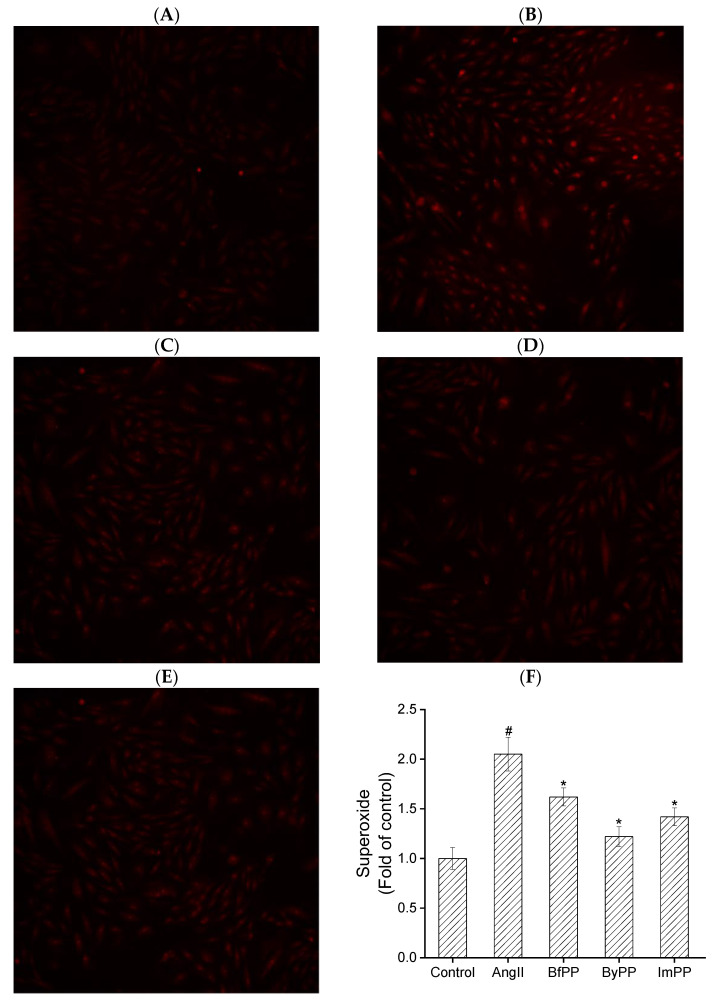
Effect of beef and PBMA hydrolysates prepared by gastrointestinal digestion on antioxidant capacity in A7r5 cells. (**A**) Control, (**B**) AngII, (**C**) BfPP, (**D**) ByPP and (**E**) ImPP, (**F**) oxidative stress in A7r5. The data are represented as means ± SD; ^#^ represents *p* < 0.05 vs. control group. * represents *p* < 0.05 vs. Ang II group.

Oxidative stress triggers various kinds of damage to cells and further disrupts cellular function [40]. Sustained and aberrant oxidative stress contributes to vascular dysfunction, thereby causing hypertension, type 2 diabetes, atherosclerosis and other chronic diseases [41]. Vascular smooth muscle cells (A7r5) are a well-established model for evaluating health benefits, including relief of vascular dysfunction, anti-inflammation and antioxidation. In this study, antioxidant and anti-inflammatory activities in Ang II-induced A7r5 cells were studied. All hydrolysates showed no cytotoxicity against A7r5 cells. Treatment of beef and PBMA hydrolysates significantly lowered superoxide levels in Ang II-stimulated A7r5 cells, especially for ByPP and ImPP (Figure 5). Fan et al. found that spent hen-derived peptides exhibited antioxidant effects by acting as direct radical scavengers or mediating endogenous antioxidant enzymes in Ang II-stimulated A7r5 cells [42]. Similarly, egg white-derived peptide IRW was also demonstrated to exhibit an antioxidant effect in A7r5 cells against Ang II stimulation [39]. In our study, the remarkable inhibition of superoxide generation (*p* < 0.05) in A7r5 cells indicated that PBMA was a good precursor of antioxidant peptides.

Vascular inflammation is an underlying cause of hypertension and cardiovascular diseases. COX2 and iNOS are two proinflammatory mediators in vascular smooth muscle cells [38]; thus, the expression of these two proteins in A7r5 cells was detected to evaluate the anti-inflammatory activity of beef and PBMA hydrolysates. As shown in Figure 4, iNOS and COX2 expression levels surged in A7r5 cells upon Ang II insult (*p* < 0.05), whereas the hydrolysates treatment significantly inhibited their protein expressions. Similarly, peptides VVHPKESF and IRW could attenuate Ang II-induced inflammation in A7r5 cells [43,44]. These findings suggested the formation of anti-inflammatory peptides by gastrointestinal digestion from PBMA.

## 4. Conclusions

This study mimicked the protein digestion of beef and PBMA through an in vitro gastrointestinal tract and further investigated the peptide profile and biological bioactivity by combining peptidomics, bioinformatics and wet lab experiments. Results obtained in SDS-PAGE, size exclusion chromatography and DH showed that gastrointestinal proteases were able to degrade beef and PBMA proteins. Notably, PBMA protein exhibited inferior digestibility than that of beef, as reported previously. From the amino acids profile, PBMA hydrolysates were expected to possess comparable nutritional properties to beef hydrolysate. A total of 37, 2420 and 2021 peptides were identified in the gastrointestinal digests of beef, Beyond Meat and Impossible Meat, respectively. The astonishingly fewer peptides identified from beef digest is probably due to the near-full digestion of beef proteins. The analysis of peptide profiles indicated that PBMA could be considered a good precursor of bioactive peptides with widespread biological functions, including amelioration of cardiovascular diseases (including hypertension, diabetes, obesity and hyperlipemia), antioxidation, anti-inflammation, anticancer and neuroprotection. Furthermore, PBMA hydrolysates exhibited great ACE inhibition, antioxidant and anti-inflammation in test tube experiments and A7r5 cells. The current results underscored the promise of generating bioactive peptides from PBMA.

## Figures and Tables

**Table 1:** The degree of hydrolysis (DH) and amino acid compositions of beef and PBMA hydrolysates.

	BfP	BfPP	ByP	ByPP	ImP	ImPP
DH (%)	8.26 ± 0.67	10.66 ± 0.83	4.92 ± 0.11	7.94 ± 0.73	6.09 ± 0.59	7.48 ± 0.40
Amino acids composition (g/100 g)						
Total amino acids	87.61 ± 1.51 ^a^	87.64 ± 1.43 ^a^	70.80 ± 1.26 ^b^	75.11 ± 1.73 ^c^	67.56 ± 1.20 ^d^	69.35 ± 0.54 ^d^
EAA	45.75 ± 0.84 ^a^	46.04 ± 1.13 ^a^	40.76 ± 0.53 ^b^	42.91 ± 1.28 ^c^	40.61 ± 0.71 ^b^	41.60 ± 1.34 ^b,c^

The data are represented as means ± SD; Values with different letters (a–d) within the same row indicate significant the differences.

## Data Availability

All related data and methods are presented in this paper. Additional inquiries should be addressed to the corresponding author.

## Supplementary Materials

The following supporting information can be downloaded at: https://www.mdpi.com/article/10.3390/foods12051061/s1. Table S1: Prediction of peptides released by gastrointestinal proteases through in silico hydrolysis of PBMA. Tables S2–S7: Potential bioactive peptides in BfPP, ByPP-pea protein, ByPP-rice protein, ByPP-mungbean protein, ImPP-soy protein and ImPP-potato protein, respectively.
