# Supplementary material for: Peptidomics Study of Plant-Based Meat Analogs as a Source of Bioactive Peptides
Source: Foods. 2023 Mar 2;12(5):1061. doi: 10.3390/foods12051061 (PMC10000916; doi:10.3390/foods12051061)
Supplement: Supplementary file 1 [file foods-12-01061-s001.zip › foods-2224317-supplementary.pdf]

Table S1. Prediction of peptides released by gastrointestinal proteases through *in silico* hydrolysis of PBMA.

Materials	Peptide fragments
Beef	VL, GF, HG, IR, EK, DK, HL, HK, YL, EF, AL, EL, MGL, TAL, HAK, GGIL, IPVK, ASEDL, HPSDF, NAWGK, TEAEMK, HGNTVL, SDGEWQL, TGHPETL, AESHANK, NDMAAQYK, GHHEAEVK, ISDAIIHVL, GADAQAAMSK, VEADVAGHGQEVL, DL, SL, SR, NL, EF, DF, HF, NF, NK, GL, QL, EL, YK, AF, PTR, DSR, SSR, VPL, QSL, EPF, DGR, VYF, NVK, ADK, VVR, AQK, AIK, AVK, EGL, IHL, APK, DVK, GNK, EEK, MSIF, DQVF, HPEL, SIPL, SPEK, VWAK, IDWEF, PQMAL, DWAER, ESVMK, VDPHF, PPSVDR, TAMDNAF, DAYEVGK, MVVEAPL, AEEITIK, EMSAIQPR, EYTQPGEL, HDDIVNMHR, VYVHGVTGK, TQAEIVDGL, SVACGDAAMSESVGR, NHIQATITTDDVL, VIEAPVNEPNYK, AIQPSEVQER, EIVTAEDGSK, TENASHSEHR, VTPEVVDASK, VR, EL, YR, DL, ER, QK, GR, DK, ML, AK, SK, TK, TR, AL, QR, SR, NR, NL, EK, TL, DR, QL, VK, QF, EL, VR, AR, MK, ISR, ESR, EDL, DAR, AER, DEL, AEK, SQL, GQL, EGL, AAL, DDL, SSL, QAR, TVR, EEL, MAK, EIK, THR, TDR, ENR, ETK, SAL, TDL, EAL, SQL, DNL, NHL, ENK, ENL, AQR, IAL, SQL, SDR, ADR, EAF, YER, QNR, MIL, QDL, AYK, ATR, GNR, EGR, TIIR, DSQL, MSESHVK, GTSPSTVR, NANEMSIQL, DTMAER, SGTSSQTHDAIR, DMEISK, ENANAAF, ETAEATL, HNTMISEISSEVENL, EIDNVL, DGAL, ASAESK, TDDL, QMADANSAK, AAENF, ANQEYEAQVVTF, ESQL, AMDEDAR, QTQL, QMDYDNL, YEEEAEAAGNL, NQVAK, NADMAAL, TEEF, ITEL, EDMAEHER, ANNL, QAENEAL, AAENGEL, QAENL, ANEL, IDEMTVEINTL, NSANSAL, EADNMR, GQVGDL, IANL, GDQL, DANR, EAER, ASAL, HDAEEAL, EMEAK, YVASQNAL, SEMEQR, DEEL, STTR, TIEEL, TTTISEMEVR, SDMSR, YEATISEL, EVQL, DVANK, ANANL, VQEL, QAAL, EDER, EAAESNL, QVSER, ASEVEEIR, NAESEL, NDANGR, ISEL, SVNTL, TNDK, EGDIGVMQGDL, DEAVNAR, AAEDR, NAEVL, ADEL, QEQENYXR, AETL, EIEIR, EITVK, EEAEAF, MVQK, EAEL, DGEIR, ANAR, QTQSEDDK , TQIK, EEQEEVSQL, TMSK, AQQQIEEAEHR, ADMAER, TITIK, TIGGPGSR, AVSVVR, EINSVSR, ATSIM, ML, GK, NK, TIK, VK, VTTAESQL, EF, EMQK, NAL, GQDL, DQVVR, TIGL, EVWYF, GIQYVDK, DGNPTF, DK, IL, SNDF, APGSEL, DF, MVK, YPENVEEEL, IQTCTITHF, YL, QVK, SDIMSGK, IYCPTDTAVL, ASYACVAK, YGPYDPQSCPK, SL, PIDR, IPGK, DQYDQTDEQWF, DR, IVTYYK, DHHDMSR, EDAMVQYL, QIAQDL, EMYGVETF, NIK, NK, GTSL, VL, GVDAL, GL, SIYEPDNL, DPK, IGF, PWSEIR, NL, SF, HDK, IIK, PADK, SAK, EF, YF , VEK, SK, INK, IL, AL, CTGNHEL, YMR, SDSIEVQQMK, IQAK, EER, EL, EAEK, QR, EER, QR, MENEQK, QEL, AQML, QK, ESDL, ADMK, ASAYEGK, IAEL, ESL, NQER, HAR, ESL, QK, SQDK, AEMNR, EETEASVEER, NR, VAER, DR, VQR, EVETQK, VAMAAMEAEK, VQAETEIR, IMR, EK, HDAQR, NPNVNGSGDAASQDDESEAK, EL, EVIPDAK, IEETR, VTMVSK, NESL, QTK, ANL, EL, SSTR, DPSK, MR, DIDR, HHEYNVR, EGNDK, YK, TL, NIR, GNTMCR, VEQF, ESM, MK, PK, NQSPTK, IQAEIK, TL, EK, NK, TPR, WK, HNTDK, NPGGDK, NPGGDK, NPGENTEHDK, NPGGDK, NPEEIK, TQ, MSAF, SF, HR, TL, CSASTL, SL, WAF, QACVL, CL, ISAIL, DSSIWL, WVVVEPSEL, GPL, GVSGPL, GVSGPL, GVSGPL, GVSGPL, GVSGPL, GVSGPF, CF, QVP, MK, TQSQAK, IPGGNTEPGK, NPGGNTEPGK, NPGEEMNPGNNMTPGSNSENGNNQNTPIK, SL, PAIISNSNQQL, GEINK, NDGQSIL, GAL, EK, NR, SL, NL, DK, SQL, DVK, NIK, TASAIVF, AK, NSTK,

	YEGQIL, VSF, VK, NHNSESTAK, NK, ML, GPL, GVSGPL, GVSGPL, GVSGPL, GVSGPL, GVSGPL, GVSGL, CF, MMK, WNWK, IK, NL, TK, NYQK, IEGEL, TEDEEK, IDK, NL, YNGL, TQDENHIWGEDDDL, DTPANSGTK, NQGTGAPK, SPQQGTGAPK, SPQQGTGAPK, APQQGTGAPK, APQQGTGAPK, APQQGTGAPK, APQQGTGAPK, APQQGTGAPK, SPQQGAETNSGK
Beyond meat	MAK, AL, SL, SF, CF, GGCF, AL, EQPQQNECQL, ER, DAL, EPDNR, IESEGGL, IETWNPNNK, QF, CAGVAL, SR, ATL, QR, NAL, PYYSNAPQEIF, IQQGNGYF, GMVF, PGCPETF, EEPQESEQGEGR, YR, DR, HQK, VNR, EGDIIAVPTGIVF, WMYNDQDTPVIAVSL, TDIR, SSNNQL, DQMPR, YL, AGNHEQEF, QYQHQQGGK, QEQENEGNNIF, SGF, DYL, EDAF, NVNR, HIVDR, QGR, NEDEEK, GAIVK, VK, GGL, SIISPPEK, QAR, HQR, GSR, QEEDEDEEK, QPR, HQR, GSR, QEEEEDEDEER, QPR, HQR, GEEEEEDK, GGSQK, GK, SR, QGDNGL, EETVCTAK, NIGPSSSPDIYNPEAGR, IK, TVTSL, DL, PVL, WL, SAEHGSL, HK, NAMF, VPHYNL, NANSIIYAL, GR, AR, QVVNCNGNTVF, DGEL, EAGR, AL, TVPQNYAVAAK, SL, SDR, SYVAF, TNDR, AGIAR, AGTSSVINNL, PL, DVVAATF, NL, QR, NEAR, QL, SNNPF, VPAR, ESENR, ASA, MATK , AL, SL, SF, CF, GGCF, AL, EQPEQNECQL, ER, NAL, EPDNR, IESEGGL, IETWNPNNK, QF, CAGVAL, SR, ATL, QHNAL, PYYSNAPQEIF, IQQGNGYF, GMVF, PGCPETF, EEPQESEQGEGR, YR, DR, HQK, VNR, EGDIIAVPTGIVF, WMYNDQDTPVIAVSL, TDIR, SSNNQL, DQMPR, YL, AGNHEQEF, YQHQQGGK, QEQENEGNNIF, SGF, DF, EDAF, NVNR, HIVDR, QGR, NEDEEK, GAIVK, VK, GGL, SIISPPEK, QAR, HQR, GSR, QEEDEDEDEER, QPR, HQR, GSR, QEEEEDEDEER, QPR, HQR, GEEEEEDK, ER, GSQK, GK, SR, QGDNGL, EETVCTAK, NIGPSSSPDIYNPEAGR, IK, TVTSL, DL, PVL, WL, SAEHGSL, HK, NAMF, VPHYNL, NANSIIYAL, GR, AR, QVVNCNGNTVF, DGEL, EAGR, AL, TVPQNYAVAAK, SL, SDR, SYVAF, TNDR, AGIAR, AGTSSVINNL, PL, DVVAATF, NL, QR, NEAR, QL, SNNPF, VPAR, QSENR, ASA, MSK, PF, SL, SL, ASACL, ATSSEF, DR, NQCQL, DSINAL, EPDHR, VESEAGL, TETWNPNHPEL, CAGVSL, IR, TIDPNGL, HL, PSF, SPSPQL, IF, IIQGK, GVL, GL, SF, PGCPETYEEPR, SSQSR, QESR, QQQGDSHQK, VR, GDIIAIPSGIPYWTYNHGDEPL, VAISL, DTSNIANQL, DSTPR, VF, YL, GGNPETEF, PETQEEQQGR, HR, QK, HSYPVGR, SGHHQQEEESEEQNEGNSVL, SGF, SSEF, AQTF, NTEEDTAK, SPR, DER, SQIVR, VEGGL, IIK, PK, GK, EEEEK, EQSHSHSHR, EEK,EEEEEEEEDEEEK, QR, SEER, NGL, EETICSAK, IR, ENIADAAR, ADL, YNPR, AGR, ISTANSL, TL, PVL, YL, SAEYVR, YR, NGIYAPHWNINANSL, YVIR, GEGR, VR, IVNCQGNTVF, DNK, VR, GQL, VVVPQNF, VVAEQAGEEEGL, EYVVF, TNDR, AAVSHVQQVF, ATPSEVL, ANAF, GL, QR, QVTEL, SGNR, GPL, VHPR, SQSQSH, IPYWTYNHGDEPL, VAISL, DTSNIANQL, DSTPR, VF, YL, GGNPETEF, PETQEEQQGR, HR, QK, HSYPVGR, SGHHQQEEESEEQNEGNSVL, SGVSSEF, AQTF, NTEEDTAK, SPR, DER, SQIVR, VEGGL, IINPK, GK, EEEEEK, EQSHSHSHR, EEEEEEEEDEEK, QR, SEER, NGL, EETICSAK, IR, ENIADAAGADL, YNPR, AGR, IR, TANSL, TL, PVL, YL, SAEYVR, YR, NGIYAPHWNINANSL, YVIR, GEGR, VR, IVNF, QGDAVF, DNK, VR, GQL, VVVPQNF, VVAEQAGEEEGL, EYVVF, TNDR, AAVSHVQQVL, ATPAEVL, ANAF, GL, QR, QVTEL, SGNR, GPL, VHPQSQSQSH, GNSVL, SGF, NVEF, AHSL, NTK, EDTAK, SPQDER, GQIVK, VEDGL, HIISPEL, QEEEEQSHSQR, K, EEEEEEQEQR, HR, HSK, EDEDEDEEEEEER, EQR, HR, HSEK, EEEDEDEPR, SYETR, WK, HTAEK, ESHGQGEEEEEL, EK, EEEEEEEIQR, QHSK, GR, NGL, EETICSAK, IR, ENIAR, PSR, GDL, YNSGAGR, ISTVNSL, TL, PIL, NL, SAEYVL, YR, NGIYAPHWNINANSL, YVIR, GEGR, VR, IVNSEGNK, VF, DDK, VSL, GQL, VVVPQNF,

VVAQQAGNEEGF, EYVVF, TNDR, AAVSHVNQVF, ATPGEVL, ANAF, GL, HSQVAQIK, SNGNR, GPL, VQPQSQ, KGGL, IISPPEKQAR, HQR, GSQKGKSR, QGDNGL, EETVCTAKL, NIGPSSSPDIYNPEAGR, IKTVTSL, DL, PVL, WL, KL, SAEHGSL, HKNTMF, VPHYNL, NANSIIYAL, KGR, AR, QVVNCNGNTVF, DGKL, EAGR, AL, TVPQNYAVAAKSL, NDR, SYVAF, KTNDR, AGIAR, AGTSSVINDL, PL, DVVAATF, KL, QR, DEAR, QL, KSNNPF, KF, VPAR, QSENR, ASA, MSK, PSL, SL, ASACL, ATR, SEF, DR, NQCQL, DNINAL, EPDHR, VESEAGL, TETWNPNNPEL, CAGVSL, IR, TIDPNGL, HL, PSF, SPSPQL, IF, IIQGK, GVL, GL, PGCPETYEEPR, SSQSR, QGSR, QQQGDSHQK, MAR, HF, SSF, SL, CF, TTACL, AHHSESDR, NQCQL, DTINAL, EPDHR, VESEAGL, TETWNPNHPEL, CAGVSL, IR, TIDPNGL, HL, PSYSPSPQL, IF, IIQGK, GVL, GL, AVPGCPETYEEPR, SQSR, QQQQR, DSHQK, SK, GDVIAIPPGIPYWTYNHGHEPL, VAITL, DTSNTL, NQL, DSTPR, VF, YL, GGNPEIEF, PETQQK, QHEPR, QQR, YSF, VGR, GGQQQEEESEEQNEGNSVL, SGF, NVEF, AHSL, NTK, EDTAK, SPQDER, GQIVK, VEDGL, HIISPEL, QEEEEQSHSQR, EEEEEEQEQR, HR, HSK, EDEDEDEEEEEER, EQR, HR, HSEK, EEEDEDEPR, SYETR, WK, HTAEK, ER, ESHGQGEEEEEL, EK, EEEEEEGIQR, QHSK, GR, NGL, EETICSAK, IR, ENIAR, PSR, GDL, YNSGAGR, ISTVNSL, TL, PIL, NL, SAEYVL, YR, NGIYAPHWNINANSL, YVIR, GEGR, VR, IVNSEGNK, VF, DDK, VSL, GQL, VVVPQNF, VVAQQAGNEEGF, EYVVF, TNDR, AAVSHVNQVF, ATPGEVL, ANAF, GL, HSQVAQIK, SNGNR, GPL, VQPQSQ, MAK, AL, SL, SF, CF, GGCF, AL, EQPQQNECQL, ER, DAL, EPDNR, IESEGGL, IETWNPNNK, QF, CAGVAL, SR, ATL, QR, NAL, PYYSNAPQEIF, IQQGNGYF, GMVF, PGCPETF, EEPQESEQGEGR, YR, DR, HQK, VNR, EGDIIAVPTGIVF, WMYNDQDTPVIAVSL, TDIR, SSNNQL, DQMPR, YL, AGNHEQEF, QYQHQQGGK, QEQENEGNNIF, SGF, DF, EDAF, NVNR, HIVDR, QGR, NEDEEK, GAIVK, VK, GGL, SIISPPEK, QAR, HQR, GSR, QEEDEDEEK, QPR, HQR, GSR, QEEEEDEDEER, QPR, HQR, GEEEEEDK, ER, GGSQK, GK, SR, QGDNGL, EETVCTAK, NIGPSSSPDIYNPEAGR, IK, TVTSL, DL, PVL, WL, SAEHGSL, HK, NAMF, VPHYNL, NANSIIYAL, GR, AR, QVVNCNGNTVF, DGEL, EAGR, AL, TVPQNYAVAAK, SL, SDR, SYVAF, TNDR, AGIAR, AGTSSVINNL, PL, DVVAATF, NL, QR, NEAR, QL, SNNPF, VPAR, ESENR, ASA, FL, EETVCTL, HEDL, AGSSQADVFNPR, AGR, ITSVNSL, TL, PVL, HL, SAQWVK, YK, NGIFMPHWNL, NANS, MR, VQAQQIR, CQVER, DGL, EHPEDPL, IETR, PNNK, QIR, CAGVPL, SR, PTL, NGL, PL, VL, HWSQEIF, MQQPF, WIL, WDGIPR, VVL, AL, SHK, NL, MK, QGTETDK, VTR, EDIHCCSYWYCIL, GCTR, TK, NSSYCR, SYDIR, SSITSL, IR, CPWR, DSIL, GTPSK, EF, QYEHEQGESAF, AF, AAAAL, AF, SPL, DDAAASSSSSK, DIVK, TR, MR, MR, NK, DTATANIAK, MR, MNL, AAMR, VL, GGAR, SR, VGL, CL, SPL, SL, TK, GPF, VSSSF, PL, QEQENEEATTF, QWL, QEDSYPNWYQPGPPSF, GSIEVL, SMVSHK, IAMF, VPL, QPECK, QYNDR, ATK, DR, QEL, SNENVL, VK, VSR, QL, EEL, SK, NAK, SSSR, SVSSESGPF, NL, SEDPL, YSNNSGK, EL, TPEK, NQQL, QDL, DL, VNSVDL, EGSL, PNYNSR, AL, VL, VVNEGK, GDF, EL, VGQR, NENQGK, EN, MAATTMK, ASF, PL, ML, MGISF, ASVCVSSR, SDPQNPF, IF, SNK, QTL, ENENGHIR, QK, DQR, SK, IF, ENL, QNYR, EYK, SK, PHTIF, PQHTDADYIL, VVL, SGK, AIL, TVL, PDDR, NSF, NL, ER, GDTIK, PAGTIAYL, VNR, DDNEEL, VL, DL, AIPVNR, PGQL, QSF, SGNQNQQNYL, SGF, SK, NIL, EASF, NTDYEEIEK, VL, EEHEK, ETQHR, SL, DK, QQSQEENVIVK, SR, GQIEEL, SK, NAK, STSK, SVSSESEPF, NL, SR, GPIYSNEF, GK, EITPEK, NPQL, QDL, DIF, VNSVEIK, EGSL, PHYNSR, AIVIVTVNEGK, GDF, EL, VGQR, NENQQEQR, EDDEEEEQGEEEINK, QVQNYK, AK, SSGDVF, VIPAGHPVAVK, ASSNL, DL, GF,

GINAENNQR, NF, AGDEDNVISQIQR, PVK, EL, AF, PGSAQEVDR, IL, ENQK, QSHF, ADAQPQQR, ER, GSR, ETR, DR, SSV, MAATPIK, PL, ML, AIAF, ASVCVSSR, SDQENPF, IF, SNR, QTL, YENENGHIR, QK, DK, SK, IF, ENL, QNYR, EYK, SK, PHTL, PQYTDADF, IL, VVL, SGK, ATL, TVL, SNDR, NSF, NL, ER, GDAIK, PAGTIAYL, ANR, DDNEDL, VL, DL, AIPVNK, PGQL, QSF, SGTQNQPSL, SGF, SK, NIL, EAAF, NTNYEEIEK, VL, EQQEQEPQHR, SL, DR, QEINEENVIVK, VSR, EQIEEL, SK, NAK, SSSK, SVSSESGPF, NL, SR, NPIYSNK, GK, EITPEK, NQQL, QDL, DIF, VNSVDIK, EGSL, PNYNSR, AIVIVTVTEGK, GDF, EL, VGQR, NENQGK, ENDK, EEEQEEETSK, QVQL, YR, AK, SPGDVF, VIPAGHPVAINASSDL, NL, IGF, GINAENNER, NF, AGEEDNVISQVER, PVK, EL, AF, PGSSHEVDR, NQK, QSYF, ANAQPL, QR, MATTVK, SR, PL, GIIF, ASVCVTYANYDEGSETR, VPGQR, ER, GR, QEGEK, EEK, HGEWR, PSYEK, EEHEEEK, QK, YR, YQR, EK, EQK, EVQPGR, ER, WER, EEDEEQVEEEWR, GSQR, EDPEER, AR, HR, EER, TK, DR, HQR, EGEEEER, SSESQEHR, NPF, SNK, TL, ENENGHIR, QR, DK, SDL, ENL, QNYR, VEYR, AK, PHTIF, PQHIDADL, IL, VVL, NGK, AIL, TVL, SPNDR, NSYNL, ER, GDTIK, IPAGTTSYL, VNQDDEEDL, VVDF, VIPVNR, PGK, EAF, GL, SENK, NQYL, GF, SK, NIL, EASL, NTK, YETIEK, VL, EEQEK, PQQL, DR, TQQGEER, DAIIK, VSR, EQIEEL, AK, SSSK, SL, PSEF, EPF, NL, SHK, PEYSNK, GK, EITPEK, YPQL, QDL, DIL, VSCVEINK, GAL, ML, PHYNSR, AIVVL, VNEGK, GNL, EL, GL, NEQQER, EDR, ER, NNEVQR, YEAR, SPGDVVIIPAGHPVAISASSNL, NL, GF, GINAK, NNQR, NF, SGSDDNVISQIENPVK, EL, TF, PGSSQEVNR, IK, NQK, QSHF, ASAEPEQK, EEESQR, SPL, SSVL, DSF, MATTIK, SR, PL, GIIF, ASVVSVTYANYDEGSEPR, VPAQR, ER, GR, QEGEK, EEK, HGEWR, PSYEK, EEDEEEGQR, ER, GR, QEGEK, EEK, HGEWGPSYEK, QEDEEEK, QK, YR, YQR, EK, EDEEEK, QK, YQYQR, EK, K, EQK, EVQPGR, ER, WER, EEDEEQVDEEWR, GSQR, R, EDPEER, AR, HR, EER, TK, DR, HQR, EGEEEER, SSESQER, NPF, SNK, TL, ENENGHIR, QR, DK, SDL, ENL, QNYR, VEYR, AK, PHTIF, PQHIDADL, IL, VVL, SGK, AIL, TVL, SPNDR, NSYNL, ER, GDTIK, PAGTTSYL, VNQDDEEDL, VDL, VIPVNGPGK, EAF, DL, AK, NK, NQYL, GF, SK, NIL, EASYNTR, YETIEK, VL, EEQEK, DR, QQGEETDAIVK, VS, MAR, PF, IL, SL, SML, ASACF, AF, SF, STF, NF, NK, CQL, DSL, NAL, EPDQR, VESEGGVIETYNSQHPEL, QCAGVTVSK, HTL, YR, NGL, HL, PSYSPYPK, IHVIQGQGAF, GL, AVPGCPETF, EEPAEGSSR, GR, QQHVQDSHQK, IR, QF, TQGDVIL, IPPGIPYWTF, NTGDEPL, IAVSL, YTSSIHNNL, DQSPR, VF, YL, GGNPDIENPETTK, EQPQQQK, GGGR, QQQEEEEEEAGSVL, SGF, SK, HF, AQSF, NIDEDL, AEK, SPEDDR, QIVK, VEGGL, SIISPMGQEQQQQEEEK, EDEDEDEPR, SR, WEEK, EEEEEEEEYETK, HSR, EHGNGIEESICTVK, HESISR, PSR, ADF, YNPK, AGR, ISTL, NSATF, PAL, TPF, GL, SAQYVVL, YK, NGIYAPHWNQNANSVIYVIR, GQGK, VR, VVNNTGDAVF, DR, VL, GQL, VVPQNF, VVAEQASEEGF, EYVVF, TNEMATTTHMK, QVF, AIPAEVL, ANTYNL, QSQVR, EL, YL, GNQGPL, VNPDSQ, MAK, AL, SL, SHCL, VL, SGCF, ASK, DK, TQQPQQNEYECQL, QR, MNAL, PDNR, IESEGGF, IETWNPNSK, PF, QCAGVAL, SR, CTL, QR, NGL, PF, TNAPQQIF, IQQGSGIF, GIIF, SGCPQTF, EEPTQR, GQSQK, PQDR, HQK, VYHF, EGDL, IAVPTGAAF, WMF, NNQDTPVVAVSL, IHTNSL, QNQL, DQMPR, YL, AGNQEQEF, VQYQQSQSQK, GK, QHQEQEEDNEGGNIF, SGF, TPEF, EQAL, NVDR, IVK, NL, QGVNEDEEK, GAIVK, VEGGL, SVTAPPTR, QQQR, AEEEEEDDDDEEEEK, PQK, CK, SK, SR, NGIDETICTMR, HNIGQTPSPDF, NPQAGSITR, ATGL, DF, PAL, SAQF, GSL, NAMF, VPHYNL, NANSIVYAL, EGSAMMQVVNCTGDR, VF, DGEL, QEGQVL, IVPQNF, AVAAK, SQSNNF, YVAF, THDIPVTGTL, AGR, NSL, NAL, PEEVIQHTF, NL, HSEQAR, QIK,

NNNPF, NF, VPPPR, HSQMR, YVA, MNAL, PDNR, IESEGGF, IETWNPNSK, PF, QCAGVAL, SR, CTL, QR, NGL, PF, TNAPQQIF, IQQGSGIF, GIIF, SGCPQTF, EEPTQR, GQSQK, PQDR, HQK, VYHF, EGDL, IAVPTGAAF, WMF, NNQDTPVVAVSL, IHTNSL, QNQL, DQMPR, YL, AGNQEQEF, VQYQQSQSQK, GK, QHQEQEEDNEGGNIF, SGF, TPEF, EQAL, NVDR, IVK, NL, QGVNEDEEK, GAIVK, VEGGL, SVTAPPTR, QQQR, AEEEEEDDDDEEEEK, PQK, SK, SK, SR, NGIDETICTMR, HNIGQTSSPDF, NPQAGSITTATGL, DF, PAL, SAQF, GSL, NAMF, VPHYNL, NANSIVYAL, EGR, AMMQVVNCTGDR, VF, DGEL, QEGQVL, IVPQNF, AVAAK, SQSNNF, YVAF, THDIPVTGTL, AGR, NSL, NAL, PEEVIQHTF, NL, SEQAR, QIK, NNNPF, NF, VPPPR, HSQMR, YVA, ML, AIAF, ASVCVSSR, SDQENPF, IF, SNR, QTL, YENENGHIR, QK, DK, SK, IF, ENL, QNYR, EYK, SK, PHTL, PQYTDADF, IL, VVL, SGK, ATL, TVL, SNDR, NSF, NL, ER, GDAIK, PAGSIAYF, ANR, DDNEEPR, VL, DL, AIPVNK, PGQL, QSF, SGTQNQK, SSL, SGF, SK, NIL, EAAF, NTNYEEIEK, VL, EQQEQEPQHR, SL, DR, QEINEENVIVK, VSR, DQIEEL, SK, NAK, SSSK, SVSSESGPF, NL, SR, NPIYSNK, GK, EITPEK, NQQL, QDL, DIF, VNSVDIK, VGSL, PNYNSR, AIVIVTVTEGK, GDF, EL, VGQR, NENQGK, ENDK, EEEQEEETSK, QVQL, YR, AK, SPGDVF, VIPAGHPVAINASSDL, NL, IGL, GINAENNER, NF, AGEEDNVISQVER, PVK, EL, AF, PGSSHEVDR, DNAEIEK, IL, L, EEHEK, ETHHR, GL, DK, QQSQEK, NVIVK, VSK, QIEEL, SK, NAK, SSSK, SVSSR, SEPF, NL, SSDPIYSNQYGK, EITPK, NPQL, QDL, DIF, VNYVEIK, EGSL, WL, PHYNSR, AIVIVTVNEGK, GDF, EL, VGQR, NENQQGL, EEDDEEEEQR, EEETK, NQVQSYK, AK, TPGDVF, VIPAGHPVAVR, ASSNL, NL, GF, GINAENNQR, NF, AGEEDNVISQIQK, QVK, DL, TF, PGSAQEVDR, ENQK, QSYF, ANAQPQQR, ETR, SQEIK, EHL, YSIL, GAF, MASVK, ASL, IVL, ATL, GMF, TK, NVGAASCNGVCSPF, EMPPCGTSACR, CIPVGL, VIGYCR, NPSGVF, TNDEHPNL, CESDADCR, GSGK, CGHYPNPGIEYGWCF, ASK, SEAEDF, SK, ITQK, DL, SVSTA, MASVK, ASL, IVL, ATL, GMF, TK, NVGAISCNGVCSPF, DIPPCGSPL, CR, CIPAGL, VIGNCR, NPYGVF, TNDEHPNL, CESDADCR, GSGTF, CGHYPNPDIEYGWCF, ASK, SEAEDVF, SK, ITPK, DL, SVSTA, MASVK, ASL, IVL, ATL, GMF, TK, NVGAASCNGVCSPF, EMPPCGTSACR, CIPVGL, F, IGYCR, NPSGVF, ANDEHPNL, CESDADCR, GSGNF, CGHYPNPDIEYGWCF, ASK, SEAEDF, SK, ITPK, DL, SVSTA, MASVK, ASL, MVL, ATL, GMF, TK, NVGAASCNGVCSPF, EMPPCGSSACR, CIPVGL, VVGYCR, HPSGVF, TNDEHPNL, CESDADCR, GSGNF, CGHYPNPDIEYGWCF, ASK, SEAEDF, SK, ITQK, DL, SVSTA, MASVK, ASL, IVL, ATL, GMF, TK, NVGAASCNGVCSPF, EMPPCGTSACR, CIPVGL, VVGYCR, NPSGVF, TNDEHPNL, CESDADCR, GSGNF, CGHYPNPDIEYGWCF, ASK, SEAEDF, SK, ITPK, DL, SVSTA, MASVK, ASL, IVL, ATL, GMF, TK, NVGAASCNGVCSPF, EMPPCGSSACR, CIPVGL, IGYCR, NPSGVF, GNDEHPNL, CESDADCK, GSGNF, CGHYPNPDIEYGWCF, ASK, SEAEDVF, SK, ITPK, DL, SVSTA, TK, PGYINAAF, SSK, NNEAYF, F, INDK, YVL, DYAPGSSR, DK, VL, YGPTPVR, DGF, SL, NQTIF, GSYGIDCSF, DTENNEAF, IF, YENF, CAL, IDYAPHSK, K, DK, IIL, GPK, IADVF, PF, EGTVF, ESGIDAAYR, STR, GK, EVYL, GDQYAR, IDYGSNSMVNK, EIK, SISSGYPCF, NTIF, ESGADAAF, ASHK, TNEVYF, DDHYAR, VK, VTPGGK, AIMDGVR, EIVDYWPSL, DIVPL, MTK, TGYINAAF, SSQNNEAYL, INDK, YVL, DYAPGTSNDK, VL, YGPTPVR, DGF, SL, NQTVF, GSYGVDCSF, DTDNDEAF, IF, YEK, CAL, IDYAPHSNK, DK, IIL, GPK, IADMF, PF, EGTVF, ENGIDAAYR, STR, GK, EVYL, GDQYAR, IDYETNSMVNK, EIK, SIR, NGF, PCF, NTIF, ESGTDAAF, ASHK, TNEVYF, GDYYAR, VTVTPGATDDQIMDGVR, TL, DYWPSL, GIIPL, EN, GSSSCR, CIPWGL, WGTCR,

YPSGSGR, VIK, VGEHPNL, CESHADCMK, ESGSF, CGR, YPNP, RSK, DCRCIPVGL, VVGYCRHPSGIF, RTTDK, HPNL, CESDADCRK, ESGNF, CGHYPNPDIE, MPNYF, TPL, VL, IATF, MF, PMK, VEACDGVCSPF, EMPPCGSTDCR, CVPWGL, VGQCINPTSATVEK, MVK, EHPNL, CQSHVDCMEK, GSGSF, CGR, YPNA, MAYVK, APL, VVF, AAF, SIF, PMMK, GEEECSGICSSF, EMPPCR, SSSCR, CIPVVL, GGNCVDPSSPTIMK, MVEEHSNL, CQSHF, ECMK, GSGSF, CAR, YPNP, MGSVK, APL, AL, ATF, MF, PAK, NVEATDCSGVCSPF, EMPPCGSTDCR, CIPWGL, VGECK, YPTGSER, VTK, MVEK, HPNL, CQSHDDCVHK, ESGSF, CAR, YPNA, MAYVQL, AYL, VVL, ATF, GMF, TQNVEAAECNGVCSPF, EMPPCGSSDCR, CIPVGL, VVGYCR, YPSGL, NTIEEHPNL, CK, SNL, DCIEK, ESGK, CGHYPNP, MAYVR, THL, VVF, STF, SL, PMK, VGATDCSGICSPF, EMPPCR, SSDCR, CIPIVL, VGGYCINPISPAATK, MVK, EHPNL, CHSHTDCTK, GSGSF, CAR, YPNP, RSSDCRCIPIAL, VMGYCRYPSSVSNDAVTRMAK, EHPNL, CQSHADCK, NK, RSGSF, CARYPNP, MEL, DL, TPR, AETMF, EGDGGGYYTWSSSQVPL, AK, NNVCAGR, HPR, GF, AL, PHYADSSK, IGYVIQGTDGVVGL, VL, PNTGEEVVL, GDVIPVPIGSVSWWF, NDGDSDF, IIVF, GETSK, AL, IPGEISYF, TGAL, GIIGGF, STEL, TGK, VYGL, DK, DEVEK, TK, SQIGVL, IIK, DK, SHQHIPK, PQIDITK, VYNIDDAL, PENVVENAGL, VK, TL, K, EEDF, PF, IGDVGL, SVIR, VK, EPGAIK, APSYSISPTVQL, IYIAR, GSGK, IEIVDSNGK, AL, DTK, VEVGHL, VVPQYF, VL, AEIAGEEGIECYSIVTTTK, PL, EEL, GGR, SIWGAL, SPTL, EQVAL, NVDSDF, QK, MSK, IK, STNL, IPPVPDS, MEIDL, TPQL, PK, EL, YGGNGGSYHAWSPSQL, PML, GQGNIGASK, AL, HTNGF, AL, PR, YSDSSK, VAYVL, QGSGVAGIVL, PESEEK, VVAIK, K, GDAL, AL, PF, GVVTWWYNK, EDTEL, VVL, GDTSK, AHK, AGQF, TDF, TGSNGIF, TGF, STEF, VGR, AWDL, EESDVK, TL, VGK, QSGQGIVQL, EGNISL, PEPK, QEHR, DGMAL, NCEEAPL, DVDIK, GGGR, VVVL, NTK, NL, PL, VGEVGL, GADL, VR, DGSAMCSPGF, SCDSAL, QVTYIVR, GSGR, VQVVGVDGR, VL, ETTIQAGNL, IVPR, VVSK, IADPEGMEWF, SIITTPNPIF, THL, AGSSSVWK, AL, SPTVL, QAAF, NVDSGVEK, SK, TNDAVF, F, PPPK, MEL, DL, TPK, TAEAIF, EGDGGGYYTWSTSQMPIL, AK, TNVSAGHL, VL, HSYGF, SL, PHYADSSK, VGYVIQGTDGVVGMIL, PNF, EK, EVVF, QGDIIPVPL, GTTSWWF, NNGDSDL, IVVF, GETSK, AL, IPGQF, TYF, IL, AGTQGL, IGGF, STEL, TK, VYGL, NNDEVK, TK, SQSGVSIIK, EK, GQL, MPK, PK, MDMTK, VYNIDDAF, SNNGVK, DGGL, VTTL, TQK, DF, PF, IGDVGL, SVIR, VK, EPNAIK, APSYPVNPTVQL, IYITR, GSGK, IEIVDF, NGK, VL, DTHVK, AGYL, VVPQF, VVAEIAGEEGMDSYSIL, TTTEPL, EEL, AGK, ASVWGAL, SPTIQQVVL, NVDSEF, QHL, ISK, IK, ETTNL, IPPTN, MEIDL, TPQL, PK, EL, YGGNGGSYHAWSPSQL, PML, GQGNIGASK, AL, HTNGF, AL, PR, YSDSSK, VAYVL, QGSGVAGIVL, PESEEK, VVAIK, GDAL, AL, PF, GVVTWWYNQEDTEL, VVL, GDTSK, AHK, AGEF, TDF, TGSNGIF, TGF, STEF, VGR, AWDL, EEGDVK, TL, VGK, QSGQGIVQL, EGNISL, PEPK, QEHR, DGMAL, NCEEAPL, DVDIK, GGGR, VVVL, NTK, NL, PL, VGEVGL, GADL, VR, DGSAMCSPGF, SCDSAL, QVTYIVR, GSGR, VQVVGVDGR, VL, ETTIK, AGNL, IVPR, VVSK, IADPEGMEWF, SIITTPNPIF, THL, AGSSSVWK, AL, SPTVL, QAAF, NVDSGVEQL, SK, TNDAVF, PPPK, MGL, DL, TPK, TAEAMF, EGDGGGYYTWSTSQMPIL, AK, TNVSAGHL, VL, HPYGF, AF, PHYADSSK, VGYVIQGTDGVVGMIL, PNSGK, EVVL, QGDIIPVPL, GTISWWF, NNGDSDL, IIAF, GETSK, AL, IPGQF, TYF, IL, AGTQGL, IGGF, STEL, TK, VYGL, NNDEVK, TK, SQSGVL, IIK, EK, GQHMPK, PEIDMTK, VYNIDDAF, SNNGIK, DGGL, VTTL, TQK, DF, PF, IGDVGL, SVIR, VK, EPNAIK, APSYPVNPTVQL, IYIAR, GSGK, MEIVDF, TGK, VL, DTHVK, AGYL, VVPQF,

	VVAEIAGEEGMDSYSIL, TTTK, PL, EEL, AGK, ASVWGVL, SPTVQQVAL, NVDSEF, QNL, ISK, IK, ETTNL, IPPTN, MEIDL, TPQL, SK, YGGNGGSYHAWSPSEL, PML, R, QGNIGASK, AL, HK, NGF, AL, PR, YSDSSK, VAYVL, QGSGVAGIVL, PESEEK, VVAIK, GDAL, AL, PF, GVVTWWYNK, EDTEL, VVL, GDTSK, AHK, AGEF, TDF, TGSNGIF, TGF, STEF, VGR, AWDL, EESDVK, TL, VGK, QSGQGIVQL, EGNISL, PEPK, QEHR, DGMAL, NCEEAPL, DVDIK, GGGR, VVVL, NTK, NL, PL, VGEVGL, GADL, VR, DGSAMCSPGF, SCDSAL, QVTYIVR, GSGR, VQVVGVDGR, VL, ETTIK, AGNL, IVPR, VVSK, IADPEGMEWF, SIITTPNPIF, THL, AGSSSVWK, AL, SPTVL, QAAF, NVDSGVEQL, SK, TNDAVF, PPPK, MEIDL, TPQL, SK, VYGDNGGSYYAWSPSEL, PML, EGNIGAAK, AL, EK, NGF, AL, PR, YSDSSK, VAYVL, QGSGVAGIVL, PESEEK, VVAIK, GDAL, AL, PF, GVVTWWYNK, EDTEL, VIL, GDTSK, GHK, AGEF, TDF, TGSNGIF, TGF, STEF, VGR, AWDL, EEK, DVK, TL, VEK, QTGK, GIVK, L, DGSIK, PEPK, EEHR, GMAL, NCEEAPL, DVDIK, NGGR, VVVL, NTK, NL, PL, VGEVGL, GADL, VR, IDGNAMCSPGF, SCDSAL, QVTYIVR, GSGR, VQVVGADGR, VL, ETTIK, AGNL, IVPR, VVSK, IADPDGMAWF, SIITTPNPIF, THL, AGSISAWK, AL, SPTVL, QASF, NVDEGVEK, SK, TSDAIF, PPPN
Impossible meat	MGK, PF, TL, SL, SSL, CL, SSACF, AITSSK, NECQL, NNL, NAL, EPDHR, VESEGGL, IETWNSQHPEL, QCAGVTVSK, TL, NR, NGSHL, PSYL, PYPQMIIVVQGK, GAIGF, AF, PGCPETF, EK, PQQQSSR, GSR, SQQQL, QDSHQK, IR, HF, NEGDVL, VIPL, GVPYWTYNTGDEPVVAISPL, DTSNF, NNQL, DQNPR, VF, YL, AGNPDIEHPETMQQQQQQK, SHGGR, QGQHR, QQEEEGGSVL, SGF, SK, HF, AQSF, NTNEDTAEK, SPDDER, QIVTVEGGL, SVISPK, WQEQEDEDEDEDEEYGR, TPSYPPR, PSHGK, HEDDEDEDEEEDQPR, PDHPPQR, PSR, PEQQEPR, GR, GCQTR, NGVEENICTMK, HENIAR, PSR, ADF, YNPK, AGR, ISTL, NSL, TL, PAL, QF, GL, SAQYVVL, YR, NGIYSPDWNL, NANSVTMTR, GK, GR, VR, VVNCQGNAVF, DGEL, GQL, VVPQNPAVAEQGGEQGL, EYVVF, THHNAVSSYIK, DVF, VIPSEVL, SNSYNL, GQSQVR, QL, YQGNSGPL, VNP, MAK, VF, SL, CF, SGCCF, AF, SSR, EQPQQNECQIQK, NAL, PDNR, IESEGGL, IETWNPNNK, PF, QCAGVAL, SR, CTL, NR, NAL, PSYTNGPQEIYIQQGK, GIF, GMIYPGCPSTF, EEPQQPQQR, GQSSR, PQDR, HQK, IYNF, EGDL, IAVPTGVAWWMYNNEDTPVVAVSIIDTNSL, ENQL, DQMPR, YL, AGNQEQEF, YQQEQGGHQSQK, GK, HQQEEENEGGSIL, SGF, TL, EF, EHAF, SVDK, QIAK, NL, QGENEGEDK, GAIVTVK, GGL, SVIK, PPTDEQQQR, PQEEEEEEEDEK, PQCK, GK, DK, HCQR, PR, GSQSK, SR, NGIDETICTMR, HNIGQTSSPDIYNPQAGSVTTATSL, DF, PAL, SWL, SAEF, GSL, NAMF, VPHYNL, NANSIIYAL, NGR, AL, IQVVNCNGER, VF, DGEL, QEGR, VL, IVPQNF, VVAAR, SQSDNF, EYVSF, TNDTPMIGTL, AGANSL, NAL, PEEVIQHTF, NL, SQQAR, QIK, NNNPF, VPPQESQK, AVA, MAK, VL, SL, CF, SGCF, AL, EQAQQNECQIQK, NAL, K, PDNR, IESEGGF, IETWNPNNK, PF, QCAGVAL, SR, CTL, NR, NAL, PSYTNGPQEIYIQQGNGIF, GMIF, PGCPSTYQEPQESQQR, GR, SQR, PQDR, HQK, VHR, EGDL, IAVPTGVAWWMYNNEDTPVVAVSIIDTNSL, ENQL, DQMPR, YL, AGNQEQEF, YQQQQQGGSQSQK, GK, QQEEENEGSNIL, SGF, APEF, EAF, GVNMQIVR, NL, QGENEEEDSGAIVTVK, GGL, VTAPAMR, PQQEEDDDDEEEQPQCVETDK, GCQR, QSK, SR, NGIDETICTMR, QNIGQNSSPDIYNPQAGSITTATSL, DF, PAL, WL, SAQYGSL, NAMF, VPHYTL, NANSIIYAL, NGR, AL, VQVVNCNGER, VF, DGEL, QEGGVL, IVPQNF, AVAAK, SQSDNF, EYVSF, TNDR, PSIGNL, AGANSL, NAL, PEEVIQHTF, NL, SQQAR, QVK, NNNPF, SF, VPPQESQR, AVA, MAK, VL, SL, CF, SGCCF, AF, SF, EQPQQNECQIQR, NAL, PDNR, IESEGGF, IETWNPNNK, PF, QCAGVAL,

SR, CTL, NR, NAL, PSYTNAPQEIYIQQGSGIF, GMIF, PGCPSTF, EEPQQK, GQSSR, PQDR, HQK, IYHF, R, EGDL, IAVPTGF, AYWMYNNEDTPVVAVSL, IDTNSF, QNQL, DQMPR, YL, AGNQEQEF, QYQPQK, QQGGTQSQK, GK, QQEEENEGGSIL, SGF, APEF, L, EHAF, VVDR, QIVR, QGENEEEEK, GAIVTVK, GGL, SVISPPTEEQQQR, PEEEEK, PDCDEK, DK, HCQSQSR, NGIDETICTMR, HNIGQTSSPDIF, NPQAGSITTATSL, DF, PAL, SWL, SAQF, GSL, NAMF, VPHYNL, NANSIIYAL, NGR, AL, VQVVNCNGER, VF, DGEL, QEGQVL, IVPQNF, AVAAR, SQSDNF, EYVSF, TNDR, PSIGNL, AGANSL, NAL, PEEVIQQTF, NL, QQAR, QVK, NNNPF, SF, VPPK, ESQR, VVA, MGK, PF, TL, SL, SSL, CL, SSACF, AISSSK, NECQL, NNL, NAL, EPDHR, VESEGGL, IQTWNSQHPEL, CAGVTVSK, TL, NR, NGL, HL, PSYSPYPR, MIIIAQGK, GAL, GVAIPGCPETF, EEPQEQSNR, GSR, SQK, QQL, QDSHQK, IR, HF, NEGDVL, VIPPGVPYWTYNTGDEPVVAISL, DTSNF, NNQL, DQTPR, VF, YL, AGNPDIEYPETMQQQQQQK, SHGGR, QGQHQQEEEEEGGSVL, SGF, SK, HF, AQSF, NTNEDIAEK, QSPDDER, QIVTVEGGL, SVISPK, WQEQQDEDEDEDEDDEDEQIPSHPPR, PSHGK, EQDEDEDEDEDK, PR, PSR, PSQGK, EQDQDQDEDEDEDEDQPR, SR, EWR, SK, TQPR, PR, QEEPR, ER, GCETR, NGVEENICTL, HENIAR, PSR, ADF, YNPK, AGR, ISTL, NSL, TL, PAL, QF, QL, SAQYVVL, YK, NGIYSPHWNL, NANSVIYVTR, GQGK, VR, VVNCQGNAVF, DGEL, GQL, VVPQNF, VVAEQAGEQGF, EYIVF, THHNAVTSYL, DVF, AIPSEVL, AHSYNL, QSQVSEL, YEGNWGPL, VNPESQQGSPR, VK, VA, MMR, VR, PL, VL, GTVF, ASVCVSL, VR, EDENNPF, YL, SSNSF, QTL, ENQNGR, IR, QR, NK, SPQL, ENL, DYR, IVQF, QSK, PNTIL, PHHADADF, VL, SGR, AIL, TL, VNNDDR, DSYNL, HPGDAQR, IPAGTTYYL, VNPHDHQNL, IIK, AIPVNK, PGR, YDDF, SSTQAQQSYL, QGF, SHNIL, ETSF, HSEF, EEINR, VL, GEEEEQR, QQEGVIVEL, SK, EQIR, QL, SR, AK, SSSR, TISSEDEPF, NL, SR, NPIYSNNF, GK, EITPEK, NPQL, DL, DIF, SSVDINEGAL, PHF, NSK, AIVIL, VINEGDANIEL, VGIK, EQQQK, QK, QEEEPL, EVQR, YR, AEL, SEDDVF, VIPAAYPF, VVNATSNL, NF, AF, GINAENNQR, NF, AGEK, DNVVR, QIER, QVQEL, AF, PGSAQDVER, QR, ESYF, VDAQPQQK, EEGSK, GR, GPF, PSIL, GAL, MMR, AR, PL, GVVF, ASVSVSF, GIAYWEK, QNPSHNK, CL, SCNSEK, DSYR, NQACHAR, CNL, VEEEEECEEGQIPR, PR, PQHPER, ER, QQHGEK, EEDEGEQPR, PF, PF, PR, QPHQEEEHEQK, EEHEWHR, EEK, HGGK, GSEEEQDER, EHPR, PHQPHQK, EEEK, HEWQHK, QEK, HQGK, ESEEEEEDQDEDEEQDK, ESQESEGSESQR, EPR, HK, NK, NPF, HF, NSK, QTL, NQYGHVR, VL, QR, NK, SQQL, QNL, DYR, IL, EF, NSK, PNTL, PHHADADYL, IVIL, NGTAIL, TL, VNNDDR, DSYNL, QSGDAL, VPAGTTYYVVNPDNDENL, MITL, AIPVNK, PGR, ESF, SSTQAQQSYL, QGF, SK, NIL, EASYDTK, EEINK, VL, GR, EEGQQQGEER, QESVIVEISK, QIR, EL, SK, HAK, SSSR, TISSEDK, PF, NL, SR, DPIYSNK, GK, EITPEK, NPQL, DL, DVF, SVVDMNEGAL, PHF, NSK, AIVVL, VINEGEANIEL, VGIK, EQQQR, QQQEEQPL, EVR, YR, AEL, SEQDIF, VIPAGYPVVVNATSDL, NF, AF, GINAENNQR, NF, AGSK, DNVISQIPSQVQEL, AF, PGSAK, DIENL, IK, SQSESYF, VDAQPQQK, EEGNK, GR, GPL, SSIL, AF, MMR, AR, PL, GL, VF, ASVSVSF, GIAYWEK, ENPK, HNK, CL, QSCNSER, DSYR, NQACHAR, CNL, VEK, EECEEGEIPR, PR, PQHPER, EPQQPGEK, EEDEDEQPR, PIPF, PQPR, QEEEHEQR, EEQEWPR, EEK, GEK, GSEEEDEDEDEEQDER, QF, PF, PPHQK, EER, QEEDEDEEQQR, ESEESEDSEL, HK, NK, NPF, GSNR, ETL, NQYGR, IR, VL, QR, NQR, SPQL, QNL, DYR, IL, EF, NSK, PNTL, PNHADADYL, IVIL, NGTAIL, SL, VNNDDR, DSYR, QSGDAL, VPSGTTYYVVNPDNNENL, ITL, AIPVNK, PGR, ESF, SSTEAQQSYL, QGF, SR, NIL, EASYDTK, F, EEINK, VL, SR, EEGQQQGEQR, QESVIVEISK, EQIR, AL, SK, AK, SSSR, TISSEDK, PF,

NL, SR, DPIYSNK, GK, EITPEK, NPQL, DL, DIF, SIVDMNEGAL, PHF, NSK, AIVIL, VINEGDANIEL, VGL, EQQQEQQQEEQPL, EVR, YR, AEL, SEQDIF, VIPAGYPVVVNATSNL, NF, AIGINAENNQR, NF, AGSQDNVISQIPSQVQEL, AF, PGSAQAVEK, NQR, ESYF, VDAQPK, EEGNK, GR, GPL, SSIL, AF, MMR, AR, PL, GL, VF, ASVSVSF, GIAYWEK, ENPK, HNK, CL, QSCNSER, DSYR, NQACHAR, CNL, VEK, EECEEGEIPR, PR, PQHPER, EPQQPGEK, EEDEDEQPR, PIPF, PQPR, QEEEHEQR, EEQEWPR, EEK, GEK, GSEEEDEDEDEEQDER, QF, PF, PPHQK, EER, QEEDEDEEQQR, ESEESEDSEL, HK, NK, NPF, GSNR, ETL, NQYGR, IR, VL, QR, NQR, SPQL, QNL, DYR, IL, EF, NSK, PNTL, PNHADADYL, IVIL, NGTAIL, SL, VNNDDR, DSYR, QSGDAL, VPSGTTYYVVNPDNNENL, ITL, AIPVNK, PGR, ESF, SSTEAQQSYL, QGF, SR, NIL, EASYDTK, EEINK, VL, SR, EEGQQQGEQR, QESVIVEISK, EQIR, AL, SK, AK, SSSR, TISSEDK, PF, NL, SR, DPIYSNK, GK, EITPEK, NPQL, DL, DIF, SIVDMNEGAL, PHF, NSK, AIVIL, VINEGDANIEL, VGL, EQQQEQQQEEQPL, EVR, YR, AEL, SEQDIF, VIPAGYPVVVNATSNL, NF, AIGINAENNQR, NF, AGSQDNVISQIPSQVQEL, AF, PGSAQAVEK, NQR, ESYF, VDAQPK, EEGNK, GR, GPL, SSIL, AF, MMR, VR, PL, VL, GTVF, ASVCVSL, VR, EDENNPF, YL, SSNSF, QTL, ENQNGR, IR, QR, NK, SPQL, ENL, DYR, IVQF, QSK, PNTIL, PHHADADF, VL, SGR, AIL, TL, VNNDDR, DSYNL, HPGDAQR, IPAGTTYYL, VNPHDHQNL, IIK, AIPVNK, PGR, YDDF, SSTQAQQSYL, QGF, SHNIL, ETSF, HSEF, EEINR, VL, GEEEEQR, QQEGVIVEL, SK, EQIR, QL, SR, AK, SSSR, TISSEDEPF, NL, SR, NPIYSNNF, GK, EITPEK, NPQL, DL, DIF, SSVDINEGAL, PHF, NSK, AIVIL, VINEGDANIEL, VGIK, EQQQK, QK, QEEEPL, EVQR, YR, AEL, SEDDVF, VIPAAYPF, VVNATSNL, NF, AF, GINAENNQR, NF, AGEK, DNVVR, QIER, QVQEL, AF, PGSAQDVER, QR, ESYF, VDAQPQQK, EEGSK, GR, GPF, PSIL, GAL

Table S2. Potential bioactive peptides in BPP.

Peptide sequence	MW	PeptideRanker score[a]	CPPpred score[b]	Parent proteein	Potential bioactive peptides[d]	Biological function[d]
WGKVEADVAGH	1168.27	0.22	0.17	Myoglobin	AG, GH, GK, WG, EA, VE, GKV, WG, VA, AD, KV	ACE inhibitor, antioxidant, α-glucosidase inhibitor, DPP-IV inhibitor
NAWGKVEADVAGHGQ EVL	1880.05	0.22	0.20	Myoglobin	AW, AG, GH, GQ, GK, WG, HG, EA, EV, VE, GKV, GHG, VL, VA, AD, KV, NA, QE,	ACE inhibitor, antioxidant, neuropeptide, glucose uptake stimulation, α-glucosidase inhibitor, DPP-IV inhibitor
FTGHPETLEKFDK	1548.72	0.23	0.14	Myoglobin	GH, TG, KF, EK, HP, FDK, LEK, ET, TL, PE	ACE inhibitor, α-glucosidase inhibitor, DPP-IV inhibitor
LEFISDAIIHV	1256.46	0.22	0.06	Myoglobin	DA, AI, LEF, EF, II, HV, IH	ACE inhibitor, glucose uptake stimulation, hypolipidemic, DPP-IV inhibitor
LEFISDAIIH	1157.33	0.24	0.05	Myoglobin	DA, AI, LEF, EF, II, IH	ACE inhibitor, glucose uptake stimulation, hypolipidemic, DPP-IV inhibitor

[a]From PeptideRanker. [b]From CPPpred. [c]From ExPASy ProtParam. [d]From BIOPEP.

Table S3. Potential bioactive peptides in ByPP-pea protein.

Peptide sequence	MW	PeptideRanker score[a]	CPPpred score[b]	Parent protein	Potential bioactive peptides[d]	Biological function[d]
IESEGGLIETWNPNNKQ	1929.07	0.34	0.10	Legumin	GL, GG, EG, NK, IE, LI, SE, TW, NP, WN, ES, ET, NN, PN	ACE inhibitor, antioxidant, stimulating vasoactive substance release, glucose uptake stimulation, DPP-IV inhibitor
SEGGLIETWNPNNK	1558.67	0.54	0.12	Legumin	GL, GG, EG, NK, IE, LI, SE, TW, NP, WN, ET, NN, PN	ACE inhibitor, antioxidant, stimulating vasoactive substance release, glucose uptake stimulation, DPP-IV inhibitor
NAMFVPHYNLNANSIIY	1981.26	0.36	0.08	Legumin	IY, MF, HY, VP, LN, PH, FVP, YN, IIY, YNL, II, PHY, FVPH, HY, NA, NL, SI	ACE inhibitor, antioxidant, antiinflammation, glucose uptake stimulation, DPP-IV inhibitor
SSNNQLDQMPR	1289.39	0.43	0.12	Legumin	PR, MP, DQ, NN, NQ, QL	ACE inhibitor, DPP-IV inhibitor
DFLEDAFNVNRH	1476.57	0.44	0.10	Legumin	AF, DA, DF, FL, FN, NR, NV, RH, VN	ACE inhibitor, DPP-IV inhibitor
IESEGGLIETWNPNNK	1800.94	0.42	0.09	Legumin	GL, GG, EG, NK, IE, LI, SE, TW, NP, WN, ES, ET, NN, PN	ACE inhibitor, antioxidant, stimulating vasoactive substance release, glucose uptake stimulation, DPP-IV inhibitor
RLNIGPSSSPDIYNPE	1758.91	0.28	0.07	Legumin	GP, RL, IY, IG, LN, YN, SSS, NP, PS, PE	ACE inhibitor, antioxidant, antiamnestic, antithrombotic, stimulating vasoactive substance release, regulating the stomach mucosal membrane activity, α-glucosidase inhibitor, DPP-IV inhibitor
SLSDRFSYVAFKTNDRAGIA	2218.45	0.24	0.23	Legumin	RF, IA, AF, RA, GI, AG, SY, VAF, YVA, YV, DR, SL, KT, ND, TN, YV	ACE inhibitor, DPP-IV inhibitor
LRLNIGPSSSPDIYNPEAGR	2156.38	0.23	0.11	Legumin	GP, RL, IY, IG, AG, GR, EA, LN, YN, LR, SSS, NP, PS, PE	ACE inhibitor, antioxidant, antiamnestic, antithrombotic, stimulating vasoactive substance release, regulating the stomach mucosal membrane activity, DPP-IV inhibitor
NLNANSIIYALKGR	1546.79	0.25	0.18	Legumin	IY, YA, GR, KG, LN, IIY, II, LK, AL, LN, NA, NL, SI	ACE inhibitor, antioxidant, glucose uptake stimulation, DPP-IV inhibitor
SEGGLIETWNPNNKQ	1686.80	0.44	0.12	Legumin	GL, GG, EG, NK, IE, LI, SE, TW, NP, WN, ES, ET, NN, PN	ACE inhibitor, stimulating vasoactive substance release, glucose uptake stimulation, DPP-IV inhibitor

DFLEDAFNVNR	1339.43	0.41	0.11	Legumin	AF, DA, DF, FL, FN, NR, NV, VN	ACE inhibitor, DPP-IV inhibitor
VKGGLSIISPPEKQAR	1679.98	0.28	0.18	Legumin	VK, GL, KG, GG, AR, PP, EK, II, SP, QA, SI, PE	ACE inhibitor, glucose uptake stimulation, α-glucosidase inhibitor, DPP-IV inhibitor
LRLNIGPSSSPDI	1368.55	0.21	0.09	Legumin	GP, RL, IG, LN, LR, SSS, SP, LN, PS	ACE inhibitor, antiamnestic, antithrombotic, stimulating vasoactive substance release, regulating the stomach mucosal membrane activity, DPP-IV inhibitor
NALEPDNRIESEGGL	1613.70	0.26	0.10	Legumin	GL, GG, EG, IE, ALEP, SE, EP, AL, DN, ES, NA, NR, RI	ACE inhibitor, stimulating vasoactive substance release, DPP-IV inhibitor
LNIGPSSSPDIYNPEAGR	1887.04	0.24	0.07	Legumin	GP, IY, IG, AG, GR, EA, LN, SSS, NP, LN, PS, YN, EA, PE	ACE inhibitor, antioxidant, antiamnestic, antithrombotic, stimulating vasoactive substance release, regulating the stomach mucosal membrane activity, α-glucosidase inhibitor, DPP-IV inhibitor
SLSDRFSYVAFK	1419.60	0.56	0.12	Legumin	RF, AF, SY, VAF, YVA, YV, DR, SL, VA	ACE inhibitor, DPP-IV inhibitor
LAGTSSVINNLPLDVVAATF	2002.30	0.20	0.18	Legumin	VAA, PL, LA, AA, AG, GT, TF, LPL, LP, VV, AT, IN, NL, NN, SV, TS, VI	ACE inhibitor, antioxidant, hypotensive, DPP-IV inhibitor
WLKLSAEHGSLH	1377.57	0.32	0.17	Legumin	LKL, GS, HG, KL, WL, SL, LH, LK, SAEHGSLH, AE, EH	ACE inhibitor, antioxidant, DPP-IV inhibitor
LAGNHEQEFLR	1313.44	0.40	0.16	Legumin	LA, AG, LR, EF, FL, HE, NH, QE	ACE inhibitor, hypolipidemic, DPP-IV inhibitor
WLKLSAEHGSLHK	1505.74	0.36	0.24	Legumin	LKL, GS, HG, KL, HK, WL, SL, LH, LHK, LK, SAEHGSLH, AE, EH	ACE inhibitor, antioxidant, DPP-IV inhibitor
LNALEPDNR	1041.13	0.22	0.23	Legumin	LN, ALEP, EP, AL, DN, LN, NA, NR	ACE inhibitor, DPP-IV inhibitor
TVTSLDLPVLRW	1399.65	0.26	0.52	Legumin	LRW, DLP, RW, LR, LP, VL, SL, PV, TS, TV, VL, VT	ACE inhibitor, antioxidant, glucose uptake stimulation, DPP-IV inhibitor
VKGGLSIISPPEKQ	1452.71	0.21	0.11	Legumin	VK, GL, KG, GG, PP, EK, II, SP, SI, VK, PE	ACE inhibitor, glucose uptake stimulation, α-glucosidase inhibitor, DPP-IV inhibitor

RLNIGPSSSPDIYNPEAG	1887.04	0.22	0.07	Legumin	GP, IG, AG, RL IY, EA, LN, YN, SSS, SP, NP, PS, PE	ACE inhibitor, antioxidant, antiamnestic, antithrombotic, stimulating vasoactive substance release, regulating the stomach mucosal membrane activity, α-glucosidase inhibitor, DPP-IV inhibitor
IESEGGLIETWNPNNKQFR	2232.44	0.58	0.11	Legumin	FR, GL, GG, EG, NK, IE, LI, SE, TW, NP, WN, ES, ET, NN, PN	ACE inhibitor, antioxidant, stimulating vasoactive substance release, glucose uptake stimulation, DPP-IV inhibitor
KGGLSIISPPE	1097.28	0.23	0.07	Legumin	GL, KG, GG, PP, II, SP, SI, PE	ACE inhibitor, glucose uptake stimulation, α-glucosidase inhibitor, DPP-IV inhibitor
KGGLSIISPPEK	1225.45	0.29	0.09	Legumin	GL, KG, GG, PP, EK, II, SP, SI, PE	ACE inhibitor, glucose uptake stimulation, α-glucosidase inhibitor, DPP-IV inhibitor
LNIGPSSSPDIYNPEAGRIK	2128.37	0.24	0.18	Legumin	GP, IY, IG, AG, GR, EA, LN, YN, SSS, NP, LN, PS, PE	ACE inhibitor, antioxidant, antiamnestic, antithrombotic, stimulating vasoactive substance release, regulating the stomach mucosal membrane activity, α-glucosidase inhibitor, DPP-IV inhibitor
GGLSIISPPEK	1097.28	0.36	0.07	Legumin	GL, GG, PP, EK, II, SP, SI, PE	ACE inhibitor, glucose uptake stimulation, α-glucosidase inhibitor, DPP-IV inhibitor
RDFLEDAFNVN	1339.43	0.22	0.11	Legumin	AF, DA, DF, FL, FN, NV, VN	ACE inhibitor, DPP-IV inhibitor
FREGDIIAVPT	1217.39	0.20	0.09	Legumin	AVP, IA, VP, FR, GD, EG, PT, AV, II, VP, PT	ACE inhibitor, glucose uptake stimulation, DPP-IV inhibitor
IETWNPNNK	1115.21	0.28	0.16	Legumin	NK, IE, TW, NP, WN, ET, NN, PN	ACE inhibitor, antioxidant, DPP-IV inhibitor
IESEGGLIETWNPNN	1672.77	0.32	0.07	Legumin	GL, GG, EG, IE, LI, SE, TW, NP, WN, ES, ET, NN, PN	ACE inhibitor, antioxidant, stimulating vasoactive substance release, glucose uptake stimulation, DPP-IV inhibitor
NIGPSSSPDIYNPEAGR	1773.88	0.26	0.06	Legumin	GP, IY, IG, AG, GR, EA, YN, SSS, SP, NP, PS, PE	ACE inhibitor, antioxidant, antiamnestic, antithrombotic, stimulating vasoactive substance release, regulating the stomach mucosal membrane activity, α-glucosidase inhibitor, DPP-IV inhibitor
VKGGLSIISPPEKQA	1523.79	0.20	0.12	Legumin	VK, GL, KG, GG, PP, EK, II, SP, QA, SI, VK, PE	ACE inhibitor, glucose uptake stimulation, α-glucosidase inhibitor, DPP-IV inhibitor

LRLNIGPSSSPDIYNPE	1872.06	0.27	0.08	Legumin	GP, RL, IY, IG, LN, YN, LR, SSS, NP, PS, PE	ACE inhibitor, antioxidant, antiamnestic, antithrombotic, stimulating vasoactive substance release, regulating the stomach mucosal membrane activity, *α*-glucosidase inhibitor, DPP-IV inhibitor
SLSDRFSYVAF	1291.43	0.64	0.08	Legumin	RF, AF, SY, VAF, YVA, YV, DR, SL, VA	ACE inhibitor, DPP-IV inhibitor
LNALEPDNRIESEGGL	1726.86	0.26	0.11	Legumin	GL, GG, EG, IE, LN, ALEP, SE, EP, AL, GL, DN, ES, NA, NR, RI	ACE inhibitor, stimulating vasoactive substance release, DPP-IV inhibitor
FREGDIIAVPTGIVF	1633.91	0.45	0.08	Legumin	AVP, VF, IA, VP, FR, GI, GD, TG, EG, PT, IVF, AV, IV, II	ACE inhibitor, glucose uptake stimulation, DPP-IV inhibitor
KRDFLEDAFNVN	1467.60	0.26	0.15	Legumin	AF, KR, DA, DF, FL, FN, NV, VN	ACE inhibitor, DPP-IV inhibitor
YSNAPQEIFIQQGNG	1665.78	0.29	0.06	Legumin	AP, IF, QG, NG, EI, PQ, AP, EI, IQ, NA, QE, QQ, YS	ACE inhibitor, DPP-IV inhibitor
FREGDIIAVPTGIV	1486.73	0.28	0.09	Legumin	AVP, IA, VP, FR, GI, GD, TG, EG, PT, IVF, AV, IV, II	ACE inhibitor, glucose uptake stimulation, regulating the stomach mucosal membrane activity, DPP-IV inhibitor
LRLNIGPSSSPDIYNPEAG	2000.20	0.22	0.08	Legumin	GP, RL, IY, IG, AG, EA, LN, YN, LR, SSS, SP, NP, PS, PE	ACE inhibitor, antioxidant, antiamnestic, antithrombotic, stimulating vasoactive substance release, regulating the stomach mucosal membrane activity, *α*-glucosidase inhibitor, DPP-IV inhibitor
SIISPPEKQ	998.14	0.22	0.06	Legumin	PP, EK, II, SP, SI	ACE inhibitor, glucose uptake stimulation, *α*-glucosidase inhibitor, DPP-IV inhibitor
SNNQLDQMPR	1202.31	0.44	0.15	Legumin	PR, MP, DQ, NN, NQ, QL	ACE inhibitor, DPP-IV inhibitor
SVINNLPLD	984.12	0.34	0.10	Legumin	PL, LPL, LP, IN, NL, NN, SV, VI	ACE inhibitor, antioxidant, DPP-IV inhibitor
YSNAPQEIFIQQGNGY	1828.96	0.28	0.06	Legumin	GY, AP, IF, QG, NG, EI, PQ, IQ, NA, NG, QE, QQ, YS	ACE inhibitor, DPP-IV inhibitor
LNIGPSSSPDIYNPEAG	1730.85	0.22	0.06	Legumin	GP, IY, IG, AG, EA, LN, YN, SSS, SP, NP, PS, PE	ACE inhibitor, antioxidant, antiamnestic, antithrombotic, stimulating vasoactive substance release, regulating the stomach mucosal membrane activity, *α*-glucosidase inhibitor, DPP-IV inhibitor

NLNANSIIYALKG	1390.60	0.27	0.12	Legumin	IY, YA, KG, LN, IIY, II, AL, NA, NL, SI	ACE inhibitor, antioxidant, glucose uptake stimulation, DPP-IV inhibitor
NNLPLDVVAATFNLQR	1785.03	0.40	0.34	Legumin	VAA, PL, AA, LQ, TF, LPL, LP, NLQ, VA, VV, AT, FN, NL, NN	ACE inhibitor, antioxidant, antiinflammation, hypotensive, DPP-IV inhibitor
FKRDFLEDAF	1287.44	0.52	0.12	Legumin	AF, KR, DA, DF, FKR, FL	ACE inhibitor, DPP-IV inhibitor
PSSSPDIYNPEAGRI	1602.72	0.51	0.06	Legumin	IY, AG, GR, EA, YN, SSS, SP, NP, PS, RI, PE	ACE inhibitor, antioxidant, stimulating vasoactive substance release, *α*-glucosidase inhibitor, DPP-IV inhibitor
VKGGLSIISPPEK	1324.58	0.21	0.11	Legumin	VK, GL, KG, GG, PP, EK, II, SP, SI, PE	ACE inhibitor, glucose uptake stimulation, *α*-glucosidase inhibitor, DPP-IV inhibitor
IKTVTSLDLPVLRW	1640.99	0.23	0.46	Legumin	LRW, DLP, RW, LR, LP, VL, SL, PV, TS, TV, VL, VT	ACE inhibitor, antioxidant, glucose uptake stimulation, DPP-IV inhibitor
SEGGLIETWNPNN	1430.49	0.43	0.09	Legumin	GL, GG, EG, IE, LI, SE, TW, NP, WN, ET, NN, PN	ACE inhibitor, antioxidant, stimulating vasoactive substance release, glucose uptake stimulation, DPP-IV inhibitor
IRSSNNQLDQMPR	1558.73	0.22	0.16	Legumin	IR, PR, MP, DQ, NN, NQ, QL	ACE inhibitor, antioxidant, DPP-IV inhibitor
FDGELEAGRA	1064.12	0.37	0.12	Legumin	RA, AG, GR, GE, EA, DG, EL	ACE inhibitor, antioxidant, *α*-glucosidase inhibitor, DPP-IV inhibitor
RLNIGPSSSPDI	1255.39	0.21	0.07	Legumin	GP, RL, IG, LN, SSS, SP, PS	ACE inhibitor, antiamnestic, antithrombotic, stimulating vasoactive substance release, regulating the stomach mucosal membrane activity, DPP-IV inhibitor
SSNNQLDQM	1036.08	0.39	0.07	Legumin	DQ, NN, NQ, QL	DPP-IV inhibitor
GNTVFDGELEAGR	1364.43	0.21	0.13	Legumin	VF, AG, GR, GE, EA, DG, EL, NT, TV	ACE inhibitor, antioxidant, *α*-glucosidase inhibitor, DPP-IV inhibitor
SSNNQLDQMP	1133.20	0.41	0.08	Legumin	MP, DQ, NN, NQ, QL	DPP-IV inhibitor
DFLEDAFNVNRHIVD	1803.95	0.22	0.09	Legumin	AF, DA, DF, IV, RHI, FL, FN, HI, NR, NV, RH, VD, VN	ACE inhibitor, antioxidant, glucose uptake stimulation, DPP-IV inhibitor
GGLSIISPPE	969.10	0.31	0.05	Legumin	GL, GG, PP, II, SP, SI, PE	ACE inhibitor, glucose uptake stimulation, *α*-glucosidase inhibitor, DPP-IV inhibitor
LAGTSSVINNLPL	1298.50	0.24	0.14	Legumin	PL, LA, AG, GT, LPL, LP, IN, NL, NN, SV, TS, VI	ACE inhibitor, antioxidant, DPP-IV inhibitor

GGLSIISPPEKQ	1225.41	0.32	0.07	Legumin	GL, GG, PP, EK, II, SP, SI, PE	ACE inhibitor, glucose uptake stimulation, α-glucosidase inhibitor, DPP-IV inhibitor
SLSDRFS	810.86	0.47	0.08	Legumin	RF, DR, SL	ACE inhibitor, DPP-IV inhibitor
VINNLPLD	897.04	0.25	0.13	Legumin	PL, LPL, LP, IN, NL, NN, VI	ACE inhibitor, antioxidant, DPP-IV inhibitor
SIISPPEK	870.01	0.29	0.06	Legumin	PP, EK, II, SP, SI, PE	ACE inhibitor, glucose uptake stimulation, α-glucosidase inhibitor, DPP-IV inhibitor
NQLDQMPR	1001.13	0.47	0.24	Legumin/ Glycinin	PR, MP, DQ, NQ, QL	ACE inhibitor, DPP-IV inhibitor
NIGPSSSPDIYNPEAG	1617.69	0.24	0.05	Legumin	GP, IY, IG, AG, EA, LN, YN, SSS, SP, NP, PS, PE	ACE inhibitor, antioxidant, antiamnestic, antithrombotic, stimulating vasoactive substance release, regulating the stomach mucosal membrane activity, α-glucosidase inhibitor, DPP-IV inhibitor
GGLSIISPPEKQAR	1452.67	0.42	0.12	Legumin	GL, GG, AR, PP, EK, II, SP, QA, SI, PE	ACE inhibitor, glucose uptake stimulation, α-glucosidase inhibitor, DPP-IV inhibitor
NAMFVPHYNLN	1319.50	0.47	0.10	Legumin/ Glycinin	MF, HY, VP, LN, PH, FVP, YN, YNL, NA, NL	ACE inhibitor, antioxidant, antiinflammation, DPP-IV inhibitor
LNIGPSSSPDIYNPE	1602.72	0.27	0.05	Legumin	GP, IY, IG, LN, YN, SSS, SP, NP, PS, PE	ACE inhibitor, antioxidant, antiamnestic, antithrombotic, stimulating vasoactive substance release, regulating the stomach mucosal membrane activity, α-glucosidase inhibitor, DPP-IV inhibitor
NAMFVPH	814.96	0.62	0.08	Legumin/ Glycinin	MF, VP, PH, FVP, FVPH, NA	ACE inhibitor, antioxidant, DPP-IV inhibitor
NIGPSSSPDIYNPE	1489.56	0.30	0.05	Legumin	GP, IY, IG, YN, SSS, SP, NP, PS, PE	ACE inhibitor, antioxidant, antiamnestic, antithrombotic, stimulating vasoactive substance release, regulating the stomach mucosal membrane activity, α-glucosidase inhibitor, DPP-IV inhibitor
NAMFVPHYNLNANSII	1818.08	0.33	0.08	Legumin	MF, HY, VP, LN, PH, FVP, YN, YNL, PHY, FVPH, NA, NL, SI	ACE inhibitor, antioxidant, antiinflammation, glucose uptake stimulation, DPP-IV inhibitor
SLSDRFSYVAFKTND	1749.90	0.22	0.11	Legumin	RF, AF, SY, VAF, YVA, YV, DR, SL, KT, ND, TN	ACE inhibitor, DPP-IV inhibitor

NAMFVPHYNLNAN	1504.68	0.35	0.11	Legumin/ Glycinin	MF, HY, VP, LN, PH, FVP, YN, YNL, PHY, FVPH, NA, NL	ACE inhibitor, antioxidant, antiinflammation, DPP-IV inhibitor
DFLEDAFNVNRHIVDR	1960.14	0.26	0.13	Legumin	AF, DA, DF, DR, IV, RHI, FL, FN, HI, NR, NV, RH, VD, VN	ACE inhibitor, antioxidant, glucose uptake stimulation, DPP-IV inhibitor
IISPPE	654.76	0.29	0.05	Legumin	PP, II, SP, PE	ACE inhibitor, glucose uptake stimulation, *α*-glucosidase inhibitor, DPP-IV inhibitor
DFLEDAFNVNRHIVDR LQG	2258.48	0.21	0.14	Legumin	RL, AF, DA, QG, LQ, DF, DR, IV, RHI, FL, FN, HI, NR, NV, RH, VD, VN	ACE inhibitor, antioxidant, glucose uptake stimulation, DPP-IV inhibitor
SIISPPE	741.84	0.33	0.04	Legumin	PP, II, SP, SI, PE	ACE inhibitor, glucose uptake stimulation, *α*-glucosidase inhibitor, DPP-IV inhibitor
ALEPDNRIESEGGL	1499.60	0.31	0.10	Legumin	GL, GG, EG, IE, ALEP, SE, EP, AL, DN, ES, NR, RI	ACE inhibitor, stimulating vasoactive substance release, DPP-IV inhibitor
NNLPLDVVAATFNLQR NEAR	2255.52	0.24	0.41	Legumin	VAA, PL, AA, EA, AR, LQ, TF, LPL, LP, NLQ, VA, VV, AT, FN, NL, NN	ACE inhibitor, antioxidant, antiinflammation, hypotensive, *α*-glucosidase inhibitor, DPP-IV inhibitor
AGTSSVINNLPL	1185.34	0.30	0.12	Legumin	PL, AG, GT, LPL, LP, IN, NL, NN, SV, TS, VI	ACE inhibitor, antioxidant, DPP-IV inhibitor
DFLEDAFNVN	1183.24	0.28	0.07	Legumin	AF, DA, DF, FL, FN, NV, VN	ACE inhibitor, DPP-IV inhibitor
INNLPL	682.82	0.56	0.16	Legumin	PL, LPL, LP, IN, NL, NN	ACE inhibitor, antioxidant, DPP-IV inhibitor
KFLVPA	673.85	0.42	0.32	Legumin	VP, KF, LV, PA, FL	ACE inhibitor, glucose uptake stimulation, DPP-IV inhibitor
PQEIFIQQGNGYFGMV	1828.07	0.76	0.06	Legumin	GY, IF, GM, FG, QG, NG, EI, PQ, IQ, MV, NG, QE, QQ, YF	ACE inhibitor, DPP-IV inhibitor
LNIGPSSSPDIYNPEA	1673.80	0.25	0.06	Legumin	GP, IY, IG, EA, LN, YN, SSS, SP, NP, PS, PE	ACE inhibitor, antioxidant, antiamnestic, antithrombotic, stimulating vasoactive substance release, regulating the stomach mucosal membrane activity, *α*-glucosidase inhibitor, DPP-IV inhibitor
GGLSIISPPEKQA	1296.49	0.31	0.08	Legumin	GL, GG, PP, EK, II, QA, SI, PE	ACE inhibitor, glucose uptake stimulation, *α*-glucosidase inhibitor, DPP-IV inhibitor
RLAGTSSVINNLPL	1454.69	0.25	0.22	Legumin	RL, PL, LA, AG, GT, LPL, LP, IN, NL, NN, TS, VI	ACE inhibitor, antioxidant, DPP-IV inhibitor

SVINNLPL	869.03	0.51	0.13	Legumin	PL, LPL, LP, IN, NL, NN, SV, VI	ACE inhibitor, antioxidant, DPP-IV inhibitor
NANSIIYALKGR	1319.53	0.31	0.16	Legumin	IY, YA, GR, KG, IIY, II, LK, AL, NA, SI	ACE inhibitor, antioxidant, glucose uptake stimulation, DPP-IV inhibitor
KNAMFVPHYNLNA	1518.75	0.39	0.15	Legumin/ Glycinin	MF, HY, VP, LN, PH, FVP, YN, YNL, PHY, FVPH, NA, NL	ACE inhibitor, antioxidant, antiinflammation, DPP-IV inhibitor
SNAPQEIFIQQGNGYF	1812.96	0.56	0.06	Legumin	GY, AP, IF, QG, NG, EI, PQ, EI, IQ, NA, QE, QQ, YF	ACE inhibitor, DPP-IV inhibitor
QEQENEGNNIFSGF	1612.63	0.46	0.05	Legumin	GF, IF, SG, EG, NE, NN, QE	ACE inhibitor, DPP-IV inhibitor
AGTSSVINNLPLDVVAATF	1889.14	0.23	0.16	Legumin	VAA, PL, AA, AG, GT, TF, LPL, LP, AT, IN, NL, NN, SV, TS, VI	ACE inhibitor, antioxidant, hypotensive, DPP-IV inhibitor
IESEGGLIETWNP	1444.56	0.28	0.07	Legumin	GL, GG, EG, IE, LI, SE, TW, NP, WN, ES, ET, LI	ACE inhibitor, antioxidant, stimulating vasoactive substance release, glucose uptake stimulation, DPP-IV inhibitor
FVPHYNLNAN	1188.31	0.45	0.10	Legumin/ Glycinin	HY, VP, LN, PH, FVP, YN, YNL, PHY, FVPH, NA, NL	ACE inhibitor, antioxidant, antiinflammation, DPP-IV inhibitor
DALEPDNRIESEGGL	1614.69	0.29	0.09	Legumin	GL, DA, GG, EG, IE, ALEP, SE, EP, AL, DN, ES, NR, RI	ACE inhibitor, stimulating vasoactive substance release, DPP-IV inhibitor
LDALEPDNR	1042.11	0.25	0.18	Legumin	DA, ALEP, EP, AL, DN, NR	ACE inhibitor, DPP-IV inhibitor
LDALEPDNRIESEGGL	1727.85	0.27	0.10	Legumin	GL, DA, GG, EG, IE, ALEP, SE, EP, AL, DN, ES, NR, RI	ACE inhibitor, stimulating vasoactive substance release, DPP-IV inhibitor
NQQLQDLDIFVNSVDIK	1989.21	0.31	0.11	Vicilin/Provicilin	IF, LQ, NQ, QD, QL, QQ, SV, VD, VN	ACE inhibitor, DPP-IV inhibitor
IKEGSLLLPNYNSR	1603.84	0.31	0.15	Vicilin	LLP, GS, EG, NY, KE, YN, LP, LLL, LL, SL, PN	ACE inhibitor, stimulating vasoactive substance release, glucose uptake stimulation, DPP-IV inhibitor
GFGINAENNER	1220.26	0.21	0.09	Vicilin	GF, GI, FG, ER, AE, IN, NA, NE, NN	ACE inhibitor, DPP-IV inhibitor
SGTQNQPSLLSGFSK	1550.69	0.40	0.11	Vicilin	GF, GT, SG, TQ, QP, LL, SL, NQ, PS, QN, SK	ACE inhibitor, glucose uptake stimulation, DPP-IV inhibitor

AKLSPGDVFVIPAGHPV	1704.00	0.48	0.13	Vicilin/Provicilin	PG, VF, IPA, LSP, IP, AG, GH, GD, KL, HP, PA, IP, SP, PV, VI	ACE inhibitor, antiamnestic, antithrombotic, regulating the stomach mucosal membrane activity, DPP-IV inhibitor
IKEGSLLLPNYNS	1447.65	0.21	0.10	Vicilin	LLP, GS, EG, NY, KE, YN, LP, LLL, LL, SL, PN	ACE inhibitor, stimulating vasoactive substance release, glucose uptake stimulation, DPP-IV inhibitor
FANAQPLQRE	1173.29	0.36	0.23	Vicilin	PL, LQ, QP, FA, NA, FA	ACE inhibitor, DPP-IV inhibitor
RSDQENPFIFK	1380.52	0.52	0.09	Vicilin/Provicilin	IF, NP, DQ, PF, QE, PF	ACE inhibitor, DPP-IV inhibitor
NKPGQLQSFLLSGTQNQPSL	2157.41	0.55	0.20	Vicilin	PG, GQ, GT, SG, SF, NK, KP, LQ, TQ, QP, LL, SL, NQ, PS, QL, QN, QS	ACE inhibitor, antioxidant, antiamnestic, neuropeptide, antithrombotic, glucose uptake stimulation, regulating the stomach mucosal membrane activity, DPP-IV inhibitor
GFGINAENNERN	1334.37	0.23	0.09	Vicilin	GF, GI, FG, ER, AE, IN, NA, NE, NN, RN	ACE inhibitor, DPP-IV inhibitor
SRNPIYSNKFG	1282.42	0.59	0.09	Vicilin/Provicilin	IY, FG, KF, NK, NP, PI, RN, YS	ACE inhibitor, antioxidant, DPP-IV inhibitor
LLSGTQNQPSLLSGFSK	1777.01	0.28	0.17	Vicilin	GF, GT, SG, TQ, QP, LL, SL, NQ, PS, QN, SK	ACE inhibitor, glucose uptake stimulation, DPP-IV inhibitor
EGKGDFELVGQ	1178.26	0.27	0.09	Vicilin/Provicilin	VG, KG, GQ, GK, GD, EG, DF, LV, EL	ACE inhibitor, antioxidant, neuropeptide, glucose uptake stimulation, DPP-IV inhibitor
FFEITPEKNQQL	1493.68	0.23	0.09	Vicilin/Provicilin	EI, EK, TP, FF, QL, QQ, PE	ACE inhibitor, α-glucosidase inhibitor, DPP-IV inhibitor
LAFPGSSHEVDR	1314.42	0.31	0.10	Vicilin/Provicilin	PG, FP, AFP, AF, LA, GS, EV, DR, LAF, HE, SH, VD	ACE inhibitor, antioxidant, antiamnestic, antithrombotic, regulating the stomach mucosal membrane activity, DPP-IV inhibitor
NQQLQDLDIFVN	1446.58	0.34	0.11	Vicilin/Provicilin	IF, LQ, NQ, QD, QL, QQ, VN	ACE inhibitor, DPP-IV inhibitor
RSDQENPFIF	1252.35	0.65	0.07	Vicilin/Provicilin	IF, NP, DQ, PF, QE	ACE inhibitor, DPP-IV inhibitor
IGFGINAENNERN	1447.53	0.20	0.08	Vicilin	GF, IG, GI, FG, ER, AE, IN, NA, NN, NE, RN	ACE inhibitor, DPP-IV inhibitor

NQQLQDLDIFVNSV	1632.79	0.25	0.10	Vicilin/Provicilin	IF, LQ, NQ, QD, QL, QQ, SV, VN	ACE inhibitor, DPP-IV inhibitor
EGSLLLPNYNSR	1362.51	0.33	0.14	Vicilin	LLP, GS, EG, NY, YN, LP, LLL, LL, SL, PN	ACE inhibitor, stimulating vasoactive substance release, glucose uptake stimulation, DPP-IV inhibitor
LAFPGSSHEVDRL	1427.58	0.49	0.12	Vicilin	PG, RL, FP, AFP, AF, LA, GS, EV, DR, LAF, HE, SH, VD	ACE inhibitor, antioxidant, antiamnestic, antithrombotic, regulating the stomach mucosal membrane activity, DPP-IV inhibitor
IPVNKPGQLQ	1093.29	0.21	0.21	Vicilin/Provicilin	PG, IP, GQ, NK, KP, LQ, KP, IP, PV, QL, VN	ACE inhibitor, antioxidant, antiamnestic, neuropeptide, antithrombotic, regulating the stomach mucosal membrane activity, DPP-IV inhibitor
GDAIKLPAGTI	1055.24	0.52	0.13	Vicilin	AG, DA, GT, AI, GD, KL, KLP, LP, IKL, PA, TI	ACE inhibitor, antioxidant, DPP-IV inhibitor
LSGTQNQPSLLSGFSK	1663.85	0.31	0.13	Vicilin	GF, GT, SG, TQ, QP, LL, SL, NQ, PS, QN, SK	ACE inhibitor, glucose uptake stimulation, DPP-IV inhibitor
SKIFENLQ	978.11	0.22	0.09	Vicilin/Provicilin	IF, LQ, NLQ, KI, NL, SK	ACE inhibitor, antiinflammation, DPP-IV inhibitor
DLAIPVNKPGQLQ	1392.62	0.29	0.20	Vicilin/Provicilin	PG, AIP, IP, LA, GQ, AI, NK, KP, LQ, PV, QL, VN	ACE inhibitor, antioxidant, antiamnestic, neuropeptide, antithrombotic, regulating the stomach mucosal membrane activity, DPP-IV inhibitor
EGSLLLPNYNS	1206.32	0.22	0.09	Vicilin	LLP, GS, EG, NY, YN, LP, LLL, LL, SL, PN	ACE inhibitor, stimulating vasoactive substance release, glucose uptake stimulation, DPP-IV inhibitor
SKIFENLQNYR	1411.58	0.29	0.12	Vicilin/Provicilin	IF, NY, LQ, IFENLQN, YR, NLQ, KI, NL, QN, SK	ACE inhibitor, antiinflammation, neuropeptide, DPP-IV inhibitor
FVNSVDIKEGSLLLPNYNSR	2265.55	0.47	0.12	Vicilin	LLP, GS, EG, NY, KE, YN, LP, LLL, LL, SL, PN, SV, VD, VN	ACE inhibitor, stimulating vasoactive substance release, glucose uptake stimulation, DPP-IV inhibitor
NLERGDAIKLPAGTI	1567.81	0.20	0.20	Vicilin	AG, DA, GT, AI, GD, KL, RG, KLP, ER, LP, RGD, IKL, PA, NL, TI	ACE inhibitor, antioxidant, antithrombotic, DPP-IV inhibitor
EGSLLLPNYN	1119.24	0.36	0.11	Vicilin	LLP, GS, EG, NY, YN, LP, LLL, LL, SL	ACE inhibitor, stimulating vasoactive substance release, glucose uptake stimulation, DPP-IV inhibitor

LAIPVNKPGQ	1036.24	0.26	0.22	Vicilin/Provicilin	PG, AIP, IP, LA, GQ, AI, NK, KP, LQ, PV, QL, VN	ACE inhibitor, antioxidant, antiamnestic, neuropeptide, antithrombotic, regulating the stomach mucosal membrane activity, DPP-IV inhibitor
VLDLAIPVNKPGQL	1476.78	0.41	0.25	Vicilin/Provicilin	PG, AIP, IP, LA, GQ, AI, NK, KP, LQ, PV, QL, VN	ACE inhibitor, antioxidant, antiamnestic, neuropeptide, antithrombotic, glucose uptake stimulation, regulating the stomach mucosal membrane activity, DPP-IV inhibitor
FVIPAGHPVAINA	1305.54	0.39	0.09	Vicilin/Provicilin	IPA, IP, AG, GH, AI, HP, VA, PA, GH, IN, NA, PV, VI	ACE inhibitor, DPP-IV inhibitor
LAIPVNKPGQLQ	1277.53	0.25	0.26	Vicilin/Provicilin	PG, AIP, IP, LA, GQ, AI, NK, KP, LQ, PV, QL, VN	ACE inhibitor, antioxidant, antiamnestic, neuropeptide, antithrombotic, regulating the stomach mucosal membrane activity, DPP-IV inhibitor
YENENGHIRL	1244.33	0.22	0.09	Vicilin/Provicilin	RL, IR, HIRL, HIR, GH, NG, YE, NEN, HI, NE	ACE inhibitor, antioxidant, neuropeptide, DPP-IV inhibitor
FEITPEKNQQLQDLDIF	2078.31	0.26	0.09	Vicilin/Provicilin	IF, EI, LQ, EK, TP, NQ, QD, QL, QQ, PE	ACE inhibitor, α-glucosidase inhibitor, DPP-IV inhibitor
AIPVNKPGQLQ	1164.37	0.29	0.23	Vicilin/Provicilin	PG, AIP, IP, GQ, AI, NK, KP, LQ, PV, QL, VN	ACE inhibitor, antioxidant, antiamnestic, neuropeptide, antithrombotic, regulating the stomach mucosal membrane activity, DPP-IV inhibitor
LANRDDNEDLRV	1429.51	0.21	0.24	Vicilin	LA, LR, LAN, DN, NE, NR	ACE inhibitor, antioxidant, DPP-IV inhibitor
LAFPGSSH	814.90	0.36	0.06	Vicilin/Provicilin	PG, FP, AFP, AF, LA, GS, LAF, SH	ACE inhibitor, antioxidant, antiamnestic, antithrombotic, regulating the stomach mucosal membrane activity, DPP-IV inhibitor
SVSSESGPFNLR	1279.37	0.38	0.08	Vicilin/Provicilin	GP, SG, SGP, LR, NLR, SE, ES, FN, NL, PF, SV, GRF	ACE inhibitor, antiamnestic, antithrombotic, stimulating vasoactive substance release, regulating the stomach mucosal membrane activity, DPP-IV inhibitor
TLFLPQ	717.86	0.41	0.23	Vicilin/Provicilin	LF, PQ, LP, FL, TL, LPQ	ACE inhibitor, DPP-IV inhibitor

RSDQENPF	992.01	0.41	0.08	Vicilin/Provicilin	NP, DQ, PF, QE	DPP-IV inhibitor
FVIPAGHPV	936.12	0.37	0.08	Vicilin/Provicilin	IPA, IP, AG, GH, HP, PA, PV, VI	ACE inhibitor, DPP-IV inhibitor
FFEITPEKNQQLQDL	1850.06	0.23	0.11	Vicilin/Provicilin	EI, LQ, EK, TP, FF, NQ, QD, QL, QQ, PE	ACE inhibitor, α-glucosidase inhibitor, DPP-IV inhibitor
SRSDQENPF	1079.09	0.44	0.07	Vicilin/Provicilin	NP, DQ, PF, QE	DPP-IV inhibitor
IFENLQNYRL	1309.49	0.51	0.13	Vicilin/Provicilin	RL, IF, NY, LQ, IFENLQN, YR, NLQ, NL, QN	ACE inhibitor, antiinflammation, neuropeptide, DPP-IV inhibitor
VDIKEGSLLLPNYNS	1661.87	0.23	0.10	Vicilin	LLP, GS, EG, NY, KE, YN, LP, LLL, LL, SL, PN, VD	ACE inhibitor, stimulating vasoactive substance release, glucose uptake stimulation, DPP-IV inhibitor
PQYTDADFIL	1182.30	0.55	0.07	Vicilin/Provicilin	DA, PQ, DF, IL, ADF, YT, AD, QY, TD	ACE inhibitor, antioxidant, glucose uptake stimulation, α-glucosidase inhibitor, DPP-IV inhibitor
ANRDDNEDLRVLDLAIPV	2038.24	0.27	0.21	Vicilin	AIP, IP, LA, AI, LR, VL, DN, NE, NR, PV	ACE inhibitor, glucose uptake stimulation, DPP-IV inhibitor
LSPGDVFVIPAGHPV	1504.75	0.53	0.09	Vicilin/Provicilin	PG, VF, IPA, LSP, IP, AG, GH, GD, HP, PA, GH, PV, VI	ACE inhibitor, antiamnestic, antithrombotic, regulating the stomach mucosal membrane activity, DPP-IV inhibitor
FVIPAGHPVA	1007.20	0.35	0.09	Vicilin/Provicilin	IPA, IP, AG, GH, HP, VA, PA, PV, VI	ACE inhibitor, DPP-IV inhibitor
LANRDDNEDLRVLDLAIPV	2151.40	0.22	0.23	Vicilin	AIP, IP, LA, AI, LR, VL, LAN, DN, NE, NR, PV	ACE inhibitor, antioxidant, glucose uptake stimulation, DPP-IV inhibitor
SKPHTLFLPQ	1167.37	0.51	0.18	Vicilin/Provicilin	LF, KP, PQ, PH, LP, PHT, FL, HT, SK, TL, LPQ	ACE inhibitor, antioxidant, DPP-IV inhibitor

LAIPVNKPGQL	1149.40	0.45	0.26	Vicilin/Provicilin	PG, AIP, IP, LA, GQ, AI, NK, KP, PV, QL, VN	ACE inhibitor, antioxidant, antiamnestic, neuropeptide, antithrombotic, regulating the stomach mucosal membrane activity, DPP-IV inhibitor
FENLQNY	926.98	0.24	0.07	Vicilin/Convicilin/Provicilin	NY, LQ, NLQ, NL, QN	ACE inhibitor, antiinflammation, DPP-IV inhibitor
IFENLQNYR	1196.33	0.30	0.11	Vicilin/Provicilin	IF, NY, LQ, IFENLQN, YR, NLQ, NL, QN	ACE inhibitor, antiinflammation, neuropeptide, DPP-IV inhibitor
DLAIPV	626.75	0.27	0.11	Vicilin/Provicilin	AIP, IP, LA, AI, PV	ACE inhibitor, DPP-IV inhibitor
IKEGSLLLPN	1083.29	0.32	0.16	Vicilin	LLP, GS, EG, KE, LP, LLL, LL, PN	ACE inhibitor, stimulating vasoactive substance release, glucose uptake stimulation, DPP-IV inhibitor
DLDIFVNSV	1021.14	0.20	0.07	Vicilin/Provicilin	IF, SV, VN	ACE inhibitor, DPP-IV inhibitor
EGSLLLPNYNSRA	1433.58	0.36	0.16	Vicilin	LLP, RA, GS, EG, NY, YN, LP, LLL, LL, SL, PN	ACE inhibitor, stimulating vasoactive substance release, glucose uptake stimulation, DPP-IV inhibitor
DIKEGSLLLPNYNS	1562.74	0.24	0.09	Vicilin	LLP, GS, EG, NY, KE, YN, LP, LLL, LL, SL, PN	ACE inhibitor, stimulating vasoactive substance release, glucose uptake stimulation, DPP-IV inhibitor
VFVIPAGHPVAI	1219.49	0.39	0.09	Vicilin/Provicilin	VF, IPA, IP, AG, GH, AI, HP, VA, PA, PV, VI	ACE inhibitor, DPP-IV inhibitor
IGFGINAEN	934.02	0.20	0.05	Vicilin	GF, IG, GI, FG, AE, IN, NA	ACE inhibitor, DPP-IV inhibitor
GDFELVGQ	863.92	0.28	0.07	Vicilin/Provicilin	VG, GQ, GD, DF, LV, EL	ACE inhibitor, antioxidant, neuropeptide, glucose uptake stimulation, DPP-IV inhibitor
DLDIFVN	834.92	0.38	0.06	Vicilin/Provicilin	IF, VN	ACE inhibitor, DPP-IV inhibitor

EGSLLLPN	841.96	0.42	0.15	Vicilin	LLP, GS, EG, LP, LLL, LL, SL, PN	ACE inhibitor, stimulating vasoactive substance release, glucose uptake stimulation, DPP-IV inhibitor
KSVSSESGPF	1024.10	0.30	0.06	Vicilin/Provicilin	GP, SG, SGP, SE, ES, KS, PF, SV, VS, GPF	ACE inhibitor, antiamnestic, antithrombotic, stimulating vasoactive substance release, regulating the stomach mucosal membrane activity, DPP-IV inhibitor
GDAIKLPAGTIA	1126.32	0. 48	0.15	Vicilin	IA, AG, DA, GT, AI, GD, KL, KLP, LP, IKL, PA, TI	ACE inhibitor, antioxidant, DPP-IV inhibitor
FEITPEKNQQLQDLDIFVN	2291.54	0.29	0.10	Vicilin/Provicilin	IF, EI, LQ, EK, TP, NQ, QD, QL, QQ, VN, PE	ACE inhibitor, α-glucosidase inhibitor, DPP-IV inhibitor
YRLLE	692.81	0.22	0.47	Vicilin/Provicilin	RL, RR, YR, LL	ACE inhibitor, neuropeptide, glucose uptake stimulation, DPP-IV inhibitor
DIKEGSLLLPNYNSR	1718.93	0.34	0.13	Vicilin	LLP, GS, EG, NY, KE, YN, LP, LLL, LL, SL, PN, YN	ACE inhibitor, stimulating vasoactive substance release, glucose uptake stimulation, DPP-IV inhibitor
RSDQENPFI	1105.17	0.42	0.07	Vicilin/Provicilin	NP, DQ, PF, QE	DPP-IV inhibitor
IFENLQ	762.86	0.21	0.07	Vicilin/Provicilin	IF, LQ, NLQ, NL	ACE inhibitor, antiinflammation, DPP-IV inhibitor
DLAIPVNKPGQ	1151.33	0.29	0.16	Vicilin/Provicilin	PG, AIP, IP, LA, GQ, AI, NK, KP, PV, VN	ACE inhibitor, antioxidant, antiamnestic, neuropeptide, antithrombotic, regulating the stomach mucosal membrane activity, DPP-IV inhibitor
IKLPAGTIAY	1046.27	0.20	0.14	Vicilin	AY, IA, AG, GT, KL, IAY, KLP, LP, IKL, PA, TI	ACE inhibitor, antioxidant, DPP-IV inhibitor
FANAQPLQ	887.99	0.46	0.16	Vicilin	PL, LQ, QP, FA, NA	ACE inhibitor, DPP-IV inhibitor
NQQLQDLDIF	1233.34	0.36	0.10	Vicilin/Provicilin	IF, LQ, NQ, QD, QL, QQ	ACE inhibitor, DPP-IV inhibitor
NQQLQDLDIFV	1332.48	0.30	0.12	Vicilin/Provicilin	IF, LQ, NQ, QD, QL, QQ	ACE inhibitor, DPP-IV inhibitor

DIKEGSLLLPN	1198.38	0.33	0.12	Vicilin	LLP, GS, EG, KE, LP, LLL, LL, SL, PN	ACE inhibitor, stimulating vasoactive substance release, glucose uptake stimulation, DPP-IV inhibitor
PQYTDADFI	1069.14	0.41	0.06	Vicilin/Provicilin	DA, PQ, DF, ADF, YT, AD, QY, TD	ACE inhibitor, antioxidant, DPP-IV inhibitor
KEGSLLLPN	970.13	0.35	0.24	Vicilin	LLP, GS, EG, KE, LP, LLL, LL, SL, PN	ACE inhibitor, stimulating vasoactive substance release, glucose uptake stimulation, DPP-IV inhibitor
DIKEGSLLLPNYN	1475.66	0.35	0.10	Vicilin	LLP, GS, EG, NY, KE, YN, LP, LLL, LL, SL, PN	ACE inhibitor, stimulating vasoactive substance release, glucose uptake stimulation, DPP-IV inhibitor
SKIFENLQNYRLLE	1767.01	0.35	0.15	Vicilin/Provicilin	RL, IF, NY, LQ, IFENLQN, LL, YR, NLQ, LL, KI, NL, QN, SK	ACE inhibitor, antiinflammation, neuropeptide, glucose uptake stimulation, DPP-IV inhibitor
DLDIFVNSVDIK	1377.56	0.21	0.07	Vicilin/Provicilin	IF, SV, VD, VN	ACE inhibitor, DPP-IV inhibitor
QSYFANAQPLQRE	1551.68	0.28	0.16	Vicilin	PL, SY, LQ, QP, FA, NA, QS, YF	ACE inhibitor, DPP-IV inhibitor
IPVNKPGQL	965.16	0.35	0.21	Vicilin/Provicilin	PG, IP, GQ, NK, KP, PV, QL, VN	ACE inhibitor, antioxidant, antiamnestic, neuropeptide, antithrombotic, regulating the stomach mucosal membrane activity, DPP-IV inhibitor
SVSSESGPF	895.92	0.39	0.04	Legumin/Vicilin/Provicilin	GP, SGP, SE, ES, PF, SV, VS, GPF	ACE inhibitor, antiamnestic, antithrombotic, stimulating vasoactive substance release, regulating the stomach mucosal membrane activity, DPP-IV inhibitor
IKLPAGTIA	883.10	0.20	0.19	Vicilin	IA, AG, GT, KL, KLP, LP, IKL, PA, TI	ACE inhibitor, antioxidant, DPP-IV inhibitor
LPQYTDADFI	1182.30	0.40	0.07	Vicilin/Provicilin	DA, PQ, DF, LP, ADF, YT, AD, QY, TD, LPQ	ACE inhibitor, antioxidant, α-glucosidase inhibitor, DPP-IV inhibitor
FFEITPEKNQQLQ	1621.81	0.20	0.10	Vicilin/Provicilin	EI, LQ, EK, TP, FF, NQ, QL, QQ, PE	ACE inhibitor, α-glucosidase inhibitor, DPP-IV inhibitor
LFLPQ	616.76	0.64	0.19	Vicilin/Provicilin	LF, PQ, LP, FL, LPQ	ACE inhibitor, DPP-IV inhibitor

LAFPGSSHE	944.01	0.26	0.06	Vicilin/Provicilin	PG, FP, AFP, AF, LA, GS, LAF, HE, SH	ACE inhibitor, antioxidant, antiamnestic, antithrombotic, regulating the stomach mucosal membrane activity, DPP-IV inhibitor
GQLQSFLLSGTQNQ	1520.66	0.29	0.15	Vicilin/Provicilin	GQ, GT, SG, SF, LQ, TQ, LL, FL, NQ, QL, QN, QS, FL	ACE inhibitor, neuropeptide, glucose uptake stimulation, DPP-IV inhibitor
LDLAIPV	739.91	0.26	0.16	Vicilin/Provicilin	AIP, IP, LA, AI, PV	ACE inhibitor, DPP-IV inhibitor
FQTLYENENGHIR	1620.74	0.21	0.09	Vicilin/Provicilin	IR, LY, HIR, GH, NG, FQ, YE, LY, NEN, HI, NE, QT, TL	ACE inhibitor, antioxidant, DPP-IV inhibitor
IKLPAGT	698.86	0.23	0.26	Vicilin/Convicilin	AG, GT, KL, KLP, LP, IKL, PA	ACE inhibitor, antioxidant, DPP-IV inhibitor
AIKLPAGT	769.94	0.33	0.28	Vicilin	AG, GT, AI, KL, KLP, LP, IKL, PA	ACE inhibitor, antioxidant, DPP-IV inhibitor
FFEITPEKNQQLQDLDIF	2225.48	0.25	0.08	Vicilin/Provicilin	IF, EI, LQ, EK, TP, FF, EI, NQ, QD, QL, QQ, PE	ACE inhibitor, α-glucosidase inhibitor, DPP-IV inhibitor
AIVIVTVNEGKGDFELVGQR	2144.46	0.22	0.16	Vicilin/Provicilin	VG, KG, GQ, GK, AI, GD, EG, DF, LV, IV, EL, NE, TV, VI, VN, VT	ACE inhibitor, antioxidant, neuropeptide, glucose uptake stimulation, DPP-IV inhibitor
ASSNLDLLGFGINAENNQR	2033.18	0.44	0.12	Vicilin	GF, GI, FG, LG, LL, LGF, AE, AS, IN, NA, NL, NN, NQ	ACE inhibitor, antioxidant, glucose uptake stimulation, DPP-IV inhibitor
LGFGINAENNQR	1332.44	0.20	0.12	Vicilin/Provicilin	GF, GI, FG, LG, LGF, AE, IN, NA, NN, NQ	ACE inhibitor, antioxidant, DPP-IV inhibitor
SNKFQTLFENENGHIR	1934.10	0.34	0.10	Vicilin	IR, HIR, LF, GH, NG, KF, NK, FQ, IR, NEN, HI, NE, QT, TL	ACE inhibitor, antioxidant, DPP-IV inhibitor
GFGINAENNQR	1219.28	0.20	0.10	Vicilin/Provicilin	GF, GI, FG, AE, IN, NA, NN, NQ	ACE inhibitor, DPP-IV inhibitor
FFEITPEKNPQLQ	1590.80	0.28	0.10	Vicilin	EI, LQ, PQ, EK, TP, FF, NP, QL, PE	ACE inhibitor, α-glucosidase inhibitor, DPP-IV inhibitor
GFGINAENNQRN	1333.38	0.23	0.10	Vicilin/Provicilin	GF, GI, FG, AE, IN, NA, NN, NQ, RN	ACE inhibitor, DPP-IV inhibitor

FQTLFENENGHIR	1604.74	0.33	0.09	Vicilin	IR, HIR, LF, GH, NG, FQ, IR, NEN, HI, NE, QT, TL	ACE inhibitor, antioxidant, DPP-IV inhibitor
EGSLLLPHYNSR	1385.54	0.40	0.13	Vicilin	HY, LLP, GS, EG, PH, YN, LP, LLL, LL, SL, LLPH, PHY	ACE inhibitor, antioxidant, antiinflammation, stimulating vasoactive substance release, glucose uptake stimulation, DPP-IV inhibitor
LAFPGSAQEVDR	1289.41	0.22	0.15	Vicilin	PG, FP, AFP, AF, LA, GS, EV, DR, LAF, QE, VD	ACE inhibitor, antioxidant, antiamnestic, antithrombotic, regulating the stomach mucosal membrane activity, DPP-IV inhibitor
EIKEGSLLLPHYNSR	1755.99	0.30	0.12	Vicilin	HY, LLP, GS, EG, EI, KE, PH, YN, LP, LLL, LL, SL, LLPH, PHY	ACE inhibitor, antioxidant, antiinflammation, stimulating vasoactive substance release, glucose uptake stimulation, DPP-IV inhibitor
GDTIKLPAGTIA	1156.34	0.31	0.15	Vicilin	IA, AG, GT, GD, KL, KLP, LP, IKL, PA, TI	ACE inhibitor, antioxidant, DPP-IV inhibitor
DLDIFVNSVEIK	1391.58	0.20	0.08	Vicilin	IF, EI, VE, SV, VN	ACE inhibitor, α-glucosidase inhibitor, DPP-IV inhibitor
SNKFQTLFENENGHI	1777.91	0.26	0.08	Vicilin	LF, GH, NG, KF, NK, FQ, NEN, GH, HI, NE, QT, TL	ACE inhibitor, antioxidant, DPP-IV inhibitor
FEITPEKNPQLQDL	1671.87	0.21	0.11	Vicilin	EI, LQ, PQ, EK, TP, NP, QD, QL, PE	ACE inhibitor, α-glucosidase inhibitor, DPP-IV inhibitor
FQTLFENENGHI	1448.55	0.27	0.06	Vicilin	LF, GH, NG, FQ, NEN, HI, NE, QT, TL	ACE inhibitor, antioxidant, DPP-IV inhibitor
VEIKEGSLLLPHYNSR	1855.12	0.29	0.14	Vicilin	HY, LLP, GS, EG, EI, VE, KE, PH, YN, LP, LLL, LL, SL, LLPH, PHY	ACE inhibitor, antioxidant, antiinflammation, stimulating vasoactive substance release, glucose uptake stimulation, α-glucosidase inhibitor, DPP-IV inhibitor
LGFGINAENNQRN	1446.54	0.22	0.12	Vicilin/Provicilin	GF, GI, FG, LG, LGF, AE, IN, NA, NN, NQ, RN	ACE inhibitor, antioxidant, DPP-IV inhibitor
FFEITPEKNPQL	1462.67	0.34	0.09	Vicilin	EI, PQ, EK, TP, FF, NP, QL, PE	ACE inhibitor, α-glucosidase inhibitor, DPP-IV inhibitor
KEGSLLLPH	993.17	0.41	0.21	Vicilin	LLP, GS, EG, KE, PH, LP, LLL, LL, SL, LLPH	ACE inhibitor, antioxidant, stimulating vasoactive substance release, glucose uptake stimulation, DPP-IV inhibitor
VNSVEIKEGSLLLPHYNSR	2155.44	0.48	0.13	Vicilin	HY, LLP, GS, EG, EI, VE, KE, PH, YN, LP, LLL, LL, SL, LLPH, PHY, SV, VE, VN	ACE inhibitor, antioxidant, antiinflammation, stimulating vasoactive substance release, glucose uptake stimulation, α-glucosidase inhibitor, DPP-IV inhibitor
DLAIPVNRPGQLQ	1420.63	0.33	0.22	Vicilin	PG, AIP, IP, RP, LA, GQ, AI, PV, QL, VN	ACE inhibitor, antiamnestic, neuropeptide, antithrombotic, regulating the stomach mucosal membrane activity, DPP-IV inhibitor

VEIKEGSLLLPH	1334.58	0.25	0.13	Vicilin	LLP, GS, EG, EI, VE, KE, PH, LP, LLL, LL, SL, LLPH	ACE inhibitor, stimulating vasoactive substance release, glucose uptake stimulation, α-glucosidase inhibitor, DPP-IV inhibitor
IKEGSLLLPH	1106.33	0.35	0.14	Vicilin	LLP, GS, EG, KE, PH, LP, LLL, LL, SL, LLPH	ACE inhibitor, stimulating vasoactive substance release, glucose uptake stimulation, DPP-IV inhibitor
KNPQLQDL	955.08	0.30	0.27	Vicilin/Provicilin	LQ, PQ, NP, QD, QL	ACE inhibitor, DPP-IV inhibitor
SGNQNQQNYLSGFSK	1671.74	0.36	0.08	Vicilin	YL, GF, SG, NY, NQ, QN, QQ, SK	ACE inhibitor, neuropeptide, DPP-IV inhibitor
SSNLDLLGFGINAENNQR	1962.11	0.46	0.11	Vicilin	GF, GI, FG, LG, LL, LGF, AE, GI, IN, NA, NL, NN, NQ	ACE inhibitor, antioxidant, glucose uptake stimulation, DPP-IV inhibitor
EITPEKNPQLQDLDIFVN	2113.35	0.27	0.10	Vicilin	IF, EI, LQ, PQ, EK, TP, NP, QD, QL, VN, PE	ACE inhibitor, α-glucosidase inhibitor, DPP-IV inhibitor
EGSLLLPH	865.00	0.50	0.13	Vicilin	LLP, GS, EG, PH, LP, LLL, LL, SL, LLPH	ACE inhibitor, antioxidant, stimulating vasoactive substance release, glucose uptake stimulation, DPP-IV inhibitor
SRGPIYSNEFGK	1354.49	0.57	0.08	Vicilin	GP, FGK, IY, FG, GK, RG, EF, NE, PI, YS, EF	ACE inhibitor, antioxidant, hypolipidemic, antiamnestic, antithrombotic, regulating the stomach mucosal membrane activity, DPP-IV inhibitor
ASSNLDLLGFGINAENNQRN	2147.29	0.49	0.12	Vicilin	GF, GI, FG, LG, LL, LGF, AE, GI, IN, NA, NL, NN, NQ	ACE inhibitor, antioxidant, glucose uptake stimulation, DPP-IV inhibitor
DLAIPVNRPGQ	1179.34	0.36	0.18	Vicilin	PG, AIP, IP, RP, LA, GQ, AI, NR, PV, VN	ACE inhibitor, antiamnestic, neuropeptide, antithrombotic, regulating the stomach mucosal membrane activity, DPP-IV inhibitor
SVEIKEGSLLLPH	1421.66	0.39	0.11	Vicilin	LLP, GS, EG, EI, VE, KE, PH, LP, LLL, LL, SL, LLPH, SV	ACE inhibitor, antioxidant, stimulating vasoactive substance release, glucose uptake stimulation, α-glucosidase inhibitor, DPP-IV inhibitor
GDTIKLPAGTI	1085.27	0.33	0.13	Vicilin	AG, GT, GD, KL, KLP, LP, IKL, PA, TI	ACE inhibitor, antioxidant, DPP-IV inhibitor
EGSLLLPHYNS	1229.35	0.28	0.08	Vicilin	HY, LLP, GS, EG, PH, YN, LP, LLL, LL, SL, LLPH, PHY	ACE inhibitor, antioxidant, antiinflammation, stimulating vasoactive substance release, glucose uptake stimulation, DPP-IV inhibitor

GDTIKLPAGT	972.11	0.33	0.17	Vicilin/Convicilin	AG, GT, GD, KL, KLP, LP, IKL, PA, TI	ACE inhibitor, antioxidant, DPP-IV inhibitor
FFEITPEKNPQLQDL	1819.04	0.30	0.10	Vicilin	EI, LQ, PQ, EK, TP, FF, NP, QD, QL, PE	ACE inhibitor, α-glucosidase inhibitor, DPP-IV inhibitor
AIPVNRPGQLQ	1192.38	0.31	0.26	Vicilin	PG, AIP, IP, RP, GQ, AI, LQ, NR, PV, QL, VN	ACE inhibitor, antiamnestic, neuropeptide, antithrombotic, regulating the stomach mucosal membrane activity, DPP-IV inhibitor
TIFLPQ	717.86	0.40	0.09	Vicilin/Convicilin	IF, PQ, IFL, LP, FL, TI, LPQ	ACE inhibitor, DPP-IV inhibitor
LAIPVNRPGQL	1177.41	0.49	0.30	Vicilin	PG, AIP, IP, RP, LA, GQ, AI, NR, PV, QL, VN	ACE inhibitor, antiamnestic, neuropeptide, antithrombotic, regulating the stomach mucosal membrane activity, DPP-IV inhibitor
EIKEGSLLLPH	1235.45	0.28	0.12	Vicilin	LLP, GS, EG, EI, KE, PH, LP, LLL, LL, SL, LLPH	ACE inhibitor, antioxidant, stimulating vasoactive substance release, glucose uptake stimulation, DPP-IV inhibitor
VLDLAIPVNRPGQL	1504.79	0.43	0.27	Vicilin	PG, AIP, IP, RP, LA, GQ, AI, VL, NR, PV, QL, VN	ACE inhibitor, antiamnestic, neuropeptide, antithrombotic, glucose uptake stimulation, regulating the stomach mucosal membrane activity, DPP-IV inhibitor
LAIPVNRPGQ	1064.25	0.32	0.25	Vicilin	PG, AIP, IP, RP, LA, GQ, AI, NR, PV, VN	ACE inhibitor, antiamnestic, neuropeptide, antithrombotic, regulating the stomach mucosal membrane activity, DPP-IV inhibitor
KEGSLLLPHYNSRAI	1697.95	0.46	0.15	Vicilin	HY, LLP, RA, GS, AI, EG, KE, PH, YN, LP, LLL, LL, SL, LLPH, PHY	ACE inhibitor, antioxidant, antiinflammation, stimulating vasoactive substance release, glucose uptake stimulation, DPP-IV inhibitor
KNPQLQDLDIFV	1429.64	0.42	0.13	Vicilin/Provicilin	IF, LQ, PQ, NP, QD, QL	ACE inhibitor, DPP-IV inhibitor
DLAIPVNRPGQLQS	1507.71	0.23	0.18	Vicilin	PG, AIP, IP, RP, LA, GQ, AI, NR, PV, QL, QS, VN	ACE inhibitor, antiamnestic, neuropeptide, antithrombotic, regulating the stomach mucosal membrane activity, DPP-IV inhibitor
SRSDPQNPF	1047.09	0.67	0.08	Vicilin	PQ, NP, DP, PF, QN	ACE inhibitor, DPP-IV inhibitor
ELAFPGSAQEVDR	1418.53	0.21	0.12	Vicilin	PG, FP, AFP, AF, LA, GS, EV, DR, EL, LAF, QE, VD	ACE inhibitor, antioxidant, antiamnestic, antithrombotic, regulating the stomach mucosal membrane activity, DPP-IV inhibitor

ELAFPGSAQ	919.00	0.24	0.09	Vicilin	PG, FP, AFP, AF, LA, GS, EL, LAF	ACE inhibitor, antioxidant, antiamnestic, antithrombotic, regulating the stomach mucosal membrane activity, DPP-IV inhibitor
GDTIKLPAG	871.00	0.38	0.15	Vicilin/Convicilin	AG, GD, KL, KLP, LP, IKL, PA, TI	ACE inhibitor, antioxidant, DPP-IV inhibitor
SKPHTIFLPQ	1167.37	0.50	0.10	Vicilin	IF, KP, PQ, PH, IFL, LP, PHT, HT, SK, TI, LPQ	ACE inhibitor, antioxidant, DPP-IV inhibitor
VLDLAIPVNRPGQLQSF	1867.18	0.32	0.19	Vicilin	PG, AIP, IP, RP, LA, GQ, AI, SF, LQ, VL, NR, PV, QL, QS, VN	ACE inhibitor, antiamnestic, neuropeptide, antithrombotic, glucose uptake stimulation, regulating the stomach mucosal membrane activity, DPP-IV inhibitor
AIPVNRPGQLQS	1867.18	0.21	0.19	Vicilin	PG, AIP, IP, RP, GQ, AI, LQ, NR, PV, QL, QS, VN	ACE inhibitor, antiamnestic, neuropeptide, antithrombotic, DPP-IV inhibitor
LAIPVNRPGQLQ	1305.54	0.26	0.30	Vicilin	PG, AIP, IP, RP, LA, GQ, AI, PV, QL, VN	ACE inhibitor, antiamnestic, neuropeptide, antithrombotic, regulating the stomach mucosal membrane activity, DPP-IV inhibitor
LAFPGSAQ	789.89	0.30	0.11	Vicilin	PG, FP, AFP, AF, LA, GS, LAF	ACE inhibitor, antioxidant, antiamnestic, antithrombotic, regulating the stomach mucosal membrane activity, DPP-IV inhibitor
NEGKGDFELV	1107.19	0.21	0.09	Vicilin/Provicilin	KG, GK, GD, EG, DF, LV, EL, NE	ACE inhibitor, antioxidant, glucose uptake stimulation, DPP-IV inhibitor
ASSNLDLLG	888.97	0.60	0.12	Vicilin	LG, LL, AS, NL	ACE inhibitor, glucose uptake stimulation, DPP-IV inhibitor
LGFGINAEN	934.02	0.20	0.07	Vicilin/Provicilin	GF, GI, FG, LG, LGF, AE, IN, NA	ACE inhibitor, antioxidant, DPP-IV inhibitor
GDTIKLPAGTIAY	1319.52	0.30	0.13	Vicilin	AY, IA, AG, GT, GD, KL, IAY, KLP, LP, IKL, PA, TI	ACE inhibitor, antioxidant, antiinflammation, antiamnestic, neuropeptide, antibacterial, DPP-IV inhibitor
KNPQLQDLDI	1183.33	0.22	0.14	Vicilin/Provicilin	LQ, PQ, NP, QD, QL	ACE inhibitor, DPP-IV inhibitor
KASSNLDLLG	1017.15	0.49	0.18	Vicilin	LG, KA, LL, AS, NL	ACE inhibitor, glucose uptake stimulation, DPP-IV inhibitor
KNPQLQDLDIFVN	1543.74	0.44	0.12	Vicilin/Provicilin	IF, LQ, PQ, NP, QD, QL, VN	ACE inhibitor, DPP-IV inhibitor

FEITPEKNPQL	1315.49	0.23	0.11	Vicilin	EI, PQ, EK, TP, NP, QL, PE	ACE inhibitor, *α*-glucosidase inhibitor, DPP-IV inhibitor
FENENGHIRL	1228.33	0.40	0.09	Vicilin/Convicilin	RL, IR, HIRL, HIR, GH, NG, NEN, HI, NE	ACE inhibitor, antioxidant, neuropeptide, DPP-IV inhibitor
RSDPQNPF	960.01	0.71	0.09	Vicilin	PQ, NP, DP, PF, QN	ACE inhibitor, DPP-IV inhibitor
RVLDLAIPVNRPGQLQ	1789.11	0.20	0.39	Vicilin	PG, AIP, IP, RP, LA, GQ, AI, LQ, VL, NR, PV, QL, VN	ACE inhibitor, antiamnestic, neuropeptide, antithrombotic, glucose uptake stimulation, regulating the stomach mucosal membrane activity, DPP-IV inhibitor
GSLLLPH	735.88	0.61	0.19	Vicilin	LLP, GS, PH, LP, LLL, LL, SL, LLPH	ACE inhibitor, antioxidant, stimulating vasoactive substance release, glucose uptake stimulation, DPP-IV inhibitor
LLLPH	591.75	0.41	0.40	Vicilin	LLP, PH, LP, LLL, LL, LLPH	ACE inhibitor, antioxidant, stimulating vasoactive substance release, glucose uptake stimulation, DPP-IV inhibitor
SRGPIYSNEFG	1226.31	0.54	0.06	Vicilin	GP, IY, FG, RG, EF, NE, PI, YS	ACE inhibitor, antioxidant, hypolipidemic, antiamnestic, antithrombotic, regulating the stomach mucosal membrane activity, DPP-IV inhibitor
FFEITPEKNPQLQDLDIF	2194.47	0.41	0.08	Vicilin	IF, EI, LQ, PQ, EK, TP, FF, NP, QD, QL, PE	ACE inhibitor, *α*-glucosidase inhibitor, DPP-IV inhibitor
VNEGKGDFEL	1107.19	0.20	0.09	Vicilin/Provicilin	KG, GK, GD, EG, DF, EL, NE, VN	ACE inhibitor, antioxidant, DPP-IV inhibitor
DLAIPVNRPGQL	1292.50	0.55	0.22	Vicilin	PG, AIP, IP, RP, LA, GQ, AI, NR, PV, QL, VN	ACE inhibitor, antiamnestic, neuropeptide, antithrombotic, regulating the stomach mucosal membrane activity, DPP-IV inhibitor
DDNEELRVL	1102.17	0.21	0.17	Vicilin	LR, VR, EE, EL, DN, NE, RV	ACE inhibitor, antioxidant, stimulating vasoactive substance release, glucose uptake stimulation, DPP-IV inhibitor
GDTIKLPAGTIAYL	1432.68	0.54	0.15	Vicilin	YL, AY, IA, AG, GT, GD, KL, IAY, KLP, LP, IKL, PA, TI	ACE inhibitor, antioxidant, neuropeptide, DPP-IV inhibitor
IFLPQ	616.76	0.64	0.08	Vicilin/Convicilin	IF, PQ, IFL, LP, FL, LPQ	ACE inhibitor, DPP-IV inhibitor

KNPQLQDLD	1070.17	0.20	0.18	Vicilin/Provicilin	LQ, PQ, NP, QD, QL	ACE inhibitor, DPP-IV inhibitor
LSSGDVFVIPA	1104.27	0.48	0.07	Vicilin	VF, IPA, IP, SG, GD, LSSGDVF, PA, VI	ACE inhibitor, DPP-IV inhibitor
FFEITPEKNPQ	1349.51	0.21	0.08	Vicilin	EI, PQ, EK, TP, FF, NP, PE	ACE inhibitor, α-glucosidase inhibitor, DPP-IV inhibitor
SEAGLTETWNPNHPE	1681.74	0.21	0.10	Legumin	GL, AG, EA, TE, HP, SE, TW, NP, WN, ET, LT, NH, PN	ACE inhibitor, antioxidant, stimulating vasoactive substance release, α-glucosidase inhibitor, DPP-IV inhibitor
LGGNPEIEFPETQQK	1686.84	0.23	0.09	Legumin	FP, GG, LG, QK, EI, IE, TKQ, EF, NP, ET, QQ, TQ, PE	ACE inhibitor, hypolipidemic, α-glucosidase inhibitor, DPP-IV inhibitor
RNGIYAPHWNINA	1525.69	0.49	0.12	Legumin	IY, AP, YA, GI, NG, PH, PHW, WN, HW, IN, NA, NG, RN	ACE inhibitor, antioxidant, DPP-IV inhibitor
RFSKGDVIAIPPGIP	1566.86	0.54	0.10	Legumin	IPP, PG, RF, AIP, IA, IP, GI, KG, AI, GD, IAIP, AIPP, IAIPP, PP, PPG, SK, VI	ACE inhibitor, antiinflammation, antiamnestic, α-amylase inhibitor, antithrombotic, regulating the stomach mucosal membrane activity, α-glucosidase inhibitor, DPP-IV inhibitor
TIDPNGLHLPSYSPSPQ	1822.99	0.25	0.07	Legumin	LHLP, PSY, GL, HL, NG, SY, PQ, LP, LH, LHL, SP, DP, PN, PS, TI, YS	ACE inhibitor, antioxidant, DPP-IV inhibitor
ATPGEVLANAFGLR	1415.61	0.61	0.30	Legumin	PG, AF, LA, GL, FG, GE, EV, TP, LR, VL, LAN, AT, NA	ACE inhibitor, antioxidant, antiamnestic, antithrombotic, glucose uptake stimulation, regulating the stomach mucosal membrane activity, DPP-IV inhibitor
GLTETWNPNHPEL	1507.62	0.30	0.16	Legumin	GL, TE, HP, EL, PEL, TW, TETWNPNHPEL, NP, WN, ET, LT, NH, PN	ACE inhibitor, antioxidant, α-glucosidase inhibitor, DPP-IV inhibitor
GQIVKVEDGLHIISPEL	1847.14	0.26	0.09	Legumin	VK, GL, GQ, DG, VE, DGL, IV, II, LH, EL, LHI, VKV, PEL, SP, HI, KV, QI, PE	ACE inhibitor, antioxidant, neuropeptide, glucose uptake stimulation, α-glucosidase inhibitor, DPP-IV inhibitor
RTIDPNGLHLPSYSPSPQ	1979.18	0.23	0.10	Legumin	LHLP, PSY, GL, HL, NG, SY, PQ, LP, LH, LHL, SP, DP, PN, PS, TI, YS	ACE inhibitor, antioxidant, DPP-IV inhibitor
IRENIARPSRGDL	1496.69	0.27	0.21	Legumin	IR, IA, RP, GD, AR, RG, RGD, PS	ACE inhibitor, antioxidant, antithrombotic, DPP-IV inhibitor

LTETWNPNHPEL	1450.57	0.21	0.16	Legumin	TE, HP, EL, PEL, TW, TETWNPNHPEL, NP, WN, ET, LT, NH, PN	ACE inhibitor, antioxidant, *α*-glucosidase inhibitor, DPP-IV inhibitor
ISTVNSLTLPILR	1426.72	0.25	0.20	Legumin	IL, ST, LR, LPILR, LP, SL, LT, PI, TL, TV, VN	ACE inhibitor, antioxidant, hypotensive, glucose uptake stimulation, DPP-IV inhibitor
ENIARPSRGDLYNSGAGR	1933.07	0.29	0.17	Legumin	LY, IA, RP, GA, AG, GR, SG, GD, YN, RGD, PS	ACE inhibitor, antioxidant, antithrombotic, DPP-IV inhibitor
YLGGNPEIEFPET	1465.58	0.25	0.06	Legumin	FP, YL, GG, LG, EI, IE, EF, YLG, FN, NP, EI, ET, PE	ACE inhibitor, antioxidant, hypolipidemic, neuropeptide, *α*-glucosidase inhibitor, DPP-IV inhibitor
TETWNPNHPEL	1337.41	0.27	0.13	Legumin	TE, HP, EL, PEL, TW, TETWNPNHPEL, NP, WN, ET, LT, NH, PN	ACE inhibitor, antioxidant, *α*-glucosidase inhibitor, DPP-IV inhibitor
RFSKGDVIAIPPGIPY	1730.04	0.47	0.09	Legumin	IPP, PG, RF, AIP, IA, IP, GI, KG, AI, GD, IAIP, AIPP, IAIPP, PP, PPG, SK, VI	ACE inhibitor, antiinflammation, antiamnestic, *α*-amylase inhibitor, antithrombotic, regulating the stomach mucosal membrane activity, *α*-glucosidase inhibitor, DPP-IV inhibitor
GGNPEIEFPETQQK	1573.68	0.27	0.08	Legumin	FP, GG, QK, EI, IE, TQ, EF, FN, NP, ET, QQ, TQ, PE	ACE inhibitor, hypolipidemic, *α*-glucosidase inhibitor, DPP-IV inhibitor
RNGIYAPHWNINAN	1639.79	0.49	0.13	Legumin	IY, AP, YA, GI, NG, PH, PHW, WN, HW, IN, NA, NG, PH, RN	ACE inhibitor, antioxidant, DPP-IV inhibitor
PNGLHLPSYSPSPQ	1493.64	0.36	0.08	Legumin	LHLP, PSY, GL, HL, NG, SY, PQ, LP, LH, LHL, SP, PN, PS, YS	ACE inhibitor, antioxidant, DPP-IV inhibitor
ENIARPSRGDL	1227.34	0.36	0.17	Legumin	IA, RP, GD, AR, RG, RGD, PS	ACE inhibitor, antithrombotic, DPP-IV inhibitor
LGGNPEIEFPET	1302.40	0.33	0.06	Legumin	FP, GG, LG, EI, IE, EF, NP, ET, PE	ACE inhibitor, hypolipidemic, *α*-glucosidase inhibitor, DPP-IV inhibitor
FSKGDVIAIPPGIPY	1573.85	0.57	0.06	Legumin	IPP, PG, RF, AIP, IA, IP, GI, KG, AI, GD, IAIP, AIPP, IAIPP, PP, PPG, SK, VI	ACE inhibitor, antiinflammation, antiamnestic, *α*-amylase inhibitor, antithrombotic, regulating the stomach mucosal membrane activity, *α*-glucosidase inhibitor, DPP-IV inhibitor

FSKGDVIAIPPGIP	1410.68	0.62	0.07	Legumin	IPP, PG, RF, AIP, IA, IP, GI, KG, AI, GD, IAIP, AIPP, IAIPP, PP, PPG, SK, VI	ACE inhibitor, antiinflammation, antiamnestic, α-amylase inhibitor, antithrombotic, regulating the stomach mucosal membrane activity, α-glucosidase inhibitor, DPP-IV inhibitor
ETWNPNHPE	1123.15	0.25	0.09	Legumin	HP, TW, WN, ET, NH, PN, PE	ACE inhibitor, antioxidant, α-glucosidase inhibitor, DPP-IV inhibitor
NALEPDHRVESEAGLT	1737.84	0.20	0.14	Legumin	GL, AG, EA, VE, ALEPDHR, EP, AL, ES, HR, LT, NA, VE	ACE inhibitor, antioxidant, stimulating vasoactive substance release, α-glucosidase inhibitor, DPP-IV inhibitor
AIPPGIP	663.81	0.79	0.07	Legumin	IPP, PG, AIP, IP, GI, AI, AIPP, PP, IP, PPG	ACE inhibitor, antiinflammation, antiamnestic, α-amylase inhibitor, antithrombotic, regulating the stomach mucosal membrane activity, α-glucosidase inhibitor, DPP-IV inhibitor
SKGDVIAIPPGIPY	1426.68	0.52	0.07	Legumin	IPP, PG, AIP, IA, IP, GI, KG, AI, GD, IAIP, AIPP, IAIPP, PP, PPG, SK, VI	ACE inhibitor, antiinflammation, antiamnestic, α-amylase inhibitor, antithrombotic, regulating the stomach mucosal membrane activity, α-glucosidase inhibitor, DPP-IV inhibitor
ALEPDHRVESEAGL	1522.63	0.23	0.13	Legumin	GL, AG, EA, VE, ALEP, SE, ALEPDHR, EP, AL, ES, HR, LT, NA, VE	ACE inhibitor, antioxidant, stimulating vasoactive substance release, α-glucosidase inhibitor, DPP-IV inhibitor
LIFIIQGK	931.19	0.32	0.08	Legumin	IF, GK, QG, LI, II, IQ	ACE inhibitor, glucose uptake stimulation, DPP-IV inhibitor
AAVSHVNQVFR	1227.39	0.25	0.21	Legumin	VF, AA, FR, AV, HV, NQ, QV, SH, VF, VN, VS	ACE inhibitor, hypotensive, DPP-IV inhibitor
NINANSLL	857.96	0.28	0.12	Legumin	LL, SL, IN, NA	glucose uptake stimulation, DPP-IV inhibitor
AGLTETWNPNHPEL	1578.70	0.31	0.17	Legumin	GL, AG, TE, HP, EL, PEL, TW, TETWNPNHPEL, NP, WN, ET, LT, NH, PN	ACE inhibitor, antioxidant, α-glucosidase inhibitor, DPP-IV inhibitor
IPPGIPY	755.91	0.74	0.05	Legumin/ Glycinin	IPP, PG, IP, GI, IPY, PP, PPG	ACE inhibitor, antiinflammation, antiamnestic, α-amylase inhibitor, antithrombotic, regulating the stomach mucosal membrane activity, α-glucosidase inhibitor, DPP-IV inhibitor
FYLGGNPEIEFPET	1612.76	0.30	0.06	Legumin	FP, YL, FY, GG, LG, EI, IE, EF, YLG, NP, ET, PE, EF	ACE inhibitor, antioxidant, hypolipidemic, neuropeptide, α-glucosidase inhibitor, DPP-IV inhibitor
GLHLPSYSPSPQ	1282.42	0.33	0.07	Legumin	LHLP, PSY, GL, HL, SY, PQ, LP, LH, LHL, SP, PN, PS, YS	ACE inhibitor, antioxidant, DPP-IV inhibitor

GDVIAIPPGIP	1048.25	0.66	0.06	Legumin	IPP, PG, AIP, IA, IP, GI, KG, AI, GD, IAIP, AIPP, IAIPP, PP, PPG, SK, VI	ACE inhibitor, antiinflammation, antiamnestic, α-amylase inhibitor, antithrombotic, regulating the stomach mucosal membrane activity, α-glucosidase inhibitor, DPP-IV inhibitor
SKGDVIAIPPGIP	1263.50	0.56	0.07	Legumin	IPP, PG, AIP, IA, IP, GI, KG, AI, GD, IAIP, AIPP, IAIPP, PP, PPG, SK, VI	ACE inhibitor, antiinflammation, antiamnestic, α-amylase inhibitor, antithrombotic, regulating the stomach mucosal membrane activity, α-glucosidase inhibitor, DPP-IV inhibitor
GGNPEIEFPETQQ	1445.51	0.20	0.06	Legumin	FP, GG, EI, IE, TQ, EF, FP, NP, EI, ET, QQ, TQ, PE	ACE inhibitor, hypolipidemic, α-glucosidase inhibitor, DPP-IV inhibitor
RTIDPNGL	884.99	0.31	0.17	Legumin	GL, NG, DP, PN, TI	ACE inhibitor, DPP-IV inhibitor
LGGNPEIEFPETQ	1430.53	0.24	0.07	Legumin	FP, GG, LG, EI, IE, TQ, EF, FP, NP, ET, PE, EF	ACE inhibitor, hypolipidemic, DPP-IV inhibitor
TETWNPNHPELK	1465.59	0.21	0.20	Legumin	TE, HP, EL, PEL, TW, TETWNPNHPEL, NP, WN, ET, NH, PN	ACE inhibitor, antioxidant, α-glucosidase inhibitor, DPP-IV inhibitor
WNINAN	730.78	0.38	0.14	Legumin	WN, IN, NA	DPP-IV inhibitor
YLGGNPEIEFPE	1364.47	0.25	0.06	Legumin	FP, YL, GG, LG, EI, IE, EF, YLG, NP, PE	ACE inhibitor, antioxidant, neuropeptide, hypolipidemic, α-glucosidase inhibitor, DPP-IV inhibitor
ISTVNSLTLPIL	1270.53	0.27	0.13	Legumin	IL, ST, LP, SL, LT, PI, TL, TV, VN	ACE inhibitor, glucose uptake stimulation, DPP-IV inhibitor
DVIAIPPGIPY	1154.37	0.52	0.06	Legumin	IPP, PG, AIP, IA, IP, GI, KG, AI, GD, IAIP, AIPP, IAIPP, PP, PPG, SK, VI	ACE inhibitor, antiinflammation, antiamnestic, α-amylase inhibitor, antithrombotic, regulating the stomach mucosal membrane activity, α-glucosidase inhibitor, DPP-IV inhibitor
LHIISPEL	921.10	0.22	0.07	Legumin	II, LH, EL, LHI, PEL, SP, HI, PE	antioxidant, glucose uptake stimulation, α-glucosidase inhibitor, DPP-IV inhibitor
TIDPNGLHLPS	1163.30	0.25	0.07	Legumin	EK, KE, LEK, EEE, EE, EL	ACE inhibitor, antioxidant, stimulating vasoactive substance release, DPP-IV inhibitor
FNVEFLAHS	1063.18	0.27	0.07	Legumin	LA, VE, AH, EF, FL, FN, HS, NV	ACE inhibitor, antioxidant, hypolipidemic, α-glucosidase inhibitor, DPP-IV inhibitor
APHWNINAN	1036.11	0.68	0.12	Legumin	AP, PH, PHW, WN, HW, IN, NA	ACE inhibitor, antioxidant, DPP-IV inhibitor

YRNGIYAPHWNINANSLL	2116.37	0.44	0.13	Legumin	IY, AP, YA, GI, NG, PH, LL, YR, SL, PHW, LL, SL, WN, HW, IN, NA, RN	ACE inhibitor, antioxidant, neuropeptide, glucose uptake stimulation, DPP-IV inhibitor
HLPSYSPSPQ	1112.21	0.33	0.06	Legumin	PSY, HL, SY, PQ, LP, SP, PS, YS	ACE inhibitor, antioxidant, DPP-IV inhibitor
DVIAIPPGIP	991.20	0.57	0.06	Legumin	IPP, PG, AIP, IA, IP, GI, KG, AI, IAIP, AIPP, IAIPP, PP, PPG	ACE inhibitor, antiinflammation, antiamnestic, α-amylase inhibitor, antithrombotic, regulating the stomach mucosal membrane activity, α-glucosidase inhibitor, DPP-IV inhibitor
IAIPPGIPY	940.15	0.65	0.06	Legumin	IPP, PG, AIP, IA, IP, GI, AI, IAIP, AIPP, IAIPP, PP, PPG	ACE inhibitor, antiinflammation, antiamnestic, α-amylase inhibitor, antithrombotic, regulating the stomach mucosal membrane activity, α-glucosidase inhibitor, DPP-IV inhibitor
HIISPEL	807.94	0.25	0.06	Legumin	II, EL, PEL, SP, HI, PE	antioxidant, glucose uptake stimulation, α-glucosidase inhibitor, DPP-IV inhibitor
IAIPPGIP	776.97	0.67	0.06	Legumin	IPP, PG, AIP, IA, IP, GI, AI, IAIP, AIPP, IAIPP, PP, PPG	ACE inhibitor, antiinflammation, antiamnestic, α-amylase inhibitor, antithrombotic, regulating the stomach mucosal membrane activity, α-glucosidase inhibitor, DPP-IV inhibitor
FYLGGNPEIE	1138.24	0.20	0.06	Legumin	YL, FY, GG, LG, EI, IE, YL, YLG, NP, PE	ACE inhibitor, antioxidant, neuropeptide, α-glucosidase inhibitor, DPP-IV inhibitor
AIPPGIPY	826.99	0.75	0.06	Legumin	IPP, PG, AIP, IA, IP, GI, AI, AIPP, IPY, PP, PPG	ACE inhibitor, antiinflammation, antiamnestic, α-amylase inhibitor, antithrombotic, regulating the stomach mucosal membrane activity, α-glucosidase inhibitor, DPP-IV inhibitor
VVVPQNFV	901.07	0.20	0.18	Legumin	VP, NF, PQ, VVV, VV, QN	ACE inhibitor, anticancer, DPP-IV inhibitor
GDVIAIPPGIPY	1211.42	0.62	0.06	Legumin	IPP, PG, AIP, IA, IP, GI, AI, GD, IAIP, AIPP, IAIPP, PP, PPG	ACE inhibitor, antiinflammation, antiamnestic, α-amylase inhibitor, antithrombotic, regulating the stomach mucosal membrane activity, α-glucosidase inhibitor, DPP-IV inhibitor
GDTIKLPAGTTSYL	1436.62	0.33	0.16	Convicilin	YL, AG, GT, SD, SY, KL, KLP, LP, IKL, PA, TI, TS, TT	ACE inhibitor, antioxidant, neuropeptide, DPP-IV inhibitor

SDLFENLQNYRLVE	1739.90	0.27	0.13	Convicilin	RL, LF, NY, LVE, VE, LQ, LV, YR, NLQ, NL, QN	ACE inhibitor, antiinflammation, neuropeptide, glucose uptake stimulation, α-glucosidase inhibitor, DPP-IV inhibitor
DLVIPVNGPGK	1108.30	0.45	0.15	Convicilin	PG, GP, IP, GK, NG, LV, PV, VI, VN	ACE inhibitor, antiamnestic, antithrombotic, glucose uptake stimulation, regulating the stomach mucosal membrane activity, DPP-IV inhibitor
FLTLFENENGHI	1433.58	0.21	0.07	Convicilin	LF, GH, NG, NEN, FL, HI, LT, NE, TL	ACE inhibitor, antioxidant, DPP-IV inhibitor
LVIPVNGPGK	993.21	0.37	0.21	Convicilin	PG, GP, IP, GK, NG, LV, PV, VI, VN	ACE inhibitor, antiamnestic, antithrombotic, glucose uptake stimulation, regulating the stomach mucosal membrane activity, DPP-IV inhibitor
HGEWRPSYE	1160.21	0.36	0.09	Convicilin	PSY, RP, HG, GE, SY, EW, YE, RP, WRP, WR, PS	ACE inhibitor, DPP-IV inhibitor
DDEEDLRLV	1103.15	0.25	0.14	Convicilin	RL, LR, DEE, LV, EE	ACE inhibitor, antithrombotic, stimulating vasoactive substance release, glucose uptake stimulation, DPP-IV inhibitor
DLVIPVNGPGKFEA	1455.67	0.37	0.11	Convicilin	PG, GP, IP, GK, EA, NG, LV, PV, VI, VN	ACE inhibitor, antiamnestic, antithrombotic, glucose uptake stimulation, regulating the stomach mucosal membrane activity, α-glucosidase inhibitor, DPP-IV inhibitor
SDLFENLQNYR	1398.49	0.31	0.11	Convicilin	LF, NY, LQ, YR, NLQ, NL, QN	ACE inhibitor, antiinflammation, neuropeptide, DPP-IV inhibitor
GEWRPSYE	1023.07	0.35	0.10	Convicilin	PSY, RP, GE, SY, EW, YE, RP, WRP, WR, PS	ACE inhibitor, DPP-IV inhibitor
SDLFENLQN	1079.13	0.26	0.08	Convicilin	LF, LQ, NLQ, NL, QN	ACE inhibitor, antiinflammation, DPP-IV inhibitor
DLVIPVNGPGKFEAFDLAK	2030.35	0.63	0.14	Convicilin	PG, GP, IP, AF, LA, GK, EA, NG, KF, LV, PV, VI, VN	ACE inhibitor, antiamnestic, antithrombotic, glucose uptake stimulation, regulating the stomach mucosal membrane activity, α-glucosidase inhibitor, DPP-IV inhibitor
LVIPVNGPGKFEA	1340.59	0.32	0.14	Convicilin	PG, GP, IP, GK, EA, NG, KF, LV, PV, VI, VN	ACE inhibitor, antiamnestic, antithrombotic, glucose uptake stimulation, regulating the stomach mucosal membrane activity, α-glucosidase inhibitor, DPP-IV inhibitor
FLPQHIDADLILV	1493.77	0.53	0.10	Convicilin	DA, PQ, IL, LP, LV, LI, FL, AD, HI, QH, LPQ	ACE inhibitor, glucose uptake stimulation, α-glucosidase inhibitor, DPP-IV inhibitor

VDLVIPVNGPGK	1207.44	0.38	0.17	Convicilin	PG, GP, IP, GK, EA, NG, NG, LV, PV, VD, VI, VN	ACE inhibitor, antiamnestic, antithrombotic, glucose uptake stimulation, regulating the stomach mucosal membrane activity, DPP-IV inhibitor
GDTIKLPAGTT	1073.21	0.27	0.20	Convicilin	AG, GT, GD, KL, KLP, LP, IKL, PA, TI, TT	ACE inhibitor, antioxidant, DPP-IV inhibitor
LFENLQNYRL	1309.49	0.50	0.21	Convicilin	RL, LF, NY, LQ, YR, NLQ, NL, QN	ACE inhibitor, antiinflammation, neuropeptide, DPP-IV inhibitor
LPQHIDADLILV	1346.59	0.46	0.11	Convicilin	DA, PQ, IL, LP, LV, LI, AD, HI, QH, LPQ	ACE inhibitor, glucose uptake stimulation, α-glucosidase inhibitor, DPP-IV inhibitor
SDLFENLQNY	1242.31	0.23	0.07	Convicilin	LF, NY, LQ, NLQ, NL, QN	ACE inhibitor, antiinflammation, DPP-IV inhibitor
LVIPVNGPG	865.04	0.28	0.14	Convicilin	PG, GP, IP, NG, LV, PV, VD, VI, VN	ACE inhibitor, antiamnestic, antithrombotic, glucose uptake stimulation, regulating the stomach mucosal membrane activity, DPP-IV inhibitor
LFENLQNYR	1196.33	0.29	0.17	Convicilin	LF, NY, LQ, YR, NLQ, NL, QN	ACE inhibitor, antiinflammation, neuropeptide, DPP-IV inhibitor
LPQHIDADL	1021.14	0.32	0.10	Convicilin	DA, PQ, LP, AD, HI, QH, LPQ	ACE inhibitor, α-glucosidase inhibitor, DPP-IV inhibitor
DEEDLRLV	988.06	0.23	0.18	Convicilin	RL, LR, DEE, LV, EE	ACE inhibitor, antithrombotic, stimulating vasoactive substance release, glucose uptake stimulation, DPP-IV inhibitor
FDKRSDLFENL	1383.52	0.53	0.12	Convicilin	LF, KR, FDK, NL	ACE inhibitor, DPP-IV inhibitor
LFENLQ	762.86	0.22	0.13	Convicilin	LF, LQ, NLQ, NL	ACE inhibitor, antiinflammation, DPP-IV inhibitor
AKPHTIFLPQ	1151.37	0.53	0.15	Convicilin	IF, KP, PQ, PH, IFL, LP, PHT, FL, HT, TI, LPQ	ACE inhibitor, antioxidant, DPP-IV inhibitor
LLQRFD	790.92	0.37	0.32	Convicilin	RF, LQ, LL	ACE inhibitor, glucose uptake stimulation, DPP-IV inhibitor
RSDLFENL	993.08	0.48	0.14	Convicilin	LF, NL	ACE inhibitor, DPP-IV inhibitor
SDLFENL	836.90	0.52	0.07	Convicilin	LF, NL	ACE inhibitor, DPP-IV inhibitor
TIDPNGLHLPSFSPSPQ	1806.99	0.34	0.07	Legumin	LHLP, GL, HL, NG, SF, PQ, LP, LH, LHL, SP, PN, PS, TI	ACE inhibitor, antioxidant, DPP-IV inhibitor
FRKGDIIAIPSGIPY	1646.95	0.44	0.07	Legumin	AIP, IA, IP, FR, GI, KG, AI, SG, GD, IAIP, IPY, II, PY, PS	ACE inhibitor, antiinflammation, glucose uptake stimulation, DPP-IV inhibitor

SQIVRVEGGLR	1213.40	0.36	0.28	Legumin	IVR, GL, GG, EG, VR, VE, LR, IV, QI, VE	ACE inhibitor, glucose uptake stimulation, α-glucosidase inhibitor, DPP-IV inhibitor
PNGLHLPSFSPSPQ	1477.64	0.51	0.08	Legumin	LHLP, GL, HL, NG, SF, PQ, LP, LH, LHL, SP, PN, PS, TI	ACE inhibitor, antioxidant, DPP-IV inhibitor
GLHLPSFSPSPQ	1266.42	0.45	0.07	Legumin	LHLP, GL, HL, SF, PQ, LP, LH, LHL, SP, PS	ACE inhibitor, antioxidant, DPP-IV inhibitor
KGDIIAIPSGIPY	1343.59	0.48	0.06	Legumin	AIP, IA, IP, GI, KG, AI, SG, GD, IAIP, IPY, II, PY, PS	ACE inhibitor, antiinflammation, glucose uptake stimulation, DPP-IV inhibitor
ATPSEVLANAFGLR	1445.64	0.53	0.24	Legumin	AF, LA, GL, FG, EV, TP, LR, VL, SE, LAN, AT, NA, PS	ACE inhibitor, antioxidant, stimulating vasoactive substance release, glucose uptake stimulation, DPP-IV inhibitor
DSINALEPDHR	1266.33	0.26	0.09	Legumin	ALEP, ALEPDHR, EP, AL, HR, IN, NA, SI	ACE inhibitor, antioxidant, DPP-IV inhibitor
LKLSGNRGPL	1054.26	0.53	0.45	Legumin	GP, LKL, GPL, PL, SG, KL, RG, LK, NR	ACE inhibitor, antioxidant, antiamnestic, antithrombotic, regulating the stomach mucosal membrane activity, DPP-IV inhibitor
GGNPETEFPETQ	1305.32	0.21	0.07	Legumin	FP, GG, TE, TQ, EF, NP, ET, PE, EF	ACE inhibitor, hypolipidemic, α-glucosidase inhibitor, DPP-IV inhibitor
ISTANSLTLPVLR	1384.64	0.26	0.29	Legumin	ST, LR, LP, VL, SL, TA, LT, PV	ACE inhibitor, glucose uptake stimulation, DPP-IV inhibitor
AAVSHVQQVFR	1241.42	0.21	0.24	Legumin	VF, AA, FR, AV, HV, QQ, QV, SH, VQ, VS	ACE inhibitor, hypotensive, DPP-IV inhibitor
HLPSFSPSPQ	1096.21	0.47	0.06	Legumin	HL, SF, PQ, LP, SP, PS	ACE inhibitor, antioxidant, DPP-IV inhibitor
KGQLVVVPQNFVVA	1497.80	0.26	0.34	Legumin	VP, KG, GQ, NF, PQ, LV, VVV, VA, VP, VV, QL, QN	ACE inhibitor, anticancer, neuropeptide, glucose uptake stimulation, DPP-IV inhibitor
LGGNPETEFPET	1290.35	0.24	0.08	Legumin	FP, GG, LG, TE, EF, NP, ET, PE	ACE inhibitor, hypolipidemic, α-glucosidase inhibitor, DPP-IV inhibitor
KGDIIAIPS	913.08	0.25	0.06	Legumin	AIP, IA, IP, KG, AI, GD, IAIP, II, PS	ACE inhibitor, glucose uptake stimulation, DPP-IV inhibitor
WTYNHGDEPL	1231.29	0.21	0.09	Legumin	PL, HG, GD, YN, TY, EP, WT, NH, YN	ACE inhibitor, antioxidant, DPP-IV inhibitor
GGNPETEFPET	1177.19	0.30	0.07	Legumin	FP, GG, TE, EF, NP, ET, PE	ACE inhibitor, hypolipidemic, α-glucosidase inhibitor, DPP-IV inhibitor

LLLFASACLATSSE	1425.66	0.24	0.15	Legumin	LF, LA, LLF, LLL, LL, SE, FA, AS, AT, TS	ACE inhibitor, stimulating vasoactive substance release, glucose uptake stimulation, DPP-IV inhibitor
ASHKTNEVYFFKGDYYAR	2196.41	0.21	0.12	Albumin	VY, YA, KG, GD, AR, EV, HK, DY, YY, FF, DYY, YYA, VY, AS, EV, KT, NE, SH, TN	ACE inhibitor, antioxidant, DPP-IV inhibitor
LFINDKYVLLDYAPGTSNDK	2286.57	0.20	0.13	Albumin	PG, LF, AP, YA, GT, KY, DY, YV, LDY, YVL, PG, VL, LL, APG, IN, ND, TS, YV	ACE inhibitor, antioxidant, antiamnestic, antithrombotic, glucose uptake stimulation, regulating the stomach mucosal membrane activity, DPP-IV inhibitor
FPFFEGTVFENGIDAAYR	2080.28	0.50	0.08	Albumin	VF, FP, AY, AA, GI, DA, GT, EG, NG, FEGTVFENG, FF, YR, TV	ACE inhibitor, antioxidant, neuropeptide, hypotensive, DPP-IV inhibitor
LYGPTPVRDG	1074.20	0.28	0.19	Albumin	GP, LY, YG, VR, DG, PT, TP, PV	ACE inhibitor, antioxidant, antiinflammation, antiamnestic, antithrombotic, regulating the stomach mucosal membrane activity, DPP-IV inhibitor
KIADMFPFFEGTV	1501.76	0.63	0.07	Albumin	MF, FP, IA, GT, EG, DM, FF, AD, EG, KI, TV	ACE inhibitor, *α*-glucosidase inhibitor, DPP-IV inhibitor
FEGTVFENGIDAA	1369.45	0.23	0.06	Albumin	VF, AA, GI, DA, GT, EG, NG, FEGTVFENG, TV	ACE inhibitor, hypotensive, DPP-IV inhibitor
GKEVYLFKGDQ	1283.45	0.28	0.13	Albumin	VY, YL, LF, KG, GK, GD, EV, KE, DQ, EV	ACE inhibitor, antioxidant, neuropeptide, DPP-IV inhibitor
GKEVYLFKGDQYAR	1673.89	0.32	0.19	Albumin	VY, YL, LF, YA, KG, GK, GD, AR, EV, KE, DQ, EV	ACE inhibitor, antioxidant, neuropeptide, DPP-IV inhibitor
TLDYWPSLR	1150.30	0.65	0.28	Albumin	YW, DY, LR, LDY, SLR, SL, WP, PS, TL	ACE inhibitor, antioxidant, antiinflammation, DPP-IV inhibitor
KIADMFPF	968.18	0.92	0.07	Albumin	MF, FP, IA, DM, AD, KI, PF	ACE inhibitor, *α*-glucosidase inhibitor, DPP-IV inhibitor
SSQNNEAYLFINDKYV	1905.05	0.28	0.08	Albumin	YL, LF, AY, EA, KY, YV, IN, ND, NE, NN, QN	ACE inhibitor, antioxidant, neuropeptide, *α*-glucosidase inhibitor, DPP-IV inhibitor
SSQNNEAYLFINDKYVLL	2131.37	0.56	0.10	Albumin	YL, LF, AY, EA, KY, YV, YVL, VL, LL, IN, ND, NE, NN, QN	ACE inhibitor, antioxidant, antibacterial, neuropeptide, glucose uptake stimulation, *α*-glucosidase inhibitor, DPP-IV inhibitor
TNEVYFFKGDYY	1545.67	0.21	0.06	Albumin	VY, KG, GD, EV, DY, YY, FF, DYY, NE, TN, YF	ACE inhibitor, antioxidant, DPP-IV inhibitor
FPFFEGTV	943.07	0.66	0.06	Albumin	FP, GT, EG, FF, TV	ACE inhibitor, DPP-IV inhibitor
TLDYWPSL	994.11	0.62	0.14	Albumin	YW, DY, LDY, SL, WP, PS, TL	ACE inhibitor, antioxidant, antiinflammation, DPP-IV inhibitor

ENGIDAAYRSTR	1352.43	0.29	0.14	Albumin	AY, AA, GI, DA, NG, ST, YR, NG, TR	ACE inhibitor, hypotensive, antioxidant, neuropeptide, DPP-IV inhibitor
FEGTVFENGIDA	1298.37	0.22	0.06	Albumin	VF, GI, DA, GT, EG, NG, FEGTVFENG, TV	ACE inhibitor, DPP-IV inhibitor
GKEVYLFK	983.18	0.44	0.21	Albumin	VY, YL, LF, GK, EV, KE, EV	ACE inhibitor, antioxidant, neuropeptide, DPP-IV inhibitor
KIADMFPFFEGT	1402.63	0.74	0.07	Albumin	MF, FP, IA, GT, EG, DM, FF, AD, EG, KI	ACE inhibitor, α-glucosidase inhibitor, DPP-IV inhibitor
KTLDYWPSLR	1278.47	0.55	0.37	Albumin	YW, DY, LR, LDY, SLR, SL, WP, KT, PS, TL	ACE inhibitor, antioxidant, antiinflammation, DPP-IV inhibitor
KIADMFP	821.00	0.73	0.08	Albumin	MF, FP, IA, DM, FF, AD, KI	ACE inhibitor, α-glucosidase inhibitor, DPP-IV inhibitor
PFFEGTVF	943.07	0.72	0.06	Albumin	VF, GT, EG, FF, PF, TV	ACE inhibitor, DPP-IV inhibitor
FASHKTNEVYFFKGDYYAR	2343.58	0.23	0.11	Albumin	VY, YA, KG, GD, AR, EV, HK, DY, YY, FF, DYY, YYA, FA, AS, KT, NE, SH, TN	ACE inhibitor, antioxidant, DPP-IV inhibitor
ADMFPF	726.85	0.98	0.05	Albumin	MF, FP, DM, AD	ACE inhibitor, α-glucosidase inhibitor, DPP-IV inhibitor
RGIIPL	667.85	0.59	0.18	Albumin	PL, IP, GI, RG, II	ACE inhibitor, glucose uptake stimulation, DPP-IV inhibitor
ASSNLNLLGFGINAENNQR	2032.20	0.46	0.13	Provicilin	GF, GI, FG, LG, LN, LL, LGF, AE, AS, IN, NA, NL, NN, NQ	ACE inhibitor, antioxidant, glucose uptake stimulation, DPP-IV inhibitor
LTPGDVFVIPAGHPVAVR	1845.17	0.37	0.21	Provicilin	PG, VF, IPA, IP, AG, GH, GD, VR, VAV, HP, AV, TP, VAA, PA, TP, PV	ACE inhibitor, antiamnestic, antithrombotic, regulating the stomach mucosal membrane activity, DPP-IV inhibitor
KNPQLQDLDIFVNY	1706.92	0.39	0.11	Provicilin	IF, NY, LQ, PQ, NP, QD, QL, VN	ACE inhibitor, DPP-IV inhibitor
KNPQLQDLDIFVNYVEIK	2176.50	0.32	0.12	Provicilin	IF, NY, EI, VE, LQ, PQ, YV, YVE, NP, EI, QD, QL, VN	ACE inhibitor, antioxidant, α-glucosidase inhibitor, DPP-IV inhibitor
SSNLNLLGFGINAENNQR	1961.12	0.46	0.12	Provicilin	GF, GI, FG, LG, LN, LL, LGF, AE, IN, NA, NL, NN, NQ	ACE inhibitor, antioxidant, glucose uptake stimulation, DPP-IV inhibitor
KNPQLQDLDIFVNYV	1806.05	0.36	0.12	Provicilin	IF, NY, LQ, PQ, YV, NP, QD, QL, VN	ACE inhibitor, DPP-IV inhibitor
ASSNLNLLGFGINAENNQRN	2146.30	0.51	0.13	Provicilin	GF, GI, FG, LG, LN, LL, LGF, AE, IN, NA, NL, NN, NQ, RN	ACE inhibitor, antioxidant, glucose uptake stimulation, DPP-IV inhibitor
ASSNLNLLG	887.99	0.64	0.16	Convicilin /Provicilin	LG, LN, LL, AS, NL	ACE inhibitor, glucose uptake stimulation, DPP-IV inhibitor

FFEITPK	881.04	0.54	0.07	Provicilin	EI, TP, FF, PK	ACE inhibitor, DPP-IV inhibitor
FFEITPKKNPQLQ	1589.85	0.39	0.15	Provicilin	EI, LQ, PQ, TP, FF, KK, NP, QL	ACE inhibitor, DPP-IV inhibitor
AKLTPGDVFVIPA	1327.59	0.34	0.18	Provicilin	PG, VF, IPA, IP, GD, KL, TP, PA, LT, VI	ACE inhibitor, antithrombotic, regulating the stomach mucosal membrane activity, DPP-IV inhibitor
AKLTPGDVFVIPAGHPV AV	1888.24	0.43	0.21	Provicilin	PG, VF, IPA, IP, AG, GH, GD, VAV, KL, HP, AV, TP, PA, LT, VI	ACE inhibitor, antiamnestic, antithrombotic, regulating the stomach mucosal membrane activity, DPP-IV inhibitor
ASSNLNLL	830.94	0.63	0.18	Convicilin /Provicilin	LN, LL, AS, NL	ACE inhibitor, glucose uptake stimulation, DPP-IV inhibitor
SGSDDNVISQIENPVK	1701.81	0.20	0.06	Convicilin	VK, GS, SG, IE, NP, DN, NV, PV, QI, VI	ACE inhibitor, DPP-IV inhibitor
GDTIKIPAGTTSYL	1436.62	0.30	0.11	Convicilin	IPA, YL, IP, AG, GT, GD, SY, KI, TI, TS, TT	ACE inhibitor, neuropeptide, DPP-IV inhibitor
AIVVLLVNEGKGNLEL	1681.01	0.35	0.26	Convicilin	KG, GK, AI, EG, VVL, VL, LV, IV, LL, EL, VV, EG, NE, NL, VN	ACE inhibitor, antioxidant, glucose uptake stimulation, DPP-IV inhibitor
LSPGDVVIIPAGHPV	1470.73	0.39	0.09	Convicilin	PG, IPA, LSP, IP, AG, GH, GD, HP, II, PA, VV, SP, PV, VI	ACE inhibitor, antiamnestic, antithrombotic, glucose uptake stimulation, regulating the stomach mucosal membrane activity, DPP-IV inhibitor
LSPGDVVIIPAGHPVA	1541.81	0.39	0.10	Convicilin	PG, IPA, LSP, IP, AG, GH, GD, HP, II, PA, VV, SP, PV, VI	ACE inhibitor, antiamnestic, antithrombotic, glucose uptake stimulation, regulating the stomach mucosal membrane activity, DPP-IV inhibitor
AIVVLLVNEGKGNLE	1567.85	0.27	0.23	Convicilin	KG, GK, AI, EG, VVL, VL, LV, IV, LL, EL, VV, EG, NE, NL, VN	ACE inhibitor, glucose uptake stimulation, DPP-IV inhibitor
FVIPVNRPGK	1126.37	0.57	0.22	Convicilin	PG, IP, RP, GK, NR, PV, VI, VN	ACE inhibitor, antithrombotic, regulating the stomach mucosal membrane activity, DPP-IV inhibitor
RVVDFVIPV	1043.27	0.21	0.18	Convicilin	IP, DF, VV, PV, VD, VI, RV	ACE inhibitor, DPP-IV inhibitor
DFVIPVNRPGK	1241.46	0.58	0.16	Convicilin	PG, IP, RP, GK, DF, NR, PV, VI, VN	ACE inhibitor, antiamnestic, antithrombotic, regulating the stomach mucosal membrane activity, DPP-IV inhibitor

ASSNLNLLGFGINAKNNQ	1875.07	0.43	0.14	Convicilin	GF, GI, FG, LG, LN, LL, LGF, AS, IN, NA, NL, NN, NQ	ACE inhibitor, antioxidant, glucose uptake stimulation, DPP-IV inhibitor
VVIIPAGHPVA	1072.32	0.20	0.11	Convicilin	IPA, IP, AG, GH, HP, II, VA, PA, VV, HP, PV, VI	ACE inhibitor, glucose uptake stimulation, DPP-IV inhibitor
NNQRNFLSGSDDNVISQ	1907.97	0.23	0.09	Convicilin	GS, SG, NF, FL, DN, NN, NQ, NV, RN, VI	ACE inhibitor, DPP-IV inhibitor
GDTIKIPAGT	972.11	0.27	0.10	Convicilin	IPA, IP, AG, GT, GD, PA, KI, TI	ACE inhibitor, DPP-IV inhibitor
GDTIKIPAGTT	1073.21	0.24	0.12	Convicilin	IPA, IP, AG, GT, GD, PA, KI, TI	ACE inhibitor, DPP-IV inhibitor
PGDVVIIPAGHPVAIS	1541.81	0.39	0.08	Convicilin	PG, IPA, IP, AG, GH, AI, GD, HP, II, VA, PA, VV, HP, PV, VI	ACE inhibitor, antiamnestic, antithrombotic, glucose uptake stimulation, regulating the stomach mucosal membrane activity, DPP-IV inhibitor
SGSDDNVISQI	1134.16	0.27	0.05	Convicilin	GS, SG, DN, NV, QI, VI	ACE inhibitor, DPP-IV inhibitor
GDTIKIPAG	871.00	0.34	0.09	Convicilin	IPA, IP, AG, GD, PA, KI, TI	ACE inhibitor, DPP-IV inhibitor
KYPQLQDLDI	1232.40	0.21	0.12	Convicilin	YP, KY, LQ, PQ, QD, QL	ACE inhibitor, α-glucosidase inhibitor, DPP-IV inhibitor
KSLPSEFEPF	1180.32	0.56	0.07	Convicilin	FEP, EF, LP, SE, SL, KS, PF, PS	ACE inhibitor, hypolipidemic, stimulating vasoactive substance release, DPP-IV inhibitor
LMLPH	609.78	0.58	0.24	Convicilin	PH, LP, LM, ML	ACE inhibitor, DPP-IV inhibitor
VVIIPAGH	804.99	0.21	0.08	Convicilin	IPA, IP, AG, GH, II, PA, VV, VI	ACE inhibitor, glucose uptake stimulation, DPP-IV inhibitor
GFGINAKNNQRN	1332.44	0.32	0.17	Convicilin	GF, GI, FG, IN, NA, NN, NQ, RN	ACE inhibitor, DPP-IV inhibitor
VIIPAGHPVA	973.18	0.23	0.10	Convicilin	IPA, IP, AG, GH, HP, II, VA, PA, VV, PV, VI	ACE inhibitor, glucose uptake stimulation, DPP-IV inhibitor
VIIPAGHPV	902.10	0.23	0.08	Convicilin	IPA, IP, AG, GH, HP, II, VA, PA, VV, PV, VI	ACE inhibitor, glucose uptake stimulation, DPP-IV inhibitor
ALMLPH	680.86	0.62	0.26	Convicilin	PH, LP, AL, LM, ML	ACE inhibitor, DPP-IV inhibitor
FVIPVNRPG	998.19	0.54	0.14	Convicilin	PG, IP, RP, NR, PV, VI, VN	ACE inhibitor, antiamnestic, antithrombotic, regulating the stomach mucosal membrane activity, DPP-IV inhibitor
ASSNLNLLGFGINAKNNQR	2031.26	0.56	0.18	Convicilin	GF, GI, FG, LG, LN, LL, LGF, AS, IN, LN, NA, NL, NN, NQ	ACE inhibitor, antioxidant, glucose uptake stimulation, DPP-IV inhibitor

SPGDVVIIPAGHPVAI	1541.81	0.53	0.08	Convicilin	PG, IPA, IP, AG, GH, AI, GD, HP, II, VA, PA, VV, HP, PV, VI	ACE inhibitor, antiamnestic, antithrombotic, glucose uptake stimulation, regulating the stomach mucosal membrane activity, DPP-IV inhibitor
LSPGDVVIIPAGH	1274.48	0.45	0.08	Convicilin	PG, IPA, LSP, IP, AG, GH, AI, GD, HP, II, VA, PA, VV, HP, PV, VI	ACE inhibitor, antiamnestic, antithrombotic, glucose uptake stimulation, regulating the stomach mucosal membrane activity, DPP-IV inhibitor
KSLPSEFEP	1033.15	0.27	0.08	Convicilin	FEP, EF, LP, SE, SL, EP, KS, PS	ACE inhibitor, hypolipidemic, stimulating vasoactive substance release, DPP-IV inhibitor
LMLPHYNS	974.14	0.22	0.09	Convicilin	HY, PH, YN, LP, PHY, LM, ML	ACE inhibitor, antioxidant, antiinflammation, DPP-IV inhibitor
LSPGDVVIIPAGHPVAIS	1742.05	0.36	0.08	Convicilin	PG, IPA, LSP, IP, AG, GH, AI, GD, HP, II, VA, PA, VV, HP, PV, VI	ACE inhibitor, antiamnestic, antithrombotic, glucose uptake stimulation, regulating the stomach mucosal membrane activity, DPP-IV inhibitor
GALMLPH	737.92	0.66	0.21	Convicilin	GA, PH, LP, AL, LM, ML	ACE inhibitor, DPP-IV inhibitor
IKIPAGT	698.86	0.20	0.13	Convicilin	IPA, IP, AG, FT, PA, KI	ACE inhibitor, DPP-IV inhibitor
INKGALMLPH	1093.35	0.38	0.19	Convicilin	GA, KG, NK, PH, LP, AL, IN, LM, ML	ACE inhibitor, DPP-IV inhibitor
IRENIADAAGADLYNPR	1859.03	0.32	0.16	Legumin	IR, LY, PR, IA, AA, GA, AG, DA, YN, NPR, AD	ACE inhibitor, antioxidant, hypotensive, α-glucosidase inhibitor, DPP-IV inhibitor
IRENIADAAGAD	1215.29	0.62	0.11	Legumin	IR, IA, AA, GA, AG, DA, YN, NPR, AD	ACE inhibitor, antioxidant, hypotensive, α-glucosidase inhibitor, DPP-IV inhibitor
ATPAEVLANAFGL	1273.45	0.58	0.22	Legumin	AF, LA, GL, FG, EV, TP, VL, LAN, PA, AE, AT, EF, NA	ACE inhibitor, antioxidant, glucose uptake stimulation, DPP-IV inhibitor
LKLSGNRGPLVHPQ	1515.78	0.28	0.34	Legumin	GP, LKL, GPL, PL, SG, KL, PQ, HP, RG, LV, LK, NR, VH	ACE inhibitor, antioxidant, antiamnestic, antithrombotic, glucose uptake stimulation, regulating the stomach mucosal membrane activity, DPP-IV inhibitor
IVNFQGDAVFDNK	1466.61	0.22	0.09	Legumin	VF, DA, QG, GD, NF, NK, AV, FQ, IV, DN, QG, VN, DA	ACE inhibitor, glucose uptake stimulation, DPP-IV inhibitor

ATPAEVLANAFGLRQR	1713.96	0.48	0.48	Legumin	AF, LA, GL, FG, EV, TP, VL, LAN, PA, AE, AT, EF, NA	ACE inhibitor, antioxidant, glucose uptake stimulation, DPP-IV inhibitor
ATPAEVLANAF	1103.24	0.26	0.21	Legumin	AF, LA, EV, TP, VL, LAN, PA, AE, AT, NA	ACE inhibitor, antioxidant, glucose uptake stimulation, DPP-IV inhibitor
GLTETWNPNNPELK	1612.76	0.22	0.24	Legumin	GL, TE, EL, PEL, LK, TW, NP, WN, ET, LT, NN, PN, PE	ACE inhibitor, antioxidant, α-glucosidase inhibitor, DPP-IV inhibitor
TETWNPNNPEL	1314.37	0.21	0.16	Legumin	TE, EL, PEL, LK, TW, NP, WN, ET, LT, NN, PN, PE	ACE inhibitor, antioxidant, α-glucosidase inhibitor, DPP-IV inhibitor
DNINALEPDHR	1293.36	0.21	0.10	Legumin	ALEP, ALEPDHR, EP, AL, DN, HR, IN, NA	ACE inhibitor, antioxidant, DPP-IV inhibitor
SEAGLTETWNPNNPEL	1771.86	0.34	0.13	Legumin	GL, AG, EA, TE, SE, EL, PEL, TW, NP, WN, ET, LT, NN, PN, PE	ACE inhibitor, antioxidant, stimulating vasoactive substance release, α-glucosidase inhibitor, DPP-IV inhibitor
VESEAGLTETWNPNNPEL	2000.11	0.23	0.13	Legumin	GL, AG, EA, VE, TE, SE, EL, PEL, TW, NP, WN, ET, LT, NN, PN, PE	ACE inhibitor, antioxidant, stimulating vasoactive substance release, α-glucosidase inhibitor, DPP-IV inhibitor
GLTETWNPNNPEL	1484.59	0.26	0.18	Legumin	GL, TE, EL, PEL, TW, NP, WN, ET, LT, NN, PN, PE	ACE inhibitor, antioxidant, α-glucosidase inhibitor, DPP-IV inhibitor
AGLTETWNPNNPEL	1555.66	0.28	0.19	Legumin	GL, AG, TE, EL, PEL, TW, NP, WN, ET, LT, NN, PN, PE	ACE inhibitor, antioxidant, α-glucosidase inhibitor, DPP-IV inhibitor
AGLTETWNPNNPELK	1683.84	0.24	0.26	Legumin	GL, AG, TE, EL, PEL, LK, TW, NP, WN, ET, LT, NN, PN, PE	ACE inhibitor, antioxidant, α-glucosidase inhibitor, DPP-IV inhibitor
RSPEDDRKQIVK	1470.65	0.21	0.26	Glycinin	VK, DR, IV, SP, QI, RK, PE	ACE inhibitor, glucose uptake stimulation, α-glucosidase inhibitor, DPP-IV inhibitor
DLAGSSQADVFNPR	1476.57	0.41	0.12	Legumin	VF, PR, LA, AG, GS, AGSS, AGS, ADVFNPR, NPR, NP, AD, FN, QA	ACE inhibitor, antioxidant, hypotensive, α-glucosidase inhibitor, DPP-IV inhibitor
HEDLAGSSQADVFNPR	1742.82	0.30	0.10	Legumin	VF, PR, LA, AG, GS, AGSS, AGS, ADVFNPR, NPR, NP, AD, FN, QA	ACE inhibitor, antioxidant, hypotensive, α-glucosidase inhibitor, DPP-IV inhibitor

EDLAGSSQADVFNPR	1605.68	0.32	0.11	Legumin	VF, PR, LA, AG, GS, AGSS, AGS, ADVFNPR, NPR, NP, AD, FN, QA	ACE inhibitor, antioxidant, hypotensive, α-glucosidase inhibitor, DPP-IV inhibitor
LHEDLAGSSQADVFNPR	1855.98	0.36	0.12	Legumin	VF, PR, LA, AG, GS, AGSS, AGS, ADVFNPR, NPR, NP, AD, FN, QA	ACE inhibitor, antioxidant, hypotensive, α-glucosidase inhibitor, DPP-IV inhibitor
WNLNAN	730.78	0.41	0.26	Legumin	LN, WN, NA, NL	ACE inhibitor, DPP-IV inhibitor
ITSVNSLTLPVLKLLHLSAQ	2147.59	0.20	0.29	Legumin	LKL, HL, KL, LP, VL, LL, SL, LH, LHL, LK, LT, PV, SV, TL, TS, VN	ACE inhibitor, antioxidant, glucose uptake stimulation, DPP-IV inhibitor
KNGIFMPH	943.13	0.74	0.08	Legumin	IF, GI, NG, PH, MP, FM	ACE inhibitor, DPP-IV inhibitor
FREGDLIAVPT	1217.39	0.24	0.14	Glycinin	AVP, IA, VP, FR, GD, EG, PT, AV, LI	ACE inhibitor, glucose uptake stimulation, DPP-IV inhibitor
VLIVPQNFAVAA	1241.50	0.28	0.19	Glycinin	VAA, VLIVP, VP, AA, NF, PQ, AV, VL, IV, LI, FA, VP, QN	ACE inhibitor, hypotensive, glucose uptake stimulation, DPP-IV inhibitor
PQTFEEPTQRG	1289.37	0.21	0.15	Glycinin	PT, TQ, PQ, TF, RG, EE, TFE, EP, QT, TF	ACE inhibitor, antioxidant, stimulating vasoactive substance release, DPP-IV inhibitor
QQGSGIFGIIFSGCPQ	1638.86	0.78	0.05	Glycinin	IF, GI, FG, GS, QG, SG, IFG, PQ, II, QQ	ACE inhibitor, glucose uptake stimulation, DPP-IV inhibitor
SLNQTIFGS	966.06	0.28	0.06	Albumin	IF, FG, GS, IFG, LN, SL, NQ, QT, TI	ACE inhibitor, DPP-IV inhibitor

[a]From PeptideRanker. [b]From CPPpred. [c]From ExPASy ProtParam. [d]From BIOPEP.

Table S4. Potential bioactive peptides in ByPP-rice protein.

Peptide sequence	MW	PeptideRanker score[a]	CPPpred score[b]	Parent protein	Potential bioactive peptides[d]	Biological function[d]
QNIDNPNRADTYNPR	1787.87	0.25	0.16	Glutelin/Globulin	PR, RA, YN, NPR, TY, NP, AD, DN, NR, PN, QN	ACE inhibitor, antioxidant, α-glucosidase inhibitor, DPP-IV inhibitor
GLLLPHYTNGASLVYIIQGR	2185.55	0.39	0.15	Glutelin/Globulin	VY, HY, LLP, GA, GL, GR, QG, NG, PH, LVY, ASL, LP, LLL, LV, II, LL, SL, LLPH, PHY, YT, AS, IQ, TN, YI	ACE inhibitor, antioxidant, antiinflammation, stimulating vasoactive substance release, glucose uptake stimulation, DPP-IV inhibitor
QGDVIALPAGVAHW	1433.63	0.66	0.17	Glutelin/Globulin	IA, AG, GV, QG, GD, AH, ALH, LPAGV, LP, VA, PA, AL, HW, VI	ACE inhibitor, antioxidant, DPP-IV inhibitor
GLLLPHYTNGASLV	1454.69	0.39	0.19	Glutelin/Globulin	HY, LLP, GA, GL, NG, PH, ASL, LP, LLL, LV, LL, SL, LLPH, PHY, YT, AS, TN	ACE inhibitor, antioxidant, antiinflammation, stimulating vasoactive substance release, glucose uptake stimulation, DPP-IV inhibitor
GQLLIIPQ	881.08	0.33	0.12	Glutelin/Globulin	IP, GQ, PQ, LI, II, LL, QL	ACE inhibitor, neuropeptide, glucose uptake stimulation, DPP-IV inhibitor
GQLLIIPQH	1018.22	0.32	0.10	Glutelin/Globulin	IP, GQ, PQ, LIIPQH, LI, LL, II, QH, QL	ACE inhibitor, neuropeptide, glucose uptake stimulation, DPP-IV inhibitor
FDRLQAFEPI	1235.41	0.41	0.11	Glutelin/Globulin	RL, AF, LQ, FEP, DR, EP, PI, QA	ACE inhibitor, DPP-IV inhibitor
NTQNFPIL	946.07	0.54	0.10	Glutelin	FP, NF, TQ, IL, NT, PI, QN	ACE inhibitor, glucose uptake stimulation, DPP-IV inhibitor
DFLLAGNK	877.01	0.36	0.21	Glutelin/Globulin	LA, AG, NK, DF, LL, FL	ACE inhibitor, glucose uptake stimulation, DPP-IV inhibitor
LQAFEPIRSV	1159.35	0.32	0.13	Glutelin/Globulin	IR, AF, LQ, FEP, EP, PI, QA, SV	ACE inhibitor, antioxidant, DPP-IV inhibitor
NTQNFPILN	1060.17	0.39	0.10	Glutelin	FP, NF, LN, TQ, IL, NT, PI, QN, TQ	ACE inhibitor, glucose uptake stimulation, DPP-IV inhibitor
GLLLPHYTNGASL	1355.56	0.51	0.17	Glutelin/Globulin	HY, LLP, GA, GL, NG, PH, ASL, LP, LLL, LL, SL, LLPH, PHY, YT, AS, TN	ACE inhibitor, antioxidant, antiinflammation, stimulating vasoactive substance release, glucose uptake stimulation, DPP-IV inhibitor

QGDVIALPA	883.01	0.35	0.13	Glutelin/Globulin	IA, QG, GD, ALP, LP, PA, AL, VI	ACE inhibitor, DPP-IV inhibitor
QGDVIALPAGV	1039.20	0.47	0.14	Glutelin/Globulin	IA, AG, GV, QG, GD, ALP, LPAGV, LP, PA, AL, VI	ACE inhibitor, antioxidant, DPP-IV inhibitor
LIIPQH	719.88	0.24	0.08	Glutelin/Globulin	IP, PQ, LIIPQH, LI, II, QH	ACE inhibitor, glucose uptake stimulation, DPP-IV inhibitor
RVIQPQGLLVPR	1375.68	0.33	0.54	Glutelin/Globulin	PR, VP, GL, QG, PQ, IQP, QP, LV, LL, IQ, VI	ACE inhibitor, glucose uptake stimulation, DPP-IV inhibitor
TVFDGVLRPGQL	1301.51	0.25	0.30	Glutelin/Globulin	PG, VF, LRP, RP, GV, GQ, PG, DG, LR, VL, QL, TV	ACE inhibitor, antiamnestic, neuropeptide, antithrombotic, glucose uptake stimulation, DPP-IV inhibitor
DGVLRPGQL	954.09	0.36	0.34	Glutelin/Globulin	PG, LRP, RP, GV, GQ, DG, LR, VL, QL	ACE inhibitor, antiamnestic, neuropeptide, antithrombotic, glucose uptake stimulation, regulating the stomach mucosal membrane activity, DPP-IV inhibitor
QQQYYPGLSN	1197.27	0.38	0.08	Glutelin/Globulin	PG, YP, PGL, GL, YPG, YY, YYP, YYPG, QYY, GL, QQ, QY	ACE inhibitor, antioxidant, antiamnestic, opioid, antithrombotic, regulating the stomach mucosal membrane activity, α-glucosidase inhibitor, DPP-IV inhibitor
QGDIVALPAGVAHW	1433.63	0.60	0.17	Glutelin/Globulin	AG, GV, QG, GD, AH, ALP, LPAGV, LP, IV, VA, PA, AL, HW	ACE inhibitor, antioxidant, glucose uptake stimulation, DPP-IV inhibitor
TVFDGVLRPGQLL	1414.67	0.35	0.35	Glutelin/Globulin	PG, VF, LRP, RP, GV, GQ, DG, LR, VL, LL, QL, TV	ACE inhibitor, antiamnestic, neuropeptide, antithrombotic, glucose uptake stimulation, regulating the stomach mucosal membrane activity, DPP-IV inhibitor
VNIENPSRADSYNPR	1731.84	0.21	0.12	Glutelin/Globulin	PR, RA, SY, IE, YN, NPR, NP, AD, PS, VN	ACE inhibitor, α-glucosidase inhibitor, DPP-IV inhibitor
SRVQVVSNFGK	1220.39	0.25	0.22	Glutelin/Globulin	FGK, FG, GK, NF, VQV, VV, QV, VQ, VS	ACE inhibitor, DPP-IV inhibitor

GEIIHVKNGLQ	1207.40	0.21	0.11	Glutelin/Globulin	VK, GL, GE, NG, EI, LQ, II, HV, IH, II	ACE inhibitor, glucose uptake stimulation, DPP-IV inhibitor
ITSVNSQKFPILN	1460.69	0.20	0.09	Glutelin/Globulin	FP, QK, KF, LN, IL, PI, SV, TS, VN	ACE inhibitor, glucose uptake stimulation, DPP-IV inhibitor
VIQPQGLLVPR	1219.49	0.42	0.37	Glutelin/Globulin	PR, VP, GL, QG, PQ, IQP, QP, LV, LL	ACE inhibitor, glucose uptake stimulation, DPP-IV inhibitor
FDRLQAFEPL	1235.41	0.51	0.19	Glutelin/Globulin	RL, PL, AF, LQ, FEP, DR, EP, QA	ACE inhibitor, DPP-IV inhibitor
DGVLRPGQLLIIPQ	1518.82	0.41	0.20	Glutelin/Globulin	PG, LRP, IP, RP, GV, GQ, DG, PQ, LR, VL, LI, II, LL, IP, QL	ACE inhibitor, antiamnestic, neuropeptide, antithrombotic, glucose uptake stimulation, regulating the stomach mucosal membrane activity, DPP-IV inhibitor
FQQQYYPGLS	1230.34	0.56	0.07	Glutelin/Globulin	PG, YP, PGL, GL, YPG, FQ, YY, YYP, YYPG, QYY, QQ, QY	ACE inhibitor, antioxidant, antiamnestic, opioid, antithrombotic, regulating the stomach mucosal membrane activity, α-glucosidase inhibitor, DPP-IV inhibitor
DGVLRPGQLL	1067.25	0.49	0.41	Glutelin/Globulin	FG, LRP, RP, GV, GQ, PG, DG, LR, VL, LL, QL	ACE inhibitor, antiamnestic, neuropeptide, antithrombotic, glucose uptake stimulation, regulating the stomach mucosal membrane activity, DPP-IV inhibitor
FQQQYYPGLSN	1344.45	0.46	0.07	Glutelin/Globulin	PG, YP, PGL, GL, YPG, FQ, YY, YYP, YYPG, QYY, QQ, QY	ACE inhibitor, antioxidant, antiamnestic, opioid, antithrombotic, regulating the stomach mucosal membrane activity, α-glucosidase inhibitor, DPP-IV inhibitor
NGLQLLKPTLT	1197.44	0.34	0.56	Glutelin/Globulin	LKP, GL, NG, KP, LQ, PT, LQL, LL, LKPT, KPT, LK, LT, QL, TL	ACE inhibitor, antioxidant, neuropeptide, glucose uptake stimulation, stimulating GLP-1 release, DPP-IV inhibitor
RQGDIVALPA	1039.20	0.23	0.23	Glutelin/Globulin	QG, GD, ALP, LP, IV, VA, PA, AL	ACE inhibitor, glucose uptake stimulation, DPP-IV inhibitor
PRYTNIPGVV	1115.30	0.27	0.18	Glutelin/Globulin	PG, RY, PRY, PR, IP, GV, VV, YT, TN	ACE inhibitor, antioxidant, antiamnestic, antithrombotic, regulating the stomach mucosal membrane activity, DPP-IV inhibitor

TVFDGVLRPG	1060.22	0.29	0.24	Glutelin/Globulin	PG, VF, LRP, RP, GV, DG, LR, VL, TV	ACE inhibitor, antiamnestic, antithrombotic, glucose uptake stimulation, regulating the stomach mucosal membrane activity, DPP-IV inhibitor
QGDIVALPAGV	1039.20	0.38	0.14	Glutelin/Globulin	AG, GV, QG, GD, ALP, LPAGV, LP, IV, VA, PA, AL	ACE inhibitor, antioxidant, glucose uptake stimulation, DPP-IV inhibitor
GQLLIVPQ	867.06	0.24	0.19	Glutelin/Globulin	VP, GQ, PQ, IV, LI, LL, QL	ACE inhibitor, neuropeptide, glucose uptake stimulation, DPP-IV inhibitor
LNIENPSHADTYNPR	1740.85	0.21	0.10	Glutelin/Globulin	PR, IE, LN, YN, NPR, TY, HA, NP, AD, PS, SH	ACE inhibitor, antioxidant, α-glucosidase inhibitor, DPP-IV inhibitor
LYVFDLNNNANQLEPR	1920.11	0.25	0.17	Glutelin/Globulin	LY, VF, PR, LN, YV, LY, EP, LN, NA, NN, NQ, QL	ACE inhibitor, antioxidant, DPP-IV inhibitor
VFDLNNNANQLEPR	1643.78	0.24	0.18	Glutelin/Globulin	VF, PR, LN, EP, NA, NN, NQ, QL	ACE inhibitor, DPP-IV inhibitor
NIENPSHADTYNPR	1627.69	0.22	0.09	Glutelin/Globulin	PR, IE, YN, NPR, TY, HA, NP, AD, PS, SH	ACE inhibitor, antioxidant, α-glucosidase inhibitor, DPP-IV inhibitor
YVFDLNNNANQLEPR	1806.95	0.27	0.15	Glutelin/Globulin	VF, PR, LN, YV, EP, NA, NN, NQ, QL	ACE inhibitor, DPP-IV inhibitor
TVFNGVLRPGQL	1300.52	0.21	0.36	Glutelin/Globulin	PG, VF, LRP, RP, GV, GQ, NG, LR, VL, FN, QL, TV	ACE inhibitor, antiamnestic, neuropeptide, antithrombotic, glucose uptake stimulation, regulating the stomach mucosal membrane activity, DPP-IV inhibitor
GEELGAFTPR	1076.17	0.37	0.15	Glutelin/Globulin	PR, AF, GA, GE, LG, TP, EE, EL	ACE inhibitor, antioxidant, stimulating vasoactive substance release, DPP-IV inhibitor
NGVLRPGQL	953.11	0.29	0.45	Glutelin/Globulin	PG, LRP, RP, GV, GQ, NG, LR, VL, GQ, QL	ACE inhibitor, antiamnestic, neuropeptide, antithrombotic, glucose uptake stimulation, regulating the stomach mucosal membrane activity, DPP-IV inhibitor

IENPSHADTYNPR	1513.59	0.27	0.08	Glutelin/Globulin	PR, IE, YN, NPR, TY, HA, NP, AD, PS, SH	ACE inhibitor, antioxidant, α-glucosidase inhibitor, DPP-IV inhibitor
SLKNNRGEELGAFTPR	1788.98	0.27	0.25	Glutelin/Globulin	PR, AF, GA, GE, LG, TP, RG, EE, SL, EL, LK, NN, NR	ACE inhibitor, antioxidant, stimulating vasoactive substance release, DPP-IV inhibitor
TVFNGVLRPGQLL	1413.68	0.29	0.42	Glutelin/Globulin	PG, VF, LRP, RP, GV, GQ, NG, LR, VL, LL, FN, QL, TV	ACE inhibitor, antiamnestic, neuropeptide, antithrombotic, glucose uptake stimulation, regulating the stomach mucosal membrane activity, DPP-IV inhibitor
YQQQTYPGFSN	1332.39	0.25	0.07	Glutelin/Globulin	PG, YP, GF, YPG, TY, QQ, QT, YQ	ACE inhibitor, antioxidant, antiamnestic, antithrombotic, regulating the stomach mucosal membrane activity, α-glucosidase inhibitor, DPP-IV inhibitor
NGVLRPGQLLIIPQ	1517.83	0.32	0.25	Glutelin/Globulin	PG, LRP, IP, RP, GV, GQ, NG, PQ, LR, VL, LI, II, LL, QL, VL	ACE inhibitor, antiamnestic, neuropeptide, antithrombotic, glucose uptake stimulation, regulating the stomach mucosal membrane activity, DPP-IV inhibitor
FDLNNNANQLEPR	1544.64	0.24	0.16	Glutelin/Globulin	PR, LN, EP, NA, NN, NQ, QL	ACE inhibitor, DPP-IV inhibitor
NGVLRPGQLL	1066.27	0.41	0.51	Glutelin/Globulin	PG, LRP, RP, GV, GQ, NG, LR, VL, LL, QL, VL	ACE inhibitor, antiamnestic, neuropeptide, antithrombotic, glucose uptake stimulation, regulating the stomach mucosal membrane activity, DPP-IV inhibitor
TVFNGVLRPG	1059.23	0.24	0.31	Glutelin/Globulin	PG, VF, LRP, RP, GV, NG, LR, VL, FN, TV	ACE inhibitor, antiamnestic, antithrombotic, glucose uptake stimulation, regulating the stomach mucosal membrane activity, DPP-IV inhibitor
QFLPEGQ	817.90	0.32	0.09	Glutelin/Globulin	GQ, EG, LP, FLPE, FL, QF, PE	ACE inhibitor, antioxidant, neuropeptide, α-glucosidase inhibitor, DPP-IV inhibitor
LYVFDINNNANQLEPR	1920.11	0.20	0.13	Glutelin	LY, VF, PR, YV, EP, IN, NA, NN, NQ, QL	ACE inhibitor, antioxidant, DPP-IV inhibitor
VFDINNNANQLEPR	1643.78	0.24	0.13	Glutelin	VF, PR, EP, IN, NA, NN, NQ, QL	ACE inhibitor, DPP-IV inhibitor
YVFDINNNANQLEPR	1806.95	0.23	0.12	Glutelin	VF, PR, YV, EP, IN, NA, NN, NQ, QL	ACE inhibitor, DPP-IV inhibitor

FDINNNANQLEPR	1544.64	0.25	0.12	Glutelin	PR, EP, IN, NA, NN, NQ, QL	ACE inhibitor, DPP-IV inhibitor
GLLVPRYSNTPGLV	1485.75	0.38	0.28	Glutelin/Globulin	PG, RY, PRY, PR, PGL, VP, GL, TP, LV, LL, NT, YS	ACE inhibitor, antioxidant, antiamnestic, antithrombotic, glucose uptake stimulation, regulating the stomach mucosal membrane activity, DPP-IV inhibitor
RQNIDNPNLADTYNPR	1901.03	0.22	0.18	Glutelin/Globulin	PR, LA, YN, NPR, TY, NP, AD, DN, NL, PN, QN	ACE inhibitor, antioxidant, α-glucosidase inhibitor, DPP-IV inhibitor
QNIDNPNLADTYNPR	1744.84	0.30	0.13	Glutelin/Globulin	PR, LA, YN, NPR, TY, NP, AD, DN, NL, PN, QN	ACE inhibitor, antioxidant, α-glucosidase inhibitor, DPP-IV inhibitor
NIDNPNLADTYNPR	1616.71	0.24	0.12	Glutelin/Globulin	PR, LA, YN, NPR, TY, NP, AD, DN, NL, PN, QN	ACE inhibitor, antioxidant, α-glucosidase inhibitor, DPP-IV inhibitor
IKFPIV	715.93	0.39	0.09	Glutelin/Globulin	FP, KF, IV, PI	ACE inhibitor, glucose uptake stimulation, DPP-IV inhibitor
ITYLNGQKFPI	1293.53	0.20	0.10	Glutelin/Globulin	FP, YL, GQ, NG, QK, KF, LN, TY, PI	ACE inhibitor, antioxidant, neuropeptide, DPP-IV inhibitor
GLLLPHYSNGATLV	1454.69	0.39	0.18	Glutelin/Globulin	HY, LLP, GA, GL, NG, PH, LP, LLL, LV, LL, LLPH, PHY, AT, TL, YS	ACE inhibitor, antioxidant, antiinflammation, stimulating vasoactive substance release, glucose uptake stimulation, DPP-IV inhibitor
NGQKFPI	802.93	0.55	0.09	Glutelin/Globulin	FP, GQ, NG, QK, KF, PI	ACE inhibitor, neuropeptide, DPP-IV inhibitor
LQAFEPIRT	1074.24	0.49	0.17	Glutelin/Globulin	IR, AF, LQ, FEP, EP, PI, QA	ACE inhibitor, antioxidant, DPP-IV inhibitor
IGQQLYRYE	1169.30	0.26	0.11	Glutelin/Globulin	RY, LY, IG, GQ, YE, YR, YRY, QL, QQ	ACE inhibitor, antioxidant, neuropeptide, DPP-IV inhibitor
IYNSANQLDPR	1290.40	0.30	0.11	Glutelin/Globulin	IY, PR, YN, DP, NQ, QL	ACE inhibitor, antioxidant, DPP-IV inhibitor
GLLLPHYSNGATLVYIIQGR	2185.55	0.31	0.15	Glutelin	VY, HY, LLP, GA, GL, GR, QG, NG, PH, LVY, LP, LLL, LV, II, LL, LLPH, PHY, AT, TL, YS	ACE inhibitor, antioxidant, antiinflammation, stimulating vasoactive substance release, glucose uptake stimulation, DPP-IV inhibitor

GEEIGAFTPR	1076.17	0.37	0.09	Glutelin/Globulin	PR, AF, IG, GA, GE, EI, TP, EE	ACE inhibitor, stimulating vasoactive substance release, DPP-IV inhibitor
NIENPNHADTYNPR	1654.72	0.20	0.10	Glutelin/Globulin	PR, IE, YN, NPR, TY, AD, HA, NP, NH, PN	ACE inhibitor, antioxidant, α-glucosidase inhibitor, DPP-IV inhibitor
SLKNNRGEEIGAFTPR	1788.98	0.24	0.18	Glutelin/Globulin	PR, AF, IG, GA, GE, EI, TP, RG, EE, SL, LK, NN, NR	ACE inhibitor, antioxidant, stimulating vasoactive substance release, DPP-IV inhibitor
VFDVNNNANQLEPR	1629.75	0.21	0.17	Glutelin/Globulin	VF, PR, EP, NA, NN, NQ, QL, VF, VN	ACE inhibitor, DPP-IV inhibitor
EFFLAGKPR	1064.25	0.57	0.24	Glutelin/Globulin	GKP, PR, FFL, LA, AG, GK, KP, EF, FF	ACE inhibitor, antioxidant, hypolipidemic, DPP-IV inhibitor
KNIDNPQSSDIFNPH	1725.83	0.31	0.06	Glutelin/Globulin	IF, PQ, PH, NP, DN, FN, QS	ACE inhibitor, DPP-IV inhibitor
NIDNPQSSDIFNPHGGR	1867.95	0.41	0.06	Glutelin/Globulin	IF, GR, HG, GG, PQ, PH, PHG, NP, DN, FN, QS	ACE inhibitor, antioxidant, DPP-IV inhibitor
KNIDNPQSSDIFNPHGGR	1996.13	0.38	0.07	Glutelin/Globulin	IF, GR, HG, GG, PQ, PH, PHG, NP, DN, FN, QS	ACE inhibitor, antioxidant, DPP-IV inhibitor
SVFDGELH	902.96	0.34	0.06	Glutelin/Globulin	VF, GE, DG, LH, EL, SV	ACE inhibitor, antioxidant, DPP-IV inhibitor
SFGGSPLQSPR	1132.24	0.63	0.10	Glutelin/Globulin	PR, PL, FG, GS, GG, SF, LQ, FGG, SP, QS	ACE inhibitor, DPP-IV inhibitor
ITRANSQNFPILN	1487.68	0.24	0.13	Glutelin/Globulin	FP, RA, NF, LN, IL, PI, QN, TR	ACE inhibitor, glucose uptake stimulation, DPP-IV inhibitor
FQVSMAQFSFGGSPLQSP	1915.15	0.45	0.08	Glutelin/Globulin	PL, FG, GS, GG, SF, LQ, FQ, FGG, MA, SP, QF, QS, QV, VS	ACE inhibitor, DPP-IV inhibitor
NQNIFAGFNPDL	1349.47	0.60	0.07	Globulin	GF, IF, AG, FA, NP, FN, NQ, QN	ACE inhibitor, DPP-IV inhibitor
FRQGDIVALPT	1216.40	0.31	0.18	Globulin	FR, QG, GD, PT, ALP, LP, IV, VA, AL	ACE inhibitor, glucose uptake stimulation, DPP-IV inhibitor

SIQQHFGQNI	1171.28	0.33	0.06	Globulin	FG, GQ, HF, IQ, QH, QN, QQ, SI	ACE inhibitor, neuropeptide, DPP-IV inhibitor
YGEQQQQPGMT	1266.35	0.23	0.10	Globulin	PG, YG, GM, GE, QP, QQ	ACE inhibitor, antiinflammation, antiamnestic, antithrombotic, regulating the stomach mucosal membrane activity, DPP-IV inhibitor
YGEQQQQPGM	1165.24	0.20	0.08	Globulin	PG, YG, GM, GE, QP, QQ	ACE inhibitor, antiinflammation, antiamnestic, antithrombotic, regulating the stomach mucosal membrane activity, DPP-IV inhibitor
ADEIGAFTPR	1076.17	0.48	0.11	Glutelin	PR, AF, IG, GA, EI, TP, AD	ACE inhibitor, *α*-glucosidase inhibitor, DPP-IV inhibitor
SFANQLEPR	1061.16	0.56	0.15	Glutelin/Globulin	PR, SF, FA, EP, NQ, QL	ACE inhibitor, DPP-IV inhibitor
YGIYPR	767.88	0.70	0.09	Prolamin	IYP, IY, YPR, PR, IYPR, YG, YP, GI	ACE inhibitor, antioxidant, antiinflammation, *α*-glucosidase inhibitor, DPP-IV inhibitor
FRIPE	660.77	0.51	0.10	Seed storage protein	IP, FR, RI, PE	ACE inhibitor, *α*-glucosidase inhibitor, DPP-IV inhibitor
FQQGDVIAVPA	1144.29	0.26	0.10	Globulin	AVP, IA, VP, QG, GD, AV, FQ, PA, QQ, VI	ACE inhibitor, DPP-IV inhibitor

[a]From PeptideRanker. [b]From CPPpred. [c]From ExPASy ProtParam. [d]From BIOPEP.

Table S5. Potential bioactive peptides in ByPP-mungbean protein.

Peptide sequence	MW	PeptideRanker score[a]	CPPpred score[b]	Parent protein	Potential bioactive peptides[d]	Biological function[d]
GQESQQEGVIVELKR	1699.88	0.21	0.17	Globulin	KR, GV, GQ, EG, VE, EL, LK, IV, ES, QE, QQ, VI	ACE inhibitor, antioxidant, neuropeptide, glucose uptake stimulation, α-glucosidase inhibitor, DPP-IV inhibitor
FLSSTEAQQSYLQGFSK	1921.09	0.21	0.10	Globulin	YL, GF, QG, EA, SY, TE, LQ, ST, FL, QQ, QS, SK	ACE inhibitor, neuropeptide (anxiolytic peptide), α-glucosidase inhibitor, DPP-IV inhibitor
DSNILEQGH	1012.04	0.20	0.06	Globulin	GH, QG, IL	ACE inhibitor, glucose uptake stimulation, DPP-IV inhibitor
KEGSLLLPHYNSKA	1556.78	0.37	0.17	Globulin	HY, LLP, GS, EG, KA, KE, PH, YN, LP, LLPH, PHY, LLL, LL, SL, SK	ACE inhibitor, antioxidant, antiinflammation, stimulating vasoactive substance release, glucose uptake stimulation, DPP-IV inhibitor
WFEITPEKNPQ	1388.54	0.23	0.11	Globulin	EI, PQ, EK, TP, PE, NP, WF, EI	ACE inhibitor, α-glucosidase inhibitor, DPP-IV inhibitor
AFGINAENNQRN	1347.41	0.20	0.12	Globulin	AF, GI, FG, AE, IN, NA, NN, NQ, RN	ACE inhibitor, DPP-IV inhibitor
KSLSSEDQPFN	1251.32	0.30	0.07	Globulin	QP, SE, SL, DQ, FN, KS, PF	ACE inhibitor, stimulating vasoactive substance release, DPP-IV inhibitor
LAVPVNNPHRFQD	1506.68	0.27	0.18	Globulin	AVP, RF, LA, VP, PH, AV, FQ, PHR, NP, HR, NN, PV, QD, VN	ACE inhibitor, antioxidant, DPP-IV inhibitor
KEGSLLLPH	993.17	0.41	0.21	Globulin	LLP, GS, EG, KE, PH, LP, LLL, LL, LLPH, SL, EG	ACE inhibitor, antioxidant, stimulating vasoactive substance release, glucose uptake stimulation, DPP-IV inhibitor
NVISEIPTEV	1100.23	0.41	0.07	Globulin	IP, EI, EV, TE, PT, EIPT, SE, NV, TE, VI	ACE inhibitor, antibacterial, stimulating vasoactive substance release, DPP-IV inhibitor
FLVNPDDNENLR	1445.55	0.41	0.14	Globulin	VNP, LR, NLR, LV, NEN, NP, FL, DN, NE, NL, VN	ACE inhibitor, antioxidant, stimulating vasoactive substance release, DPP-IV inhibitor
KSLSSEDQPF	1137.21	0.29	0.07	Globulin	QP, SE, SL, DQ, KS, PF	ACE inhibitor, stimulating vasoactive substance release, DPP-IV inhibitor

EGSLLLPH	865.00	0.50	0.13	Globulin	LLP, GS, EG, PH, LP, LLL, LL, LLPH, SL	ACE inhibitor, antioxidant, stimulating vasoactive substance release, glucose uptake stimulation, DPP-IV inhibitor
ASFDSDIKE	1011.05	0.31	0.06	Globulin	SF, KE, AS	ACE inhibitor, DPP-IV inhibitor
TLVNPDGRDSNILE	1542.67	0.23	012	Globulin	GR, DG, VNP, IL, LV, NP, TL, VN	ACE inhibitor, glucose uptake stimulation, DPP-IV inhibitor
EGSLLLPHYNS	1229.35	0.28	0.08	Globulin	HY, LLP, GS, EG, PH, YN, LP, LLL, LL, LLPH, PHY, SL	ACE inhibitor, antioxidant, antiinflammation, stimulating vasoactive substance release, glucose uptake stimulation, DPP-IV inhibitor
ILEQGHAQKIPAGTT	1563.77	0.20	0.16	Globulin	IPA, IP, AG, GH, GT, QG, QK, IL, PA, HA, KI, TT	ACE inhibitor, glucose uptake stimulation, DPP-IV inhibitor
EASFDSDIK	1011.05	0.27	0.06	Globulin	EA, SF, AS	ACE inhibitor, α-glucosidase inhibitor, DPP-IV inhibitor
TLVNPDGRDSNIL	1413.55	0.24	0.14	Globulin	GR, DG, VNP, IL, LV, NP, TL, VN	ACE inhibitor, glucose uptake stimulation, DPP-IV inhibitor
SFDSDIKEI	1053.13	0.37	0.05	Globulin	SF, EI, KE	ACE inhibitor, DPP-IV inhibitor
FRNQFGHL	1018.14	0.75	0.13	Globulin	FR, GH, HL, FG, NQ, QF, RN	ACE inhibitor, antioxidant, DPP-IV inhibitor
AELSEDDVFIIPATYPV	1879.09	0.22	0.07	Globulin	VF, IPA, YP, IP, AEL, II, SE, EL, TY, PA, AE, AT, II, PV	ACE inhibitor, antioxidant, stimulating vasoactive substance release, glucose uptake stimulation, α-glucosidase inhibitor, DPP-IV inhibitor
SFDSDIKE	939.97	0.35	0.05	Globulin	SF, KE	ACE inhibitor, DPP-IV inhibitor
DLDMFIR	909.07	0.76	0.10	Globulin	IR, MF, DM	ACE inhibitor, antioxidant, DPP-IV inhibitor
AELSEDDVFIIPA	1418.56	0.27	0.06	Globulin	VF, IPA, IP, AEL, II, SE, EL, PA, AE	ACE inhibitor, antioxidant, stimulating vasoactive substance release, glucose uptake stimulation, DPP-IV inhibitor
FEITPEKNPQLRD	1586.76	0.22	0.14	Globulin	EI, PQ, EK, TP, LR, KNPQLR, EITPEKNPQLR, PE, NP, QL	ACE inhibitor, fatty acid synthase inhibitor, α-glucosidase inhibitor, DPP-IV inhibitor
EASFDSDIKE	1140.17	0.21	0.05	Globulin	EA, SF, KE, AS	ACE inhibitor, α-glucosidase inhibitor, DPP-IV inhibitor
FEITPEKNPQL	1315.49	0.23	0.11	Globulin	EI, PQ, EK, TP, PE, NP, QL	ACE inhibitor, α-glucosidase inhibitor, DPP-IV inhibitor
MKEGSLLLPH	1124.36	0.30	0.19	Globulin	LLP, GS, EG, KE, PH, LP, LLL, LL, LLPH, LP, SL, MK	ACE inhibitor, antioxidant, stimulating vasoactive substance release, glucose uptake stimulation, DPP-IV inhibitor
LAVPVNNPH	960.10	0.23	0.18	Globulin	AVP, LA, VP, PH, AV, NP, NN, PV, VN	ACE inhibitor, DPP-IV inhibitor
GSLLLPH	735.88	0.61	0.19	Globulin	LLP, GS, PH, LP, LLL, LL, LLPH, LP	ACE inhibitor, antioxidant, stimulating vasoactive substance release, glucose uptake stimulation, DPP-IV inhibitor

NALKPDHRVETDGGL	1621.77	0.27	0.21	Glycinin	LKP, GL, GG, DG, KP, VE, LK, AL, ET, HR, NA, TD, VE	ACE inhibitor, antioxidant, *α*-glucosidase inhibitor, DPP-IV inhibitor
IHQSGPGHVIL	1157.34	0.40	0.07	Glycinin	PG, GP, GH, SG, IL, SGP, QSGP, HV, IH, QS, VI	ACE inhibitor, antiamnestic, antithrombotic, glucose uptake stimulation, regulating the stomach mucosal membrane activity, DPP-IV inhibitor
LAGNPDIEHPEAM	1393.53	0.42	0.08	Glycinin	LA, AG, EA, IE, HP, NP, EH, PE	ACE inhibitor, *α*-glucosidase inhibitor, DPP-IV inhibitor
YLAGNPDIEHPEA	1425.52	0.20	0.07	Glycinin	YL, LA, AG, EA, IE, HP, NP, EH, PE	ACE inhibitor, neuropeptide, *α*-glucosidase inhibitor, DPP-IV inhibitor
VFDDELSKGQ	1137.21	0.21	0.08	Glycinin	VF, KG, GQ, EL, SK	ACE inhibitor, antioxidant, neuropeptide, DPP-IV inhibitor
AGNPDIEHPEA	1149.18	0.26	0.06	Glycinin	AG, EA, IE, HP, NP, EH, PE	ACE inhibitor, *α*-glucosidase inhibitor, DPP-IV inhibitor
RNGLQMPSYSPY	1412.58	0.25	0.10	Glycinin	PSY, GL, NG, SY, LQ, PY, MP, SP, PS, RN, YS	ACE inhibitor, antiinflammation, DPP-IV inhibitor
IHQSGPGHVILIPR	1523.80	0.60	0.09	Glycinin	PG, GP, PR, IP, GH, SG, IL, SGP, QSGP, LI, HV, IH, QS, VI	ACE inhibitor, antiamnestic, antithrombotic, glucose uptake stimulation, regulating the stomach mucosal membrane activity, DPP-IV inhibitor
EVEPLPPR	936.08	0.47	0.25	Glycinin	PLP, LPP, PR, PL, EV, VE, PP, LP, EP	ACE inhibitor, *α*-glucosidase inhibitor, DPP-IV inhibitor
EIEPLPPR	950.10	0.56	0.15	Glycinin	PLP, LPP, PR, PL, EI, IE, PP, IEP, LP, EP	ACE inhibitor, *α*-glucosidase inhibitor, DPP-IV inhibitor
RAFPAEV	788.90	0.27	0.22	Glycinin	FP, AFP, AF, EV, PA, RA, AE	ACE inhibitor, DPP-IV inhibitor
VFDDELSKG	1009.08	0.28	0.08	Glycinin	VF, KG, EL, SK	ACE inhibitor, antioxidant, DPP-IV inhibitor
FDDELSKGQ	1038.08	0.25	0.07	Glycinin	KG, GQ, EL, SK	ACE inhibitor, antioxidant, neuropeptide, DPP-IV inhibitor
LSKGQLL	757.93	0.37	0.44	Glycinin	KG, GQ, LL, QL, SK	ACE inhibitor, neuropeptide, glucose uptake stimulation, DPP-IV inhibitor
AGNPDIEHPEAM	1280.37	0.45	0.07	Glycinin	AG, EA, IE, HP, NP, EH, PE	ACE inhibitor, *α*-glucosidase inhibitor, DPP-IV inhibitor
VQLNEGSIMGPHWNPK	1807.06	0.38	0.12	Vicilin-like	GP, GS, MG, EG, LN, PH, MGP, PHW, NP, WN, HW, IM, NE, PK, QL, SI, VQ	ACE inhibitor, antioxidant, antiamnestic, antithrombotic, regulating the stomach mucosal membrane activity, DPP-IV inhibitor
NEGSIMGPHWNPK	1466.63	0.52	0.09	Vicilin-like	GP, GS, MG, EG, PH, MGP, PHW, NP, WN, HW, IM, NE, PK, SI	ACE inhibitor, antioxidant, antiamnestic, antithrombotic, regulating the stomach mucosal membrane activity, DPP-IV inhibitor

YEAGVVPAPR	1058.20	0.23	0.21	Oleosin	PR, AP, VP, AG, GV, EA, PAP, YE, PA, VV	ACE inhibitor, α-glucosidase inhibitor, DPP-IV inhibitor

[a]From PeptideRanker. [b]From CPPpred. [c]From ExPASy ProtParam. [d]From BIOPEP.

Table S6. Potential bioactive peptides in IPP-soy protein.

Peptide sequence	MW	PeptideRanker score[a]	CPPpred sCore[b]	Parent protein	Potential bioactive peptides[d]	Biological function[d]
KTNDTPMIGTLAGANSLL	1817.09	0.34	0.22	Glycinin	LA, IG, GA, AG, GT, TP, LL, SL, KT, MI, ND, PM, TL, TN	ACE inhibitor, glucose uptake stimulation, DPP-IV inhibitor
SIIDTNSLENQLDQMPR	1974.17	0.24	0.10	Glycinin	PR, II, SL, MP, DQ, NQ, QL, SI, TN	ACE inhibitor, glucose uptake stimulation, DPP-IV inhibitor
VSIIDTNSLENQLDQMPRRF	2376.67	0.27	0.12	Glycinin	RF, PR, RR, II, MP, SL, DQ, NQ, QL, SI, TN, VS	ACE inhibitor, glucose uptake stimulation, DPP-IV inhibitor
FLEHAFSVDKQIA	1504.71	0.22	0.09	Glycinin	IA, AF, HA, FL, EH, QI, SV, VD	ACE inhibitor, DPP-IV inhibitor
NALPEEVIQHTFNLK	1752.99	0.25	0.14	Glycinin	EV, TF, ALP, LP, EE, LK, AL, FN, HT, IQ, NA, NL, QH, VI, PE	ACE inhibitor, antioxidant, stimulating vasoactive substance release, α-glucosidase inhibitor, DPP-IV inhibitor
NSLENQLDQMPRRF	1747.95	0.42	0.20	Glycinin	RF, PR, RR, MP, SL, DQ, NQ, QL	ACE inhibitor, DPP-IV inhibitor
TPMIGTLAGANSLL	1358.62	0.39	0.20	Glycinin	LA, IG, GA, AG, GT, TP, LL, SL, MI, PM, TL	ACE inhibitor, glucose uptake stimulation, DPP-IV inhibitor
PFKFLVPPQESQKR	1701.00	0.41	0.27	Glycinin	VPP, KR, VP, QK, KF, PP, PQ, LV, FL, ES, KF, QE	ACE inhibitor, antiinflammation, glucose uptake stimulation, α-glucosidase inhibitor, DPP-IV inhibitor
IIDTNSLENQLDQMPRRF	2190.46	0.24	0.13	Glycinin	RF, PR, RR, II, MP, SL, DQ, NQ, QL, TN	ACE inhibitor, glucose uptake stimulation, DPP-IV inhibitor
NSIIYALNGRAL	1304.51	0.48	0.14	Glycinin	IY, RA, YA, GR, NG, LN, IIY, II, AL, SI	ACE inhibitor, antioxidant, glucose uptake stimulation, DPP-IV inhibitor
KFLVPPQESQKRA	1527.79	0.24	0.36	Glycinin	VPP, KR, VP, RA, QK, KF, PP, PQ, LV, FL, ES, QE, FL	ACE inhibitor, antiinflammation, glucose uptake stimulation, α-glucosidase inhibitor, DPP-IV inhibitor
NANSIIYALNGRAL	1489.69	0.37	0.15	Glycinin	IY, RA, YA, GR, NG, LN, IIY, II, AL, SI	ACE inhibitor, antioxidant, glucose uptake stimulation, DPP-IV inhibitor
TPMIGTLAGANSL	1245.46	0.21	0.16	Glycinin	LA, IG, GA, AG, GT, TP, SL, MI, PM, TL	ACE inhibitor, DPP-IV inhibitor
SLENQLDQMPR	1330.48	0.31	0.16	Glycinin	PR, SL, MP, DQ, NQ, QL	ACE inhibitor, DPP-IV inhibitor

ARQIKNNNPFKFLVPPQES	2227.55	0.27	0.20	Glycinin	VPP, VP, KF, AR, PP, PQ, LV, NP, FL, ES, NN, QE, QI, FL	ACE inhibitor, antiinflammation, glucose uptake stimulation, *α*-glucosidase inhibitor, DPP-IV inhibitor
NALKPDNRIESEGGL	1612.76	0.28	0.14	Glycinin	LKP, GL, GG, EG, KP, IE, SE, LK, AL, DN, ES, NA, NR, RI	ACE inhibitor, antioxidant, stimulating vasoactive substance release, DPP-IV inhibitor
NLKSQQARQIKNNNPF	1900.13	0.31	0.26	Glycinin	AR, LK, NP, KS, NL, NN, PF, QA, QI, QQ	ACE inhibitor, antioxidant, DPP-IV inhibitor
DQMPRRFYLAGNQEQEF	2129.33	0.41	0.20	Glycinin	RF, PR, YL, FY, YLAGNQ, LA, AG, RR, EF, YLAGNQEQE, MP, DQ, NQ, QE	ACE inhibitor, antioxidant, neuropeptide, hypolipidemic, DPP-IV inhibitor
KSQQARQIKNNNPFKF	1948.21	0.61	0.26	Glycinin	KF, AR, NP, KS, NN, PF, QA, QI, QQ	ACE inhibitor, DPP-IV inhibitor
ENQLDQMPR	1130.24	0.35	0.17	Glycinin	PR, MP, DQ, NQ, QL	ACE inhibitor, DPP-IV inhibitor
SAEFGSLRKNAMF	1457.67	0.78	0.14	Glycinin	MF, FG, GS, LR, EF, SLR, SL, AE, NA, RK, EF	ACE inhibitor, hypolipidemic, DPP-IV inhibitor
NSLENQLDQMPR	1444.58	0.24	0.15	Glycinin	PR, SL, MP, SL, DQ, NQ, QL	ACE inhibitor, DPP-IV inhibitor
ENQLDQMPRRF	1433.60	0.61	0.24	Glycinin	RF, PR, RR, MP, DQ, NQ, QL	ACE inhibitor, DPP-IV inhibitor
SAEFGSLRKNAM	1310.49	0.56	0.17	Glycinin	FG, GS, LR, EF, SLR, SL, AE, NA, RK, EF	ACE inhibitor, hypolipidemic, DPP-IV inhibitor
RKNAMFVPHYNLNA	1674.94	0.27	0.22	Glycinin	MF, HY, VP, LN, PH, FVP, YN, YNL, PHY, FVPH, NA, NL, RK	ACE inhibitor, antioxidant, antiinflammation, DPP-IV inhibitor
NPFKFLVPPQES	1402.61	0.29	0.10	Glycinin	VPP, VP, KF, PP, PQ, LV, NP, FL, ES, QE, FL	ACE inhibitor, antiinflammation, glucose uptake stimulation, *α*-glucosidase inhibitor, DPP-IV inhibitor
QIKNNNPFKFLVPPQESQ	2128.42	0.27	0.13	Glycinin	VPP, VP, KF, PP, PQ, LV, NP, FL, ES, QE, QI, FL	ACE inhibitor, antiinflammation, glucose uptake stimulation, *α*-glucosidase inhibitor, DPP-IV inhibitor
FEEPQQPQQRGQSSRPQDR	2298.42	0.26	0.25	Glycinin	RP, GQ, PQ, RG, QP, DR, EE, EP, QD, QQ, QS	ACE inhibitor, neuropeptide, stimulating vasoactive substance release, DPP-IV inhibitor
FVPHYNLNANSI	1388.55	0.34	0.08	Glycinin	HY, VP, LN, PH, FVP, YN, YNL, PHY, FVPH, NA, NL, SI	ACE inhibitor, antioxidant, antiinflammation, DPP-IV inhibitor
NALPEEVIQHTFNL	1624.81	0.28	0.10	Glycinin	EV, TF, ALP, LP, EE, AL, FN, HT, IQ, NA, NL, QH, VI, PE	ACE inhibitor, stimulating vasoactive substance release, *α*-glucosidase inhibitor, DPP-IV inhibitor

FREGDLIAVPTGVA	1444.65	0.25	0.16	Glycinin	AVP, IA, VP, FR, GV, GD, TG, EG, PT, AV, IAVPTGVA, LI, VA	ACE inhibitor, glucose uptake stimulation, DPP-IV inhibitor
NQLDQMPRRF	1304.49	0.75	0.32	Glycinin	RF, PR, RR, MP, DQ, NQ, QL	ACE inhibitor, DPP-IV inhibitor
KGAIVTVKGGL	1042.29	0.42	0.38	Glycinin	VK, GA, GL, KG, GG, AI, IV, TV, VT	ACE inhibitor, glucose uptake stimulation, DPP-IV inhibitor
FREGDLIAVPT	1217.39	0.24	0.14	Glycinin	AVP, IA, VP, FR, GD, EG, PT, AV, LI	ACE inhibitor, glucose uptake stimulation, DPP-IV inhibitor
NANSIIYALNG	1149.27	0.31	0.08	Glycinin	IY, YA, NG, LN, IIY, II, AL, NA, SI	ACE inhibitor, antioxidant, glucose uptake stimulation, DPP-IV inhibitor
GRVLIVPQNF	1142.37	0.42	0.19	Glycinin	VLIVP, VP, GR, NF, PQ, VL, IV, LI, NF, QN	ACE inhibitor, glucose uptake stimulation, DPP-IV inhibitor
PEEVIQHTFNL	1326.47	0.30	0.08	Glycinin	EV, TF, EE, FN, HT, IQ, NL, QH, VI, PE	ACE inhibitor, stimulating vasoactive substance release, α-glucosidase inhibitor, DPP-IV inhibitor
TSLDFPALSWLRLSAE	1806.05	0.53	0.27	Glycinin	RL, FP, LSW, DF, WL, LR, SL, PA, AL, AE, SW, TS, SWLRL, WLRL	ACE inhibitor, antioxidant, antiinflammation, α-glucosidase inhibitor, DPP-IV inhibitor
TNSLENQLDQMPRRF	1849.05	0.29	0.23	Glycinin	RF, PR, RR, MP, SL, DQ, NQ, QL, TN	ACE inhibitor, DPP-IV inhibitor
RKNAMFVPHYN	1376.60	0.28	0.18	Glycinin	MF, HY, VP, PH, FVP, YN, PHY, FVPH, NA, RK	ACE inhibitor, antioxidant, antiinflammation, DPP-IV inhibitor
NQLDQMPR	1001.13	0.47	0.24	Glycinin	PR, MP, DQ, NQ, QL	ACE inhibitor, DPP-IV inhibitor
GRVLIVPQNFVVA	1411.71	0.29	0.27	Glycinin	VLIVP, VP, GR, NF, PQ, VL, IV, LI, VA, VV, QN	ACE inhibitor, glucose uptake stimulation, DPP-IV inhibitor
REGDLIAVPTGVAW	1483.69	0.38	0.24	Glycinin	AVP, AW, IA, VP, GV, GD, TG, EG, PT, AV, IAVPTGVA, LI, VA, TG	ACE inhibitor, antioxidant, glucose uptake stimulation, DPP-IV inhibitor
QIKNNNPFKFLVPPQES	2000.28	0.34	0.12	Glycinin	VPP, VP, KF, PP, PQ, LV, NP, FL, ES, NN, PF, QE, QI	ACE inhibitor, antiinflammation, glucose uptake stimulation, PP-IV inhibitor
NSIIYALNGRA	1191.35	0.35	0.12	Glycinin	IY, RA, YA, GR, NG, LN, IIY, II, AL, SI	ACE inhibitor, antioxidant, glucose uptake stimulation, DPP-IV inhibitor
IYIQQGKGIFG	1223.44	0.47	0.06	Glycinin	IY, IF, GI, KG, FG, GK, QG, IFG, IQ, QQ	ACE inhibitor, antioxidant, DPP-IV inhibitor
LRLSAEFGS	979.10	0.20	0.15	Glycinin	RL, FG, GS, LR, EF, AE	ACE inhibitor, hypolipidemic, DPP-IV inhibitor
VIQHTFNLK	1099.30	0.29	0.15	Glycinin	TF, LK, FN, HT, IQ, NL, QH, VI	ACE inhibitor, antioxidant, DPP-IV inhibitor

PALSWLRL	955.17	0.92	0.61	Glycinin	RL, LSW, WL, LR, PA, AL, SW, SWLRL, WLRL	ACE inhibitor, antioxidant, antiinflammation, *α*-glucosidase inhibitor, DPP-IV inhibitor
LNANSIIYALNGRA	1489.69	0.27	0.15	Glycinin	IY, RA, YA, GR, NG, LN, IIY, II, AL, NA, SI	ACE inhibitor, antioxidant, glucose uptake stimulation, DPP-IV inhibitor
PFKFLVPPQESQ	1416.64	0.26	0.11	Glycinin	VPP, VP, KF, PP, PQ, LV, FL, ES, PF, QE	ACE inhibitor, antiinflammation, glucose uptake stimulation, *α*-glucosidase inhibitor, DPP-IV inhibitor
IAVPTGVAWW	1099.30	0.60	0.29	Glycinin	AVP, AW, IA, VP, GV, TG, PT, AV, IAVPTGVA, TG	ACE inhibitor, antioxidant, DPP-IV inhibitor
FEEPQQPQQRGQ	1471.55	0.21	0.14	Glycinin	GQ, PQ, RG, QP, EE, EP, QQ	ACE inhibitor, neuropeptide, stimulating vasoactive substance release, DPP-IV inhibitor
TSLDFPAL	862.98	0.54	0.12	Glycinin	FP, DF, SL, PA, AL, TS	ACE inhibitor, DPP-IV inhibitor
LKSQQARQIKNNNPF	1786.02	0.33	0.27	Glycinin	AR, LK, NP, KS, NN, PF, QA, QI, QQ	ACE inhibitor, antioxidant, DPP-IV inhibitor
IYIQQGKGIF	1166.39	0.42	0.06	Glycinin	IY, IF, GI, KG, GK, QG, IQ, QQ	ACE inhibitor, antioxidant, DPP-IV inhibitor
VPTGVAWWMYNNEDTPV	1979.19	0.21	0.18	Glycinin	MY, AW, YP, GV, TG, PT, TP, WM, YN, WMY, VA, NE, NN, PV	ACE inhibitor, antioxidant, DPP-IV inhibitor
IQKLNALKPDNRIESEGGL	2095.38	0.33	0.18	Glycinin	LKP, GL, GG, EG, QK, KL, KP, IE, LN, SE, LK, AL, DN, ES, IQ, LN, NA, NR, RI	ACE inhibitor, antioxidant, stimulating vasoactive substance release, DPP-IV inhibitor
TATSLDFPALSWLRLSAE	1978.23	0.90	0.31	Glycinin	RL, FP, LSW, DF, WL, LR, SL, PA, TA, AL, AE, AT, TS, SWLRL, WLRL	ACE inhibitor, antioxidant, antiinflammation, *α*-glucosidase inhibitor, DPP-IV inhibitor
TSLDFPALSWLRL	1518.78	0.36	0.34	Glycinin	RL, FP, LSW, DF, WL, LR, SL, PA, TA, AL, AT, TS, SWLRL, WLRL	ACE inhibitor, antioxidant, antiinflammation, *α*-glucosidase inhibitor, DPP-IV inhibitor
GRVLIVPQ	881.09	0.21	0.34	Glycinin	VLIVP, VP, GR, PQ, VL, IV, LI	ACE inhibitor, glucose uptake stimulation, DPP-IV inhibitor
KTNDTPMIGT	1077.22	0.20	0.14	Glycinin	IG, GT, TP, KT, MI, ND, PM, TN	ACE inhibitor, DPP-IV inhibitor
IQQGKGIFG	947.10	0.54	0.08	Glycinin	IF, GI, KG, FG, GK, QG, IFG, IQ, QQ	ACE inhibitor, DPP-IV inhibitor
IAVPTGVAWWM	1230.49	0.80	0.28	Glycinin	AVP, AW, IA, VP, GV, TG, PT, AV, WM, IAVPTGVA, TG	ACE inhibitor, antioxidant, DPP-IV inhibitor

LNALPEEVIQHTFNL	1737.97	0.34	0.12	Glycinin	EV, LN, TF, ALP, LP, EE, AL, FN, HT, IQ, NA, NL, QH, VI, PE	ACE inhibitor, stimulating vasoactive substance release, *α*-glucosidase inhibitor, DPP-IV inhibitor
LKPDNRIESEGGL	1427.58	0.26	0.13	Glycinin	LKP, GL, GG, EG, KP, IE, SE, LK, ES, NR, RI	ACE inhibitor, antioxidant, stimulating vasoactive substance release, DPP-IV inhibitor
LQGENEGEDKGAIVTV KGGL	2014.22	0.31	0.16	Glycinin	VK, GA, GL, KG, GE, GG, QG, AI, EG, LQ, IV, NE, TV, VT	ACE inhibitor, glucose uptake stimulation, DPP-IV inhibitor
VLIVPQNFVVA	1198.47	0.23	0.18	Glycinin	VLIVP, VP, NF, PQ, VL, IV, LI, VA, VV	ACE inhibitor, glucose uptake stimulation, DPP-IV inhibitor
IAVPTGVAW	913.08	0.33	0.23	Glycinin	AVP, AW, IA, VP, GV, TG, PT, AV, IAVPTGVA	ACE inhibitor, antioxidant, DPP-IV inhibitor
NNNPFKFLVPPQES	1630.82	0.29	0.11	Glycinin	VPP, VP, KF, PP, PQ, LV, NP, FL, ES, NN, QE	ACE inhibitor, antiinflammation, glucose uptake stimulation, DPP-IV inhibitor
FLEHAFSVD	1064.16	0.23	0.06	Glycinin	AF, HA, FL, EH, SV, VD	ACE inhibitor, DPP-IV inhibitor
IKNNNPFKFLVPPQES	1872.15	0.28	0.12	Glycinin	VPP, VP, KF, PP, PQ, LV, NP, FL, ES, NN, QE	ACE inhibitor, antiinflammation, glucose uptake stimulation, *α*-glucosidase inhibitor, DPP-IV inhibitor
VSFKTNDTPMIGTL	1523.76	0.32	0.13	Glycinin	IG, GT, SF, TP, KT, MI, ND, PM, TL, TN, VS	ACE inhibitor, DPP-IV inhibitor
TPMIGTLAGAN	1045.22	0.21	0.17	Glycinin	LI, IG, GA, AG, GT, TP, MI, PM, TL	ACE inhibitor, DPP-IV inhibitor
FLEHAFSVDKQIAK	1632.88	0.23	0.12	Glycinin	IA, AF, IAK, HA, FL, EH, QI, SV, CD	ACE inhibitor, hypotensive, antibacterial, DPP-IV inhibitor
PFKFLVPPQES	1288.51	0.32	0.10	Glycinin	VPP, VP, KF, PP, PQ, LV, FL, ES, NN, QE	ACE inhibitor, antiinflammation, glucose uptake stimulation, *α*-glucosidase inhibitor, DPP-IV inhibitor
VPHYNLNA	927.03	0.34	0.12	Glycinin	HY, VP, LN, PH, YN, YNL, PHY, NA, NL	ACE inhibitor, antioxidant, antiinflammation, DPP-IV inhibitor
IIYALNGRA	990.17	0.34	0.15	Glycinin	IY, RA, YA, GR, NG, LN, IIY, II, AL	ACE inhibitor, antioxidant, glucose uptake stimulation, DPP-IV inhibitor
NNPFKFLVPPQES	1516.72	0.29	0.10	Glycinin	VPP, VP, KF, PP, PQ, LV, NP, FL, ES, NN, QE	ACE inhibitor, antiinflammation, glucose uptake stimulation, *α*-glucosidase inhibitor, DPP-IV inhibitor
FREGDLIAVPTGV	1373.57	0.27	0.15	Glycinin	AVP, IA, VP, FR, GV, GD, TG, PT, AV, LI	ACE inhibitor, glucose uptake stimulation, DPP-IV inhibitor
IETWNPNNKPFQ	1487.63	0.39	0.12	Glycinin	NK, KP, IE, FQ, KP, TW, NP, WN, ET, NN, PF, PN	ACE inhibitor, antioxidant, DPP-IV inhibitor
ATSLDFPAL	934.06	0.54	0.15	Glycinin	FP, DF, SL, PA, AL, AT, TS	ACE inhibitor, DPP-IV inhibitor

TSLDFPA	749.82	0.41	0.09	Glycinin	FP, DL, SL, PA, TS	ACE inhibitor, DPP-IV inhibitor
YIQQGKGIFG	1110.28	0.49	0.07	Glycinin	IF, GI, KG, FG, GK, QG, IFG, IQ, QQ, YI	ACE inhibitor, DPP-IV inhibitor
NANSIIYAL	978.11	0.41	0.08	Glycinin	IY, YA, IIY, II, AL, NA, SI	ACE inhibitor, antioxidant, glucose uptake stimulation, DPP-IV inhibitor
NNNPFKFLVPPQESQ	1758.95	0.24	0.11	Glycinin	VPP, VP, KF, PP, PQ, LV, NP, FL, ES, NN, QE	ACE inhibitor, antiinflammation, glucose uptake stimulation, α-glucosidase inhibitor, DPP-IV inhibitor
IIYALNG	762.90	0.30	0.07	Glycinin	IY, YA, NG, LN, IIY, II, AL, II	ACE inhibitor, antioxidant, glucose uptake stimulation, DPP-IV inhibitor
TSLDFPALS	950.06	0.32	0.10	Glycinin	FP, DF, PA, AL, SL, TS	ACE inhibitor, DPP-IV inhibitor
IAVPTGVAWWMYNNE DTPV	2163.43	0.25	0.17	Glycinin	AVP, MY, AW, IA, VP, GV, TG, PT, AV, TP, WM, YN, IAVPTGVA, WMY, VA, WW, NE, NN, PV	ACE inhibitor, antioxidant, DPP-IV inhibitor
FREGDLIAVPTGVAW	1630.86	0.46	0.20	Glycinin	AVP, AW, IA, VP, FR, GV, GD, TG, EG, PT, AV, IAVPTGVA, VA, IA	ACE inhibitor, antioxidant, glucose uptake stimulation, DPP-IV inhibitor
AIVTVKGGL	857.06	0.21	0.32	Glycinin	VK, GL, KG, GG, AI, IV, TV, VT	ACE inhibitor, glucose uptake stimulation, DPP-IV inhibitor
RSQSDNFEYV	1244.28	0.27	0.07	Glycinin	NF, EY, YV, DN, QS	ACE inhibitor, DPP-IV inhibitor
KTNDTPMIGTL	1190.38	0.30	0.18	Glycinin	IG, GT, TP, KT, MI, ND, PM, TL, TN	ACE inhibitor, DPP-IV inhibitor
YVSFKTNDTPMIGT	1573.78	0.20	0.09	Glycinin	IG, GT, SF, TP, YV, KT, MI, ND, PM, TL, TN, VS	ACE inhibitor, DPP-IV inhibitor
IYNFREGDL	1126.23	0.28	0.08	Glycinin	IY, FR, GD, EG, NF, YN	ACE inhibitor, antioxidant, DPP-IV inhibitor
ALNGRALI	826.99	0.27	0.38	Glycinin	RA, GR, NG, LN, LI, AL	ACE inhibitor, glucose uptake stimulation, DPP-IV inhibitor
FREGDL	735.79	0.43	0.13	Glycinin	FR, GD, EG	ACE inhibitor, DPP-IV inhibitor
VLIVPQNFVVAA	1269.55	0.25	0.20	Glycinin	VAA, VLIVP, VP, AA, NF, PQ, VL, IV, LI, VA, VV, QN, VL	ACE inhibitor, hypotensive, glucose uptake stimulation, DPP-IV inhibitor
FLEHAFSVDKQ	1320.47	0.23	0.09	Glycinin	AF, HA, FL, EH, SV, VD	ACE inhibitor, DPP-IV inhibitor
IVPQNFVVAA	1057.26	0.25	0.14	Glycinin	VAA, VP, AA, NF, PQ, IV, VA, VV, QN	ACE inhibitor, hypotensive, glucose uptake stimulation, DPP-IV inhibitor

IAVPTGVAWWMYNNED	1866.08	0.34	0.14	Glycinin	AVP, MY, AW, IA, VP, GV, TG, PT, AV, TP, WM, YN, IAVPTGVA, WMY, VA, WW, NE, NN, PV	ACE inhibitor, antioxidant, DPP-IV inhibitor
LIAVPTGVAW	1026.24	0.37	0.28	Glycinin	AVP, AW, IA, VP, GV, TG, PT, AV, IAVPTGVA, LI, VA	ACE inhibitor, antioxidant, glucose uptake stimulation, DPP-IV inhibitor
IGTLAGANSLLNAL	1327.54	0.51	0.23	Glycinin	LA, IG, GA, AG, GT, LN, LL, SL, AL, NA, TL	ACE inhibitor, glucose uptake stimulation, DPP-IV inhibitor
ALKPDNRIESEGGL	1498.66	0.32	0.14	Glycinin	LKP, GL, GG, EG, KP, IE, SE, LK, AL, DN, ES, NR, RI	ACE inhibitor, antioxidant, stimulating vasoactive substance release, DPP-IV inhibitor
AWWMYNNEDTPV	1525.66	0.33	0.13	Glycinin	MY, AW, TP, WM, YN, WMY, WW, NE, NN, PV	ACE inhibitor, antioxidant, DPP-IV inhibitor
FVPHYNLNANSIIYALNG	2020.27	0.44	0.08	Glycinin	IY, HY, VP, YA, NG, LN, PH, FVP, YN, IIY, YNL, II, PHY, FVPH, AL, NA, NL, SI	ACE inhibitor, antioxidant, antiinflammation, glucose uptake stimulation, DPP-IV inhibitor
LNALPEEVIQHTFN	1624.81	0.22	0.10	Glycinin	EV, LN, TF, ALP, LP, EE, AL, FN, HT, IQ, NA, QH, VI, PE	ACE inhibitor, stimulating vasoactive substance release, α-glucosidase inhibitor, DPP-IV inhibitor
NFREGDL	849.90	0.31	0.12	Glycinin	FR, GD, EG, NF	ACE inhibitor, DPP-IV inhibitor
NPFKFLVPPQE	1315.53	0.40	0.13	Glycinin	VPP, VP, KF, PP, PQ, LV, NP, FL, PF	ACE inhibitor, antiinflammation, glucose uptake stimulation, α-glucosidase inhibitor, DPP-IV inhibitor
TATSLDFPA	922.00	0.22	0.14	Glycinin	FP, DF, SL, PA, TA, AT, TS	ACE inhibitor, DPP-IV inhibitor
QQGKGIF	776.89	0.60	0.09	Glycinin	IF, GI, KG, GK, QG, QQ	ACE inhibitor, DPP-IV inhibitor
WMYNNEDT	1072.11	0.20	0.08	Glycinin	MY, WM, YN, WMY, NE, NN	ACE inhibitor, antioxidant, DPP-IV inhibitor
FVPHYNL	889.02	0.69	0.08	Glycinin	HY, VP, PH, FVP, YN, YNL, PHY, FVPH, NL	ACE inhibitor, antioxidant, antiinflammation, DPP-IV inhibitor
SQQARQIKNNNPF	1544.69	0.41	0.17	Glycinin	AR, NP, NN, PF, QA, QI, QQ	ACE inhibitor, DPP-IV inhibitor
EPQQPQQRGQSSRPQ	1750.85	0.24	0.26	Glycinin	RP, GQ, PQ, RG, QP, EP, QQ, QS	ACE inhibitor, neuropeptide, DPP-IV inhibitor
IIYALNGRAL	1103.33	0.44	0.18	Glycinin	IY, RA, YA, GR, NG, LN, IIY, II, AL	ACE inhibitor, antioxidant, glucose uptake stimulation, DPP-IV inhibitor
KFLVPPQE	957.14	0.24	0.18	Glycinin	VPP, VP, KF, PP, PQ, LV, FL, QE	ACE inhibitor, antiinflammation, glucose uptake stimulation, α-glucosidase inhibitor, DPP-IV inhibitor
SWLRL	673.81	0.88	0.68	Glycinin	RL, WL, LR, SW, SWLRL, WLRL	ACE inhibitor, α-glucosidase inhibitor, DPP-IV inhibitor

AWWMYNNEDTPVV	1624.79	0.25	0.15	Glycinin	MY, AW, TP, WM, YN, WMY, WW, NE, NN, PV	ACE inhibitor, antioxidant, DPP-IV inhibitor
KNAMFVPHYNLNA	1518.75	0.39	0.15	Glycinin	MF, HY, VP, LN, PH, FVP, YN, YNL, PHY, FVPH, NA, NL	ACE inhibitor, antioxidant, antiinflammation, DPP-IV inhibitor
FSVDKQIA	907.03	0.20	0.09	Glycinin	IA, QI, SV, VD	ACE inhibitor, DPP-IV inhibitor
FVPHYNLNANS	1275.39	0.28	0.08	Glycinin	HY, VP, LN, PH, FVP, YN, YNL, PHY, FVPH, NA, NL	ACE inhibitor, antioxidant, antiinflammation, DPP-IV inhibitor
RKNAMFVPHYTLNA	1661.94	0.20	0.26	Glycinin	MF, HY, VP, LN, PH, FVP, YN, YNL, PHY, FVPH, NA, NL	ACE inhibitor, antioxidant, antiinflammation, DPP-IV inhibitor
LKLSAQYGSLRKNAM	1680.00	0.34	0.41	Glycinin	LKL, YG, GS, KL, LR, SLR, SL, LK, NA, QY, RK	ACE inhibitor, antioxidant, antiinflammation, DPP-IV inhibitor
KTNDRPSIGNLAGANSLL	1841.05	0.52	0.21	Glycinin	RP, LA, IG, GA, AG, DR, LL, SL, KT, ND, NL, PS, SI, TN	ACE inhibitor, glucose uptake stimulation, DPP-IV inhibitor
KTNDRPSIGNLAGANSL	1727.89	0.28	0.	Glycinin	RP, LA, IG, GA, AG, DR, LL, SL, KT, ND, NL, PS, SI, TN	ACE inhibitor, DPP-IV inhibitor
DRPSIGNLAGANS	1271.35	0.25	0.11	Glycinin	RP, LA, IG, GA, AG, DR, NL, PS, SI	ACE inhibitor, DPP-IV inhibitor
RPSIGNLAGANSLL	1382.58	0.62	0.18	Glycinin	RP, LA, IG, GA, AG, LL, SL, NL, PS, SI	ACE inhibitor, glucose uptake stimulation, DPP-IV inhibitor
KTNDRPSIGNLAGAN	1527.66	0.25	0.19	Glycinin	RP, LA, IG, GA, AG, DR, LL, SL, KT, ND, NL, PS, SI, TN	ACE inhibitor, DPP-IV inhibitor
RPSIGNLAGANSL	1269.42	0.37	0.16	Glycinin	RP, LA, IG, GA, AG, SL, NL, PS, SI	ACE inhibitor, DPP-IV inhibitor
KTNDRPSIGNL	1214.34	0.33	0.17	Glycinin	RP, LA, IG, GA, AG, SL, NL, PS, SI	ACE inhibitor, DPP-IV inhibitor
NRIESEGGF	1008.06	0.21	0.06	Glycinin	GF, GG, EG, IE, SE, ES, NR, IR	ACE inhibitor, stimulating vasoactive substance release, DPP-IV inhibitor
AVAAKSQSDNFEYVSF	1762.89	0.25	0.08	Glycinin	VAA, AA, NF, SF, EY, AV, YV, AA, VA, DN, KS, QS, VS	ACE inhibitor, hypotensive, DPP-IV inhibitor
VSFKTNDRPSIGN	1434.57	0.23	0.11	Glycinin	RP, IG, SF, DR, KT, ND, PS, SI, TN, VS	ACE inhibitor, DPP-IV inhibitor
NLKSQQARQVKNNNPFS	1973.18	0.21	0.32	Glycinin	VK, AR, LK, NP, KS, NL, NN, PF, QA, QQ, QV	ACE inhibitor, antioxidant, DPP-IV inhibitor

GKQQEEENEGSNILSGFAPE	2163.24	0.30	0.07	Glycinin	FAP, AP, GF, GS, GK, SG, EG, IL, EEE, EE, FA, NE, QE, QQ, PE	ACE inhibitor, stimulating vasoactive substance release, glucose uptake stimulation, α-glucosidase inhibitor, DPP-IV inhibitor
DRPSIGNLAGANSLL	1497.67	0.70	0.15	Glycinin	RP, LA, IG, GA, AG, DR, LL, SL, NL, PS, SI	ACE inhibitor, glucose uptake stimulation, DPP-IV inhibitor
LKEAFGVNM	1008.20	0.28	0.16	Glycinin	AF, FG, GV, EA, KE, LK, NM, VN	ACE inhibitor, antioxidant, α-glucosidase inhibitor, DPP-IV inhibitor
NSIIYALNGRALVQ	1531.77	0.21	0.16	Glycinin	IY, RA, YA, GR, NG, LN, LVQ, IIY, LV, II, AL, SI, VQ	ACE inhibitor, antioxidant, glucose uptake stimulation, DPP-IV inhibitor
KSQQARQVKNNNPFSF	1893.09	0.49	0.22	Glycinin	VK, SF, AR, NP, KS, NN, PF, QA, QQ, QV	ACE inhibitor, DPP-IV inhibitor
FLVPPQESQRR	1356.55	0.31	0.28	Glycinin	VPP, VP, RR, PP, PQ, LV, FL, ES, QE	ACE inhibitor, antiinflammation, glucose uptake stimulation, α-glucosidase inhibitor, DPP-IV inhibitor
YIQQGNGIF	1039.16	0.40	0.05	Glycinin	IF, GI, QG, NG, IQ, QQ, YI	ACE inhibitor, DPP-IV inhibitor
SSPDIYNPQAGSITTA	1621.72	0.48	0.07	Glycinin	IY, AG, GS, ITT, PQ, YN, AGS, SP, NP, TA, QA, SI, TT	ACE inhibitor, antioxidant, DPP-IV inhibitor
LKLSAQYGSL	1079.26	0.24	0.20	Glycinin	LKL, YG, GS, KL, SL, LK, QY	ACE inhibitor, antioxidant, antiinflammation, DPP-IV inhibitor
PALWLLKLS	1040.31	0.66	0.59	Glycinin	LKL, LW, KL, WL, LL, LK, PA, AL	ACE inhibitor, antioxidant, glucose uptake stimulation, DPP-IV inhibitor
LKSQQARQVKNNNPFS	1859.07	0.24	0.32	Glycinin	VK, AR, LK, NP, KS, NN, PF, QA, QQ, QV	ACE inhibitor, antioxidant, DPP-IV inhibitor
SGAIVTVKGGLRVTA	1428.70	0.22	0.38	Glycinin	VK, GA, GL, KG, GG, AI, SG, LR, IV, TA, VK, VT	ACE inhibitor, glucose uptake stimulation, DPP-IV inhibitor
SSPDIYNPQAGSITT	1550.64	0.48	0.06	Glycinin	IY, AG, GS, ITT, PQ, YN, AGS, SP, NP, QA, SI, TT	ACE inhibitor, antioxidant, DPP-IV inhibitor
SGFAPEFLKEAFGV	1498.70	0.79	0.10	Glycinin	FAP, AFF, AP, GF, FG, GV, SG, EA, KE, EF, LK, FA, FL, AF, EA, PE	ACE inhibitor, antioxidant, hypolipidemic, glucose uptake stimulation, α-glucosidase inhibitor, DPP-IV inhibitor
FLKEAFGVN	1024.18	0.29	0.13	Glycinin	AF, FG, GV, EA, KE, LK, FL, VN	ACE inhibitor, antioxidant, α-glucosidase inhibitor, DPP-IV inhibitor
SGFAPEFLKEAFGVNMQIV	2084.42	0.84	0.09	Glycinin	FAP, AFF, AP, GF, FG, GV, SG, EA, KE, MQ, NM, QI, EF, LK, FA, FL, AF, EA, PE	ACE inhibitor, antioxidant, hypolipidemic, glucose uptake stimulation, α-glucosidase inhibitor, DPP-IV inhibitor
FLVPPQESQRRA	1427.63	0.29	0.31	Glycinin	VPP, VP, RA, RR, PP, PQ, LV, FL, ES, QE	ACE inhibitor, antiinflammation, glucose uptake stimulation, α-glucosidase inhibitor, DPP-IV inhibitor
IYIQQGNGIF	1152.32	0.36	0.05	Glycinin	IY, IF, GI, QG, NG, IQ, QQ, YI	ACE inhibitor, antioxidant, DPP-IV inhibitor

PHYTLNANSIIYAL	1589.81	0.29	0.09	Glycinin	IY, HY, YA, LN, PH, IIY, II, PHY, AL, YT, SI, TL	ACE inhibitor, antioxidant, antiinflammation, glucose uptake stimulation, DPP-IV inhibitor
RPSIGNL	755.87	0.52	0.15	Glycinin	RP, IG, NL, PS, SI	ACE inhibitor, DPP-IV inhibitor
KEAFGVNM	895.04	0.30	0.12	Glycinin	AF, FG, GV, EA, KE, NM, VN	ACE inhibitor, DPP-IV inhibitor
VPHYTLNA	914.03	0.23	0.15	Glycinin	HY, VP, LN, PH, PHY, YT, NA, TL	ACE inhibitor, antioxidant, antiinflammation, DPP-IV inhibitor
FAVAAKSQSDNFE	1413.51	0.21	0.09	Glycinin	VAA, AA, NF, AV, VA, FA, DN, KS, QS, FA	ACE inhibitor, hypotensive, DPP-IV inhibitor
WLLKL	671.88	0.75	0.80	Glycinin	LKL, KL, WL, LL, LK	ACE inhibitor, antioxidant, glucose uptake stimulation, DPP-IV inhibitor
SIGNLAGANSLL	1129.28	0.54	0.12	Glycinin	LA, IG, GA, AG, LL, SL, NL, SI	ACE inhibitor, glucose uptake stimulation, DPP-IV inhibitor
KTNDRPSIGNLAGA	1413.55	0.24	0.20	Glycinin	RP, LA, IG, GA, AG, DR, KT, ND, NL, PS, SI, TN	ACE inhibitor, DPP-IV inhibitor
VPHYTLN	842.95	0.35	0.13	Glycinin	HY, VP, LN, PH, PHY, YT, TL	ACE inhibitor, antioxidant, antiinflammation, DPP-IV inhibitor
KNNNPFSFLVPP	1373.57	0.74	0.11	Glycinin	VPP, VP, SF, PP, LV, NP, FL, NN, PF	ACE inhibitor, antiinflammation, glucose uptake stimulation, *α*-glucosidase inhibitor, DPP-IV inhibitor
YVSFKTNDRPSIGNL	1710.91	0.31	0.12	Glycinin	RP, IG, SF, YV, DR, KT, ND, NL, PS, SI, TN, VS	ACE inhibitor, DPP-IV inhibitor
QEIYIQQGNGIFGM	1597.81	0.63	0.06	Glycinin	IY, IF, GI, GM, FG, QG, NG, IFG, EI, IQ, NG, QE, QQ	ACE inhibitor, antioxidant, DPP-IV inhibitor
YIQQGNGIFG	1096.21	0.48	0.05	Glycinin	IF, GI, FG, QG, NG, IFG, IQ, QQ, YI	ACE inhibitor, DPP-IV inhibitor
TATSLDFPALWLLKLSA	1847.18	0.52	0.42	Glycinin	LKL, LW, FP, KL, DF, WL, LL, SL, LK, PA, AL, AT, TS	ACE inhibitor, antioxidant, glucose uptake stimulation, DPP-IV inhibitor
PFSFLVPPQESQR	1531.73	0.40	0.11	Glycinin	VPP, VP, SF, PP, PQ, LV, FL, ES	ACE inhibitor, antiinflammation, glucose uptake stimulation, DPP-IV inhibitor
PSIGNLAGANSLLNALPE	1750.97	0.64	0.14	Glycinin	LA, IG, GA, AG, LN, ALP, LP, LL, SL, LN, NA, NL, PS, SI, PE	ACE inhibitor, glucose uptake stimulation, *α*-glucosidase inhibitor, DPP-IV inhibitor
QNSSPDIYNPQAGSI	1590.67	0.32	0.06	Glycinin	IY, AG, GS, PQ, YN, AGS, SP, NP, QA, QN, SI	ACE inhibitor, antioxidant, DPP-IV inhibitor
EAFGVNMQIVRNL	1490.74	0.35	0.16	Glycinin	IVR, AF, FG, GV, EA, VR, IV, MQ, NL, NM, QI, RN, VN	ACE inhibitor, glucose uptake stimulation, *α*-glucosidase inhibitor, DPP-IV inhibitor

PQAGSITTATSLDFPA	1576.72	0.23	0.13	Glycinin	FP, AG, GS, ITT, PQ, DF, AGS, SL, PA, TA, AT, QA, SI, TS, TT	ACE inhibitor, DPP-IV inhibitor
LAFPGSAQAVEKLLKNQRES	2186.50	0.21	0.35	Conglycinin	PG, FP, AFP, AF, LA, GS, KL, VE, EK, AV, LL, LK, LAF, ES, NQ, QA	ACE inhibitor, antioxidant, antiamnestic, antithrombotic, glucose uptake stimulation, regulating the stomach mucosal membrane activity, α-glucosidase inhibitor, DPP-IV inhibitor
RSRDPIYSNKLGKF	1680.93	0.65	0.19	Conglycinin	IY, GK, LG, NKL, KF, KL, NK, DP, PI, YS	ACE inhibitor, antioxidant, DPP-IV inhibitor
LAFPGSAQAVEKLLKNQRE	2099.42	0.31	0.41	Conglycinin	PG, FP, AFP, AF, LA, GS, KL, VE, EK, AV, LL, LK, LAF, ES, NQ, GQ, QA	ACE inhibitor, antioxidant, antiamnestic, antithrombotic, glucose uptake stimulation, regulating the stomach mucosal membrane activity, α-glucosidase inhibitor, DPP-IV inhibitor
NQRSPQLQNLRDYRILE	2143.39	0.25	0.29	Conglycinin	LQ, PQ, DY, IL, LR, NLR, YR, RDY, SP, NL, NQ, QL, QN, RI	ACE inhibitor, antioxidant, neuropeptide, glucose uptake stimulation, DPP-IV inhibitor
FAIGINAENNQRN	1460.57	0.22	0.10	Conglycinin	IG, GI, AI, IGINAENNQRN, FA, AE, IN, NA, NN, NQ, RN	ACE inhibitor, antioxidant, DPP-IV inhibitor
QRFNQRSPQLQNLRD	1900.09	0.28	0.44	Conglycinin	RF, FNQ, LQ, PQ, LR, NLR, SP, FN, NL, NQ, QL, QN	ACE inhibitor, DPP-IV inhibitor
YVVNPDNNENLRL	1559.70	0.34	0.18	Conglycinin	RL, VNP, YV, LR, NLR, NEN, VV, NP, DN, NE, NL, NN, RL, VN, YV	ACE inhibitor, antioxidant, DPP-IV inhibitor
PIYSNKLGKF	1166.39	0.77	0.11	Conglycinin	IY, GK, LG, NKL, KF, KL, NK, PI, YS	ACE inhibitor, antioxidant, DPP-IV inhibitor
RLQSGDALRVPSGTTY	1720.90	0.21	0.28	Conglycinin	RL, VP, DA, GT, SG, GD, LQ, LR, TY, VP, AL, PS, QS, TT, TY, RV, DA	ACE inhibitor, antioxidant, DPP-IV inhibitor
ITLAIPVNKPGRF	1425.74	0.57	0.21	Conglycinin	PG, RF, AIP, IP, LA, GR, AI, NK, KP, PV, TL, VN	ACE inhibitor, antioxidant, antiamnestic, antithrombotic, regulating the stomach mucosal membrane activity, DPP-IV inhibitor
RLQSGDALRVPSGT	1456.62	0.27	0.35	Conglycinin	RL, VP, DA, GT, SG, GD, LQ, LR, VP, AL, PS, QS	ACE inhibitor, DPP-IV inhibitor

NEGALLLPHFNSKA	1510.71	0.56	0.16	Conglycinin	LLP, GA, EG, KA, PH, LP, LLL, LL, LLPH, PHF, LP, AL, EG, FN, HF, NE, PH, SK	ACE inhibitor, antioxidant, stimulating vasoactive substance release, glucose uptake stimulation, DPP-IV inhibitor
LSIVDMNEGALLLPHFNSKA	2169.52	0.54	0.13	Conglycinin	LLP, GA, EG, KA, PH, DM, LP, LLL, LL, IV, LLPH, PHF, LP, AL, EG, FN, HF, NE, PH, SK	ACE inhibitor, antioxidant, stimulating vasoactive substance release, glucose uptake stimulation, DPP-IV inhibitor
FNSKPNTLLLPNHADA	1751.96	0.24	0.17	Conglycinin	LLP, DA, KP, LP, LLL, LL, HA, AD, FN, NH, NT, PN, SK, TL	ACE inhibitor, antioxidant, stimulating vasoactive substance release, glucose uptake stimulation, *α*-glucosidase inhibitor, DPP-IV inhibitor
KFFEITPEKNPQLRDLD	2090.36	0.23	0.15	Conglycinin	KF, EI, PQ, EK, TP, LR, FF, KNPQLR, EITPEKNPQLR, NP, EI, QL, PE, LR	ACE inhibitor, fatty acid synthase inhibitor, *α*-glucosidase inhibitor, DPP-IV inhibitor
YRILEFNSKPN	1380.57	0.22	0.12	Conglycinin	KP, LEF, IL, EF, YR, FN, PN, RI, SK	ACE inhibitor, antioxidant, hypolipidemic, neuropeptide, glucose uptake stimulation, DPP-IV inhibitor
FEITPEKNPQLRDLDI	1928.17	0.20	0.12	Conglycinin	EI, PQ, EK, TP, LR, KNPQLR, EITPEKNPQLR, NP, EI, QL, PE, LR	ACE inhibitor, fatty acid synthase inhibitor, *α*-glucosidase inhibitor, DPP-IV inhibitor
ITLAIPVNKPGRFESF	1789.11	0.40	0.13	Conglycinin	PG, RF, AIP, IP, LA, GR, AI, NK, KP, PV, TL, VN	ACE inhibitor, antioxidant, antiamnestic, antithrombotic, regulating the stomach mucosal membrane activity, DPP-IV inhibitor
LAFPGSAQAVEKLLK	1571.88	0.40	0.39	Conglycinin	PG, FP, AFP, AF, LA, GS, KL, VE, EK, AV, LL, LK, LAF, ES, NQ, GQ, QA	ACE inhibitor, antioxidant, antiamnestic, antithrombotic, glucose uptake stimulation, regulating the stomach mucosal membrane activity, *α*-glucosidase inhibitor, DPP-IV inhibitor
FEITPEKNPQLR	1471.68	0.31	0.17	Conglycinin	EI, PQ, EK, TP, LR, KNPQLR, EITPEKNPQLR, NP, EI, QL, PE	ACE inhibitor, fatty acid synthase inhibitor, *α*-glucosidase inhibitor, DPP-IV inhibitor
VLQRFNQRSPQLQNLRD	2112.38	0.20	0.49	Conglycinin	RF, FNQ, LQ, PQ, LR, NLR, VL, SP, FN, NL, NQ, QL, QN	ACE inhibitor, glucose uptake stimulation, DPP-IV inhibitor
FNSKPNTLLLPNHADAD	1867.05	0.23	0.15	Conglycinin	LLP, DA, KP, LP, LL, LLL, HA, AD, FN, NH, NT, PN, SK, TL	ACE inhibitor, antioxidant, stimulating vasoactive substance release, glucose uptake stimulation, *α*-glucosidase inhibitor, DPP-IV inhibitor
FEITPEKNPQLRDLDIF	2075.35	0.35	0.11	Conglycinin	IF, EI, PQ, EK, TP, LR, KNPQLR, EITPEKNPQLR, NP, EI, QL, PE	ACE inhibitor, fatty acid synthase inhibitor, *α*-glucosidase inhibitor, DPP-IV inhibitor

GALLLPHFNS	1068.24	0.49	0.13	Conglycinin	LLP, GA, PH, LP, LLL, LL, LLPH, PHF, AL, FN, HF	ACE inhibitor, antioxidant, stimulating vasoactive substance release, glucose uptake stimulation, DPP-IV inhibitor
AELSEQDIFVIPA	1431.61	0.23	0.07	Conglycinin	IPA, IP, IF, AEL, SE, EL, PA, AE, QD, VI	ACE inhibitor, antioxidant, stimulating vasoactive substance release, DPP-IV inhibitor
RAELSEQDIFVIPAGYPV	2004.27	0.24	0.10	Conglycinin	IPA, GY, YP, IP, RA, IF, AG, AEL, SE, VIPAGYP, EL, PAGY, PA, AE, PV, QD, VI	ACE inhibitor, antioxidant, stimulating vasoactive substance release, α-glucosidase inhibitor, DPP-IV inhibitor
YRILEFNSKPNT	1481.67	0.20	0.13	Conglycinin	KP, LEF, IL, EF, YP, FN, NT, PN, RI, SK	ACE inhibitor, antioxidant, hypolipidemic, neuropeptide, glucose uptake stimulation, DPP-IV inhibitor
LLLPHFNSKA	1139.36	0.39	0.21	Conglycinin	LLP, KA, PH, LP, LLL, LL, LLPH, PHF, FN, HF, SK	ACE inhibitor, antioxidant, stimulating vasoactive substance release, glucose uptake stimulation, DPP-IV inhibitor
RSRDPIYSNKLG	1405.58	0.42	0.17	Conglycinin	IY, LG, NKL, KL, NK, IY, DP, PI, YS	ACE inhibitor, antioxidant, DPP-IV inhibitor
ITLAIPVNKPGRFES	1641.93	0.23	0.15	Conglycinin	PG, RF, AIP, IP, LA, GR, AI, NK, KP, PV, ES, TL, VN	ACE inhibitor, antioxidant, antiamnestic, antithrombotic, regulating the stomach mucosal membrane activity, DPP-IV inhibitor
MNEGALLLPHF	1241.47	0.57	0.13	Conglycinin	LLP, GA, EG, PH, LP, LLL, LL, LLPH, PHF, HF, MN, NE	ACE inhibitor, antioxidant, stimulating vasoactive substance release, glucose uptake stimulation, DPP-IV inhibitor
YVVNPDNNENLRLIT	1773.96	0.21	0.16	Conglycinin	RL, VNP, YV, LR, NLR, LI, NEN, VV, NP, DN, NE, NL, NN, VN	ACE inhibitor, antioxidant, glucose uptake stimulation, DPP-IV inhibitor
FAIGINAENNQRNFL	1720.91	0.43	0.11	Conglycinin	IG, GI, AI, NF, IGINAENNQRN, FA, FL, AE, IN, NA, NN, NQ, RN	ACE inhibitor, antioxidant, DPP-IV inhibitor
NLRSRDPIYSNKLG	1632.84	0.25	0.19	Conglycinin	IY, LG, NKL, KL, NK, LR, NLR, DP, NL, PI, YS	ACE inhibitor, antioxidant, DPP-IV inhibitor
LSIVDMNEGALLLPH	1621.91	0.39	0.12	Conglycinin	LLP, GA, EG, PH, DM, LP, LLL, IV, LL, LLPH, AL, EG, MN, NE, SI, VD	ACE inhibitor, antioxidant, stimulating vasoactive substance release, glucose uptake stimulation, DPP-IV inhibitor
LRSRDPIYSNKLG	1518.74	0.30	0.20	Conglycinin	IY, LG, NKL, KL, NK, LR, DP, PI, YS	ACE inhibitor, antioxidant, DPP-IV inhibitor

LAIPVNKPGRFES	1427.67	0.42	0.16	Conglycin in	PG, RF, AIP, IP, LA, GR, AI, NK, KP, PV, ES, VN	ACE inhibitor, antioxidant, antiamnestic, antithrombotic, regulating the stomach mucosal membrane activity, DPP-IV inhibitor
SIVDMNEGALLLPH	1508.75	0.51	0.10	Conglycin in	LLP, GA, EG, PH, DM, LP, LLL, IV, LL, LLPH, AL, EG, MN, NE, SI, VD	ACE inhibitor, antioxidant, stimulating vasoactive substance release, glucose uptake stimulation, DPP-IV inhibitor
FNSKPNTLLLPNHADA DYL	2143.38	0.39	0.15	Conglycin in	YL, LLP, DA, KP, DY, LP, LLL, LL, YL, DY, HA, AD, FN, NH, NT, PN, SK, TL	ACE inhibitor, antioxidant, neuropeptide, stimulating vasoactive substance release, glucose uptake stimulation, α-glucosidase inhibitor, DPP-IV inhibitor
FEITPEKNPQLRD	1586.76	0.22	0.14	Conglycin in	EI, PQ, EK, TP, LR, KNPQLR, EITPEKNPQLR, NP, QL, PE	ACE inhibitor, fatty acid synthase inhibitor, α-glucosidase inhibitor, DPP-IV inhibitor
ISKEQIRALSKRA	1499.78	0.22	0.33	Conglycin in	IR, IRA, KR, RA, KE, AL, QI, SK	ACE inhibitor, antioxidant, DPP-IV inhibitor
RLQSGDALRVPSGTT	1557.73	0.23	0.37	Conglycin in	RL, VP, DA, GT, SG, GD, LQ, LR, VP, AL, PS, QS, TT	ACE inhibitor, DPP-IV inhibitor
EITPEKNPQLRDL	1552.75	0.20	0.20	Conglycin in	EI, PQ, EK, TP, LR, KNPQLR, EITPEKNPQLR, NP, QL, PE	ACE inhibitor, fatty acid synthase inhibitor, α-glucosidase inhibitor, DPP-IV inhibitor
QGFSRNILEA	1134.26	0.32	0.11	Conglycin in	GF, QG, EA, IL, RN	ACE inhibitor, glucose uptake stimulation, α-glucosidase inhibitor, DPP-IV inhibitor
SRDPIYSNKLG	1249.39	0.53	0.11	Conglycin in	IY, LG, NKL, KL, NK, DP, PI, YS	ACE inhibitor, antioxidant, DPP-IV inhibitor
AIPVNKPGRF	1098.31	0.85	0.21	Conglycin in	PG, RF, AIP, IP, GR, AI, NK, KP, PV, VN	ACE inhibitor, antioxidant, antiamnestic, antithrombotic, regulating the stomach mucosal membrane activity, DPP-IV inhibitor
SEQDIFVIPA	1118.25	0.39	0.06	Conglycin in	IPA, IP, IF, SE, PA, QD, VI	ACE inhibitor, stimulating vasoactive substance release, DPP-IV inhibitor
FLSSTEAQQSYLQGFSR NIL	2289.53	0.32	0.11	Conglycin in	YL, GF, QG, EA, SY, TE, LQ, IL, ST, FL, QQ, QS, RN, TE	ACE inhibitor, neuropeptide, glucose uptake stimulation, α-glucosidase inhibitor, DPP-IV inhibitor

LRSRDPIYSNKLGK	1646.91	0.32	0.27	Conglycinin	IY, GK, LG, NKL, KL, NK, LR, DP, PI, YS	ACE inhibitor, antioxidant, DPP-IV inhibitor
EDEQPRPIPFPRPQPR	1959.19	0.62	0.21	Conglycinin	FP, PR, IP, RP, PQ, PFP, QP, PI	ACE inhibitor, DPP-IV inhibitor
MNEGALLLPHFNSKA	1641.91	0.41	0.16	Conglycinin	LLP, GA, EG, KA, PH, LP, LLL, LL, LLPH, PHF, AL, EG, MN, NE	ACE inhibitor, antioxidant, stimulating vasoactive substance release, glucose uptake stimulation, DPP-IV inhibitor
NEGALLLPHF	1110.28	0.71	0.14	Conglycinin	LLP, GA, EG, PH, LP, LLL, LL, LLPH, PHF, AL, EG, NE	ACE inhibitor, antioxidant, stimulating vasoactive substance release, glucose uptake stimulation, DPP-IV inhibitor
FNQRSPQLQNLRD	1615.77	0.27	0.28	Conglycinin	FNQ, LQ, PQ, LR, NLR, SP, FN, NL, NQ, QL, QN	ACE inhibitor, DPP-IV inhibitor
KTISSEDKPFNLRSRD	1893.09	0.21	0.15	Conglycinin	KP, LR, NLR, SE, FN, KT, NL, PF, TI	ACE inhibitor, antioxidant, stimulating vasoactive substance release, DPP-IV inhibitor
TISSEDKPFN	1137.21	0.26	0.06	Conglycinin	KP, SE, FN, PF, TI	ACE inhibitor, antioxidant, stimulating vasoactive substance release, DPP-IV inhibitor
NSKPNTLLLPNHADADYL	1996.21	0.32	0.17	Conglycinin	YL, LLP, DA, KP, DY, LP, LLL, LL, HA, AD, NH, NT, PN, SK, TL	ACE inhibitor, antioxidant, neuropeptide, stimulating vasoactive substance release, glucose uptake stimulation, α-glucosidase inhibitor, DPP-IV inhibitor
LAFPGSAQAVEK	1217.39	0.22	0.19	Conglycinin	PG, FP, AFP, AF, LA, GS, VE, EK, AV, LAF, QA	ACE inhibitor, antioxidant, antiamnestic, antithrombotic, regulating the stomach mucosal membrane activity, α-glucosidase inhibitor, DPP-IV inhibitor
FLSSTEAQQSYLQG	1558.66	0.21	0.09	Conglycinin	YL, QG, EA, SY, TE, LQ, ST, FL, QQ, QS	ACE inhibitor, neuropeptide, α-glucosidase inhibitor, DPP-IV inhibitor
NSKPNTLLLPNHADADY	1883.05	0.20	0.15	Conglycinin	LLP, DA, KP, DY, LP, LLL, LL, HA, AD, NH, NT, PN, SK, TL	ACE inhibitor, antioxidant, stimulating vasoactive substance release, glucose uptake stimulation, α-glucosidase inhibitor, DPP-IV inhibitor
ESEDSELRRHKNKNPF	1986.13	0.42	0.17	Conglycinin	NK, RR, HK, LR, SE, EL, RHK, NP, ES, PF	ACE inhibitor, antioxidant, stimulating vasoactive substance release, DPP-IV inhibitor

NQRSPQLQNL	1197.32	0.26	0.30	Conglycin in	LQ, PQ, SP, NL, NQ, PQ, QL, QN	ACE inhibitor, DPP-IV inhibitor
VDMNEGALLLPH	1308.52	0.28	0.15	Conglycin in	LLP, GA, EG, PH, DM, LP, LLL, LL, LLPH, AL, MN, NE, VD	ACE inhibitor, antioxidant, stimulating vasoactive substance release, glucose uptake stimulation, DPP-IV inhibitor
FAIGINAENNQRNF	1607.75	0.37	0.09	Conglycin in	IG, GI, AI, NF, IGINAENNQRN, FA, AE, IN, NA, NN, NQ, RN	ACE inhibitor, antioxidant, DPP-IV inhibitor
LAIPVNKPGRFESF	1574.84	0.64	0.13	Conglycin in	PG, RF, AIP, IP, LA, GR, AI, NK, KP, ES, PV, VN	ACE inhibitor, antioxidant, antiamnestic, antithrombotic, regulating the stomach mucosal membrane activity, DPP-IV inhibitor
NEGALLLPHFNS	1311.46	0.41	0.11	Conglycin in	LLP, GA, EG, PH, DM, LP, LLL, LL, LLPH, PHF, AL, NE	ACE inhibitor, antioxidant, stimulating vasoactive substance release, glucose uptake stimulation, DPP-IV inhibitor
RDLDIF	777.88	0.62	0.10	Conglycin in	IF	ACE inhibitor
FGSNRFET	957.01	0.46	0.08	Conglycin in	RF, FG, GS, ET, NR	ACE inhibitor, DPP-IV inhibitor
SIVDMNEGALLLPHFNS KA	2056.36	0.65	0.11	Conglycin in	LLP, GA, EG, KA, PH, DM, LP, LLL, IV, LL, LLPH, PHF, AL, FN, HF, MN, NE, SI, SK, VD	ACE inhibitor, antioxidant, stimulating vasoactive substance release, glucose uptake stimulation, DPP-IV inhibitor
FKNQYGRIRV	1280.50	0.37	0.28	Conglycin in	IR, YG, GR, NQ, QY, RI, RV	ACE inhibitor, antioxidant, antiinflammation, DPP-IV inhibitor
LSIVDMNEGALLLPHFN S	1970.27	0.39	0.09	Conglycin in	LLP, GA, EG, PH, DM, LP, LLL, IV, LL, LLPH, PHF, AL, FN, HF, MN, NE, SI, SK, VD	ACE inhibitor, antioxidant, stimulating vasoactive substance release, glucose uptake stimulation, DPP-IV inhibitor
NEGALLLPHFNSKAI	1623.87	0.64	0.13	Conglycin in	LLP, GA, AI, EG, KA, PH, LP, LLL, LL, LLPH, PHF, KAI, AL, FN, HF, NE, SK	ACE inhibitor, antioxidant, stimulating vasoactive substance release, glucose uptake stimulation, DPP-IV inhibitor
ILEFNSKPNT	1162.31	0.22	0.09	Conglycin in	KP, LEF, IL, EF, FN, IL, NT, PN, SK, EF	ACE inhibitor, antioxidant, hypolipidemic, glucose uptake stimulation, DPP-IV inhibitor
RDLDIFL	891.03	0.70	0.13	Conglycin in	IF, IFL, FL	ACE inhibitor, DPP-IV inhibitor

QRFNQRSPQLQNL	1628.81	0.37	0.39	Conglycinin	RF, FNQ, LQ, PQ, SP, FN, NL, NQ, QL, QN	ACE inhibitor, DPP-IV inhibitor
IPVNKPGRF	1027.23	0.81	0.19	Conglycinin	PG, RF, IP, GR, NK, KP, PV, VN	ACE inhibitor, antioxidant, antiamnestic, antithrombotic, regulating the stomach mucosal membrane activity, DPP-IV inhibitor
NEGALLLPH	963.10	0.38	0.19	Conglycinin	LLP, GA, EG, PH, LP, LLL, LL, LLPH, AL, NE	ACE inhibitor, antioxidant, stimulating vasoactive substance release, glucose uptake stimulation, DPP-IV inhibitor
PIYSNKLG	891.03	0.48	0.09	Conglycinin	IY, LG, NKL, KL, NK, PI, YS	ACE inhibitor, antioxidant, DPP-IV inhibitor
FAIGINAENNQRNFLAGSQ	2064.24	0.20	0.11	Conglycinin	LA, IG, GI, AG, GS, AI, NF, AGS, FA, FL, AE, IN, NA, IGINAENNQRN, NN, NQ, RN	ACE inhibitor, antioxidant, DPP-IV inhibitor
FAIGINAENNQRNFLAG	1849.04	0.38	0.11	Conglycinin	LA, IG, GI, AG, AI, NF, IGINAENNQRN, FA, FL, AE, IN, NA, NN, NQ, RN	ACE inhibitor, antioxidant, DPP-IV inhibitor
VVNPDNNENLRLIT	1610.79	0.27	0.19	Conglycinin	RL, VNP, LR, NLR, LI, NEN, VV, NP, DN, NE, NL, NN, VN	ACE inhibitor, antioxidant, glucose uptake stimulation, DPP-IV inhibitor
EEINKVLF	991.15	0.23	0.11	Conglycinin	LF, NK, EI, INKV, VL, EE, IN, KV	ACE inhibitor, stimulating vasoactive substance release, glucose uptake stimulation, DPP-IV inhibitor
LAIPVNKPG	908.11	0.35	0.22	5Conglycinin	PG, AIP, IP, LA, AI, NK, KP, IP, PV, VN	ACE inhibitor, antioxidant, antiamnestic, antithrombotic, regulating the stomach mucosal membrane activity, DPP-IV inhibitor
AIPVNKPGRFES	1314.51	0.50	0.13	Conglycinin	PG, RF, AIP, IP, DR, AI, NK, KP, IP, ES, PV, VN	ACE inhibitor, antioxidant, antiamnestic, antibacterial, regulating the stomach mucosal membrane activity, DPP-IV inhibitor
SEQDIFVIPAG	1175.30	0.42	0.05	Conglycinin	IPA, IP, IF, AG, SE, PA, QD, VI	ACE inhibitor, stimulating vasoactive substance release, DPP-IV inhibitor
SSSRKTISSEDKPFN	1682.81	0.25	0.09	Conglycinin	KP, SSS, SE, FN, KT, PF, RK, TI	ACE inhibitor, antioxidant, stimulating vasoactive substance release, DPP-IV inhibitor
FSRNILE	878.00	0.36	0.10	Conglycinin	IL, RN	ACE inhibitor, glucose uptake stimulation, DPP-IV inhibitor

LPNHADADYL	1128.21	0.32	0.11	Conglycin in	YL, DA, DY, LP, HA, AD, NH, PN	ACE inhibitor, neuropeptide, α-glucosidase inhibitor, DPP-IV inhibitor
LRLIT	614.79	0.20	0.61	Conglycin in	RL, LR, LI	ACE inhibitor, stimulating vasoactive substance release, DPP-IV inhibitor
QGFSRNILE	1063.18	0.39	0.10	Conglycin in	GF, QG, IL, RN	ACE inhibitor, glucose uptake stimulation, DPP-IV inhibitor
LAIPVNKPGRFESFF	1722.02	0.78	0.11	Conglycin in	PG, RF, AIP, IP, LA, GR, AI, SF, NK, KP, FF, IP, ES, PV, VN	ACE inhibitor, antioxidant, antiamnestic, antithrombotic, regulating the stomach mucosal membrane activity, DPP-IV inhibitor
FEITPEKNPQL	1315.49	0.23	0.11	Conglycin in	EI, PQ, EK, TP, NP, QL, PE	ACE inhibitor, α-glucosidase inhibitor, DPP-IV inhibitor
SPQLQNLRD	1070.17	0.33	0.23	Conglycin in	LQ, PQ, LR, NLR, SP, NL, QL, QN	ACE inhibitor, DPP-IV inhibitor
FSRNIL	748.88	0.65	0.12	Conglycin in	IL, RN	ACE inhibitor, glucose uptake stimulation, DPP-IV inhibitor
FEITPEKNPQLRDLDIFL	2188.51	0.46	0.13	Conglycin in	IF, EI, PQ, EK, IFL, TP, LR, KNPQLR, EITPEKNPQLR, NP, FL, QL, PE	ACE inhibitor, fatty acid synthase inhibitor, α-glucosidase inhibitor, DPP-IV inhibitor
LAFPGSAQ	789.89	0.30	0.11	Conglycin in	PG, FP, AFP, AF, LA, GS, LAF	ACE inhibitor, antioxidant, antiamnestic, antithrombotic, regulating the stomach mucosal membrane activity, DPP-IV inhibitor
ITLAIPVNKPGRFESFF	1936.28	0.56	0.11	Conglycin in	PG, RF, AIP, IP, LA, GR, AI, SF, NK, KP, FF, IP, ES, PV, VN	ACE inhibitor, antioxidant, antiamnestic, antithrombotic, regulating the stomach mucosal membrane activity, DPP-IV inhibitor
FFEITPEKNPQLRDLD	1962.19	0.25	0.12	Conglycin in	EI, PQ, EK, TP, LR, FF, KNPQLR, EITPEKNPQLR, NP, QL, PE	ACE inhibitor, fatty acid synthase inhibitor, α-glucosidase inhibitor, DPP-IV inhibitor
SKPNTLLLPNHADAD	1605.77	0.20	0.18	Conglycin in	LLP, DA, KP, LP, LLL, LL, HA, AD, NH, NT, PN, TL	ACE inhibitor, antioxidant, stimulating vasoactive substance release, glucose uptake stimulation, α-glucosidase inhibitor, DPP-IV inhibitor
GALLLPH	719.88	0.54	0.33	Conglycin in	LLP, GA, PH, LP, LLL, LL, LLPH, AL	ACE inhibitor, antioxidant, stimulating vasoactive substance release, glucose uptake stimulation, DPP-IV inhibitor

SEQDIFVIPAGY	1338.48	0.38	0.05	Conglycinin	IPA, GY, IP, IF, AG, SE, PAGY, PA, QD, VI	ACE inhibitor, antioxidant, stimulating vasoactive substance release, DPP-IV inhibitor
EITPEKNPQLRDLDIF	1928.17	0.30	0.12	Conglycinin	IF, EI, PQ, EK, TP, LR, KNPQLR, EITPEKNPQLR, NP, QL, PE	ACE inhibitor, fatty acid synthase inhibitor, *α*-glucosidase inhibitor, DPP-IV inhibitor
LAFPGSAQAVEKLLKNQ	1814.11	0.28	0.37	Conglycinin	PG, FP, AFP, AF, LA, GS, VE, EK, AV, LAF, NQ, QA	ACE inhibitor, antioxidant, antiamnestic, antithrombotic, glucose uptake stimulation, regulating the stomach mucosal membrane activity, *α*-glucosidase inhibitor, DPP-IV inhibitor
PVNKPGRF	914.08	0.80	0.30	Conglycinin	PG, RF, GR, NK, KP, PV, VN	ACE inhibitor, antioxidant, antiamnestic, antithrombotic, regulating the stomach mucosal membrane activity, DPP-IV inhibitor
SEQDIFVIPAGYPV	1534.73	0.44	0.06	Conglycinin	IPA, GY, YP, IP, IF, AG, SE, VIPAGYP, PAGY, PA, IPA, PV, QD, VI	ACE inhibitor, antioxidant, stimulating vasoactive substance release, *α*-glucosidase inhibitor, DPP-IV inhibitor
LLLPH	591.75	0.41	0.40	Conglycinin	LLP, PH, LP, LLL, LL, LLPH	ACE inhibitor, antioxidant, stimulating vasoactive substance release, glucose uptake stimulation, DPP-IV inhibitor
FGSNRFETLFKNQ	1587.76	0.33	0.15	Conglycinin	RF, LF, FG, GS, ET, NQ, NR, TL	ACE inhibitor, DPP-IV inhibitor
LLLPHFNS	940.11	0.36	0.12	Conglycinin	LLP, PH, LP, LLL, LL, LLPH, PHF, FN, HF	ACE inhibitor, antioxidant, stimulating vasoactive substance release, glucose uptake stimulation, DPP-IV inhibitor
PNHADADYLI	1128.21	0.42	0.07	Conglycinin	YL, DA, DY, LI, HA, AD, NH, PN	ACE inhibitor, neuropeptide, glucose uptake stimulation, *α*-glucosidase inhibitor, DPP-IV inhibitor
GALLLPHFNSKA	1267.49	0.61	0.21	Conglycinin	LLP, GA, KA, PH, LP, LLL, LL, LLPH, PHF, AL, SK, FN, HF	ACE inhibitor, antioxidant, stimulating vasoactive substance release, glucose uptake stimulation, DPP-IV inhibitor
NSKPNTLLLPN	1210.40	0.34	0.28	Conglycinin	LLP, KP, LP, LLL, LL, NT, PN, SK, TL	ACE inhibitor, antioxidant, stimulating vasoactive substance release, glucose uptake stimulation, DPP-IV inhibitor
YRILEF	839.99	0.44	0.11	Conglycinin	LEF, IL, EF, YR, RI	ACE inhibitor, hypolipidemic, neuropeptide, glucose uptake stimulation, DPP-IV inhibitor

LSIVDMNEGAL	1161.34	0.31	0.09	Conglycin in	GA, EG, DM, IV, AL, MN, NE, SI, VD	ACE inhibitor, glucose uptake stimulation, DPP-IV inhibitor
LLPHFNSKA	1026.20	0.39	0.17	Conglycin in	LLP, KA, PH, LP, LL, LLPH, PHF, SK, FN, HF	ACE inhibitor, antioxidant, glucose uptake stimulation, DPP-IV inhibitor
SSSRKTISSEDKPFNLRS	2039.23	0.25	0.12	Conglycin in	KP, LR, NLR, SSS, SE, FN, KT, NL, PF, RK, TI	ACE inhibitor, antioxidant, stimulating vasoactive substance release, DPP-IV inhibitor
IFVIPAGYPV	1075.32	0.38	0.07	Conglycin in	IPA, GY, YP, IP, IF, AG, VIPAGYP, PAGY, PA, AG, PV, VI	ACE inhibitor, antioxidant, α-glucosidase inhibitor, DPP-IV inhibitor
KNQYGRIRVLQ	1374.61	0.20	0.47	Conglycin in	IR, YG, GR, LQ, VL, NQ, QY, RI, RV	ACE inhibitor, antioxidant, antiinflammation, glucose uptake stimulation, DPP-IV inhibitor
SEQDIFVIPAGYPVVV	1732.99	0.52	0.07	Conglycin in	IPA, GY, YP, IP, IF, AG, SE, VVV, VIPAGYP, PAGY, PA, VV, AG, PV, QD, VI	ACE inhibitor, antioxidant, anticancer, stimulating vasoactive substance release, α-glucosidase inhibitor, DPP-IV inhibitor
LAFPGSAQA	860.97	0.28	0.13	Conglycin in	PG, FP, AFP, AF, LA, GS, LAF, QA	ACE inhibitor, antioxidant, antiamnestic, antithrombotic, regulating the stomach mucosal membrane activity, DPP-IV inhibitor
QLQNLRDYRILEF	1707.95	0.42	0.24	Conglycin in	LQ, LEF, DY, IL, LR, NLR, EF, YR, RDY, NL, QL, QN, RI, YR	ACE inhibitor, antioxidant, hypolipidemic, neuropeptide, glucose uptake stimulation, DPP-IV inhibitor
LLPHFNS	826.95	0.46	0.09	Conglycin in	LLP, PH, LP, LL, LLPH, PHF, SK, FN, HF	ACE inhibitor, antioxidant, glucose uptake stimulation, DPP-IV inhibitor
IPSQVQELAFPGS	1372.54	0.33	0.08	Conglycin in	PG, FP, AFP, IP, AF, LA, GS, EL, LAF, PS, QE, QV, VQ	ACE inhibitor, antioxidant, antiamnestic, antithrombotic, regulating the stomach mucosal membrane activity, DPP-IV inhibitor
LLLPHF	738.93	0.77	0.21	Conglycin in	LLP, PH, LP, LLL, LL, LLPH, PHF, SK, FN, HF	ACE inhibitor, antioxidant, stimulating vasoactive substance release, glucose uptake stimulation, DPP-IV inhibitor
SQIPSQVQEL	1128.25	0.21	0.08	Conglycin in	IP, EL, PS, QE, QI, QV, VQ	ACE inhibitor, antioxidant, DPP-IV inhibitor
EASYDTKF	960.01	0.41	0.07	Conglycin in	EA, SY, KF, AS, TK, YD	ACE inhibitor, DPP-IV inhibitor

DEQPRPIPFPRPQPR	1830.08	0.73	0.25	Conglycinin	FP, PR, IP, RP, PQ, PFP, QP, PF, PI	ACE inhibitor, DPP-IV inhibitor
KKEEGNKGRKGPLS	1527.74	0.28	0.41	Conglycinin	GP, GPL, PL, GR, KG, EG, NK, KE, KGP, EE, KK	ACE inhibitor, antiamnestic, antithrombotic, stimulating vasoactive substance release, regulating the stomach mucosal membrane activity, DPP-IV inhibitor
AENNQRNFLAG	1233.31	0.36	0.19	Conglycinin	LA, AG, NF, FL, AE, NN, NQ, RN	ACE inhibitor, DPP-IV inhibitor
YRILEFN	954.09	0.31	0.11	Conglycinin	LEF, IL, EF, YR, FN, RI	ACE inhibitor, hypolipidemic, neuropeptide, glucose uptake stimulation, DPP-IV inhibitor
SIVDMNEGALL	1161.34	0.50	0.09	Conglycinin	GA, EG, DM, IV, LL, AL, MN, NE, SI, VD	ACE inhibitor, glucose uptake stimulation, DPP-IV inhibitor
KLQSPDDERKQIVTVEGGL	2112.37	0.23	0.20	Glycinin	GL, GG, EG, KL, VE, LQ, ER, IV, SP, QI, QS, RK, TV, VT	ACE inhibitor, glucose uptake stimulation, α-glucosidase inhibitor, DPP-IV inhibitor
NRNGLHLPSYSPYPR	1770.97	0.36	0.16	Glycinin	LHLP, PYP, YPR, PR, YP, PSY, GL, HL, NG, SY, LP, LH, LHL, PY, NR, PS, RN, YS	ACE inhibitor, antioxidant, antiinflammation, α-glucosidase inhibitor, DPP-IV inhibitor
KYEGNWGPLVNPESQQGSPR	2243.42	0.34	0.15	Glycinin	GP, PR, GPL, PL, NWGPLV, GS, WG, QG, EG, KY, VNP, YE, LV, VNPESQQGSPR, NP, ES, NW, QG, QQ, VN, PE	ACE inhibitor, antioxidant, antiamnestic, antithrombotic, glucose uptake stimulation, regulating the stomach mucosal membrane activity, α-glucosidase inhibitor, DPP-IV inhibitor
SGFSKHFLAQSFNTNE	1813.94	0.43	0.07	Glycinin	LA, GF, SG, SF, FL, FN, HF, KH, NE, NT, QS, SK, TN	ACE inhibitor, DPP-IV inhibitor
LKYEGNWGPLVNPESQQGSP	2200.39	0.24	0.12	Glycinin	GP, GPL, PL, NWGPLV, GS, WG, QG, EG, KY, VNP, YE, LV, VNPESQQGSPR, NP, ES, NW, QG, QQ, VN, PE	ACE inhibitor, antioxidant, antiamnestic, antithrombotic, glucose uptake stimulation, regulating the stomach mucosal membrane activity, α-glucosidase inhibitor, DPP-IV inhibitor
TLNRNGLHLPSYSPYPRM	2116.43	0.37	0.18	Glycinin	LHLP, PYP, YPR, PR, YP, PSY, GL, HL, NG, SY, LP, LH, LHL, PY, NR, PS, RN, YS	ACE inhibitor, antioxidant, antiinflammation, α-glucosidase inhibitor, DPP-IV inhibitor

KYEGNWGPLVNPESQQGS	1990.12	0.24	0.11	Glycinin	GP, GPL, PL, NWGPLV, GS, WG, QG, EG, KY, VNP, YE, LV, VNPESQQGSPR, NP, ES, NW, QG, QQ, VN, PE	ACE inhibitor, antioxidant, antiamnestic, antithrombotic, glucose uptake stimulation, regulating the stomach mucosal membrane activity, α-glucosidase inhibitor, DPP-IV inhibitor
KYEGNWGPLVNPESQQGSP	2087.23	0.27	0.11	Glycinin	GP, GPL, PL, NWGPLV, GS, WG, QG, EG, KY, VNP, YE, LV, VNPESQQGSPR, NP, ES, NW, QG, QQ, VN, PE	ACE inhibitor, antioxidant, antiamnestic, antithrombotic, glucose uptake stimulation, regulating the stomach mucosal membrane activity, α-glucosidase inhibitor, DPP-IV inhibitor
ADFYNPKAGRIST	1439.59	0.46	0.13	Glycinin	FY, AG, GR, KA, FYN, DF, ST, YN, DFY, ADF, NP, AD, PK, RI	ACE inhibitor, antioxidant, α-glucosidase inhibitor, DPP-IV inhibitor
LKYEGNWGPLVNPESQQG	2016.20	0.22	0.14	Glycinin	GP, GPL, GP, PL, NWGPLV, GS, WG, QG, EG, KY, VNP, YE, LV, VNPESQQGSPR, NP, ES, NW, QG, QQ, VN, PE	ACE inhibitor, antioxidant, antiamnestic, antithrombotic, glucose uptake stimulation, regulating the stomach mucosal membrane activity, α-glucosidase inhibitor, DPP-IV inhibitor
TLNRNGLHLPSYSPYPR	1985.23	0.25	0.19	Glycinin	LHLP, PYP, YPR, PR, YP, PSY, GL, HL, NG, SY, LP, LH, LHL, PY, NR, PS, RN, YS	ACE inhibitor, antioxidant, antiinflammation, α-glucosidase inhibitor, DPP-IV inhibitor
NRNGLHLPSYSPYP	1614.78	0.28	0.11	Glycinin	LHLP, PYP, YPR, YP, PSY, GL, HL, NG, SY, LP, LH, LHL, PY, NR, PS, RN, YS	ACE inhibitor, antioxidant, antiinflammation, α-glucosidase inhibitor, DPP-IV inhibitor
NLNALEPDHRVESEGGL	1849.97	0.25	0.13	Glycinin	GL, GG, EG, VE, LN, ALEP, SE, ALEPDHR, NALEPDHRVESEGG, EP, AL, ES, HR, NA, NL, VE	ACE inhibitor, antioxidant, stimulating vasoactive substance release, α-glucosidase inhibitor, DPP-IV inhibitor
NRNGLHLPSYSPYPRM	1902.16	0.52	0.15	Glycinin	LHLP, PYP, YPR, YP, PSY, GL, HL, NG, SY, LP, LH, LHL, PY, NR, PS, RN, YS	ACE inhibitor, antioxidant, antiinflammation, α-glucosidase inhibitor, DPP-IV inhibitor
RADFYNPKAGRIST	1595.78	0.33	0.20	Glycinin	FY, RA, AG, GR, KA, FYN, DF, ST, YN, DFY, RADFY, ADF, NP, AD, PK, RI, YN	ACE inhibitor, antioxidant, α-glucosidase inhibitor, DPP-IV inhibitor
HENIARPSRADFYNPKAG	2043.23	0.24	0.15	Glycinin	FY, IA, RP, RA, AG, AR, GR, KA, FYN, DF, YN, DFY, RADFY, ADF, NP, AD, PK, RI, YN, HE, PS	ACE inhibitor, antioxidant, α-glucosidase inhibitor, DPP-IV inhibitor
RAIPSEVLAHSYNL	1569.78	0.25	0.13	Glycinin	AIP, AHSY, IP, LA, RA, AI, SY, EV, AH, YN, YNL, SE, HS, NL, PS, SY, VL	ACE inhibitor, antioxidant, stimulating vasoactive substance release, glucose uptake stimulation, DPP-IV inhibitor

KYEGNWGPLVNPESQQG	1903.04	0.25	0.12	Glycinin	GP, GPL, GP, PL, NWGPLV, GS, WG, QG, EG, KY, VNP, YE, LV, VNPESQQGSPR, NP, ES, NW, QG, QQ, VN, PE	ACE inhibitor, antioxidant, antiamnestic, antithrombotic, glucose uptake stimulation, regulating the stomach mucosal membrane activity, α-glucosidase inhibitor, DPP-IV inhibitor
FNNQLDQTPR	1232.32	0.30	0.20	Glycinin	PR, TP, DQ, FN, NN, NQ, QL, QT	ACE inhibitor, DPP-IV inhibitor
TLNRNGLHLPSYSPYP	1829.05	0.20	0.14	Glycinin	LHLP, PYP, YPR, YP, PSY, GL, HL, NG, SY, LP, LH, LHL, PY, NR, PS, RN	ACE inhibitor, antioxidant, antiinflammation, α-glucosidase inhibitor, DPP-IV inhibitor
WGPLVNPESQQGSPR	1651.80	0.40	0.16	Glycinin	GP, PR, GPL, PL, GS, WG, QG, VNP, LV, VNPESQQGSPR, NP, ES, QG, QQ, VN, PE	ACE inhibitor, antioxidant, antiamnestic, antithrombotic, glucose uptake stimulation, regulating the stomach mucosal membrane activity, α-glucosidase inhibitor, DPP-IV inhibitor
IQTWNSQHPEL	1352.47	0.23	0.10	Glycinin	HP, EL, PEL, TW, WN, IQ, QH, QT, PE	ACE inhibitor, antioxidant, α-glucosidase inhibitor, DPP-IV inhibitor
PSYSPYPR	966.06	0.81	0.08	Glycinin	PYP, YPR, PR, YP, PSY, SY, PY, SP, PS, YS	ACE inhibitor, antiinflammation, α-glucosidase inhibitor, DPP-IV inhibitor
NSLTLPALRQFQLSAQ	1787.05	0.24	0.38	Glycinin	FQ, LR, LP, SL, PA, AL, LT, QF, QL, TL	ACE inhibitor, α-glucosidase inhibitor, DPP-IV inhibitor
PALRQFQLSAQ	1258.44	0.21	0.35	Glycinin	FQ, LR, PA, AL, QF, QL	ACE inhibitor, DPP-IV inhibitor
SHQKIRHFNEGDVL	1679.86	0.30	0.11	Glycinin	IR, GD, EG, QK, FNE, VL, RHF, FN, HF, KI, NE, RH, SH	ACE inhibitor, antioxidant, glucose uptake stimulation, DPP-IV inhibitor
YKNGIYSPHWN	1378.51	0.42	0.07	Glycinin	IY, GI, NG, YK, PH, IYSPH, PHW, SP, WN, HW, YS	ACE inhibitor, antioxidant, DPP-IV inhibitor
FYNPKAGRIST	1253.42	0.29	0.13	Glycinin	FY, AG, GR, KA, FYN, ST, YN, NP, PK, RI	ACE inhibitor, DPP-IV inhibitor
GPLVNPESQQGSPR	1465.59	0.30	0.14	Glycinin	GP, PR, GPL, PL, GS, QG, VNP, LV, VNPESQQGSPR, NP, ES, QG, QQ, VN, PE	ACE inhibitor, antioxidant, antiamnestic, antithrombotic, glucose uptake stimulation, regulating the stomach mucosal membrane activity, α-glucosidase inhibitor, DPP-IV inhibitor
YKNGIYSPHW	1264.41	0.48	0.07	Glycinin	IY, GI, NG, YK, PH, IYSPH, PHW, SP, HW, YS	ACE inhibitor, antioxidant, DPP-IV inhibitor
NALEPDHRVESEGGL	1622.71	0.27	0.11	Glycinin	GL, GG, EG, VE, ALEP, SE, ALEPDHR, NALEPDHRVESEGG, EP, AL, ES, HR, NA	ACE inhibitor, antioxidant, stimulating vasoactive substance release, α-glucosidase inhibitor, DPP-IV inhibitor

YKNGIYSPHWNLNA	1676.85	0.52	0.10	Glycinin	IY, GI, NG, YK, LN, PH, IYSPH, PHW, SP, HW, YS	ACE inhibitor, antioxidant, DPP-IV inhibitor
SGFSKHFLAQSFNTNED	1929.03	0.35	0.07	Glycinin	LA, GF, SG, SF, FL, FN, HF, KH, NE, NT, QS, SK, TN	ACE inhibitor, DPP-IV inhibitor
VIPPGVPYWTYNTGDE	1807.98	0.31	0.09	Glycinin	IPP, PG, YW, IP, VP, GV, GD, TG, PP, YN, TY, PY, PPG, WT, NT, VI	ACE inhibitor, antioxidant, antiinflammation, *α*-amylase inhibitor, antiamnestic, antithrombotic, regulating the stomach mucosal membrane activity, *α*-glucosidase inhibitor, DPP-IV inhibitor
HENIARPSRADF	1412.53	0.30	0.13	Glycinin	IA, RP, RA, AR, DF, ADF, AD, HE, PS	ACE inhibitor, antioxidant, *α*-glucosidase inhibitor, DPP-IV inhibitor
FSKHFLAQSF	1211.39	0.57	0.08	Glycinin	LA, SF, FL, HF, KH, QS, SK	ACE inhibitor, DPP-IV inhibitor
FSKHFLAQSFNTNE	1669.81	0.30	0.08	Glycinin	LA, SF, FL, FN, HF, KH, NE, NT, QS, TN	ACE inhibitor, DPP-IV inhibitor
SGFSKHFLAQSF	1355.52	0.72	0.07	Glycinin	LA, GF, SG, SF, FL, HF, KH, QS	ACE inhibitor, DPP-IV inhibitor
NSLTLPALRQ	1112.29	0.24	0.49	Glycinin	LR, LP, SL, PA, AL, LT, TL	ACE inhibitor, DPP-IV inhibitor
LAGNPDIEYPET	1318.40	0.30	0.07	Glycinin	YP, LA, AG, EY, IE, IEY, NP, ET, PE	ACE inhibitor, *α*-glucosidase inhibitor, DPP-IV inhibitor
KLQSPDDERKQIV	1555.75	0.20	0.20	Glycinin	KL, LQ, ER, IV, SP, QI, QS, RK	ACE inhibitor, glucose uptake stimulation, DPP-IV inhibitor
IIIAQGKGALG	1040.27	0.37	0.13	Glycinin	IA, GA, KG, GK, QG, LG, IAQ, II, AL	ACE inhibitor, glucose uptake stimulation, DPP-IV inhibitor
YNPKAGRISTL	1219.41	0.31	0.20	Glycinin	AG, GR, KA, ST, YN, NP, PK, RI, TL	ACE inhibitor, DPP-IV inhibitor
DQTPRVFY	1025.13	0.45	0.15	Glycinin	VF, PR, FY, TP, DQ, QT	ACE inhibitor, DPP-IV inhibitor
VIPPGVPYWTYNTGDE PV	2004.23	0.20	0.10	Glycinin	IPP, PG, YW, IP, VP, GV, GD, TG, PP, YN, TY, PY, PPG, WT, NT, VI	ACE inhibitor, antioxidant, antiinflammation, antiamnestic, *α*-amylase inhibitor, antithrombotic, regulating the stomach mucosal membrane activity, *α*-glucosidase inhibitor, DPP-IV inhibitor
DEDEQIPSHPPRRPSHG	1954.05	0.38	0.09	Glycinin	PR, IP, HG, SHP, RR, PP, DEQIPSHPPR, PS, QI, SH	ACE inhibitor, antioxidant, *α*-glucosidase inhibitor, DPP-IV inhibitor
SGFSKHFLAQS	1208.34	0.46	0.08	Glycinin	LA, GF, SG, FL, HF, KH, QS, SK	ACE inhibitor, DPP-IV inhibitor
FSKHFLAQS	1064.21	0.34	0.09	Glycinin	LA, FL, HF, KH, QS, SK	ACE inhibitor, DPP-IV inhibitor
VFDGELRRGQ	1176.30	0.28	0.25	Glycinin	VF, GQ, GE, DG, RR, RG, LR, EL	ACE inhibitor, antioxidant, neuropeptide, DPP-IV inhibitor
FYLAGNPDIEY	1301.42	0.29	0.06	Glycinin	YL, FY, AG, EY, LA, IE, IEY, NP	ACE inhibitor, neuropeptide, DPP-IV inhibitor

NSLTLPALRQFQLSAQY	1950.22	0.30	0.34	Glycinin	FQ, LR, LP, PA, AL, SL, LT, QF, QL, QY, TL	ACE inhibitor, DPP-IV inhibitor
PALRQFQLS	1059.23	0.33	0.31	Glycinin	FQ, LR, PA, AL, QF, QL	ACE inhibitor, DPP-IV inhibitor
VIPPGVPYWTYNTGD	1678.86	0.47	0.10	Glycinin	IPP, PG, YW, IP, VP, GV, GD, TG, PP, YN, TY, PY, PPG, WT, NT, VI	ACE inhibitor, antioxidant, antiinflammation, antiamnestic, α-amylase inhibitor, antithrombotic, regulating the stomach mucosal membrane activity, α-glucosidase inhibitor, DPP-IV inhibitor
NSLTLPALRQFQLS	1587.84	0.35	0.35	Glycinin	FQ, LR, LP, PA, AL, SL, LT, QF, QL, TL	ACE inhibitor, DPP-IV inhibitor
VIPPGVPYWTYN	1405.62	0.69	0.10	Glycinin	IPP, PG, YW, IP, VP, GV, GD, TG, PP, YN, TY, PY, PPG, WT, NT, VI	ACE inhibitor, antioxidant, antiinflammation, antiamnestic, α-amylase inhibitor, antithrombotic, regulating the stomach mucosal membrane activity, α-glucosidase inhibitor, DPP-IV inhibitor
DFYNPKAG	910.98	0.63	0.08	Glycinin	FY, AG, KA, FYN, DF, YN, DFY, NP, PK	ACE inhibitor, DPP-IV inhibitor
NNQLDQTPRV	1184.27	0.21	0.31	Glycinin	PR, TP, DQ, NN, NQ, QL, QT	ACE inhibitor, DPP-IV inhibitor
LVIPPGVPYWTYNTGDE	1921.14	0.27	0.10	Glycinin	IPP, PG, YW, IP, VP, GV, GD, TG, PP, YN, TY, PY, PPG, WT, NT, VI	ACE inhibitor, antioxidant, antiinflammation, antiamnestic, α-amylase inhibitor, antithrombotic, regulating the stomach mucosal membrane activity, α-glucosidase inhibitor, DPP-IV inhibitor
IIIAQGKGAL	983.22	0.32	0.13	Glycinin	IA, GA, KG, GK, QG, IAQ, II, AL	ACE inhibitor, glucose uptake stimulation, DPP-IV inhibitor
NIARPSRADF	1146.27	0.37	0.16	Glycinin	IA, RP, RA, AR, DF, ADF, AD, PS	ACE inhibitor, antioxidant, α-glucosidase inhibitor, DPP-IV inhibitor
GLHLPSYSPYPRM	1517.77	0.79	0.11	Glycinin	LHLP, PYP, YPR, YP, PSY, GL, HL, NG, SY, LP, LH, LHL, PY, NR, PS, RN	ACE inhibitor, antioxidant, antiinflammation, α-glucosidase inhibitor, DPP-IV inhibitor
NNQLDQTPRVFY	1494.63	0.24	0.20	Glycinin	VF, PR, FY, TP, DQ, NN, NQ, QL, QT	ACE inhibitor, DPP-IV inhibitor
YLAGNPDIEYPET	1481.58	0.23	0.07	Glycinin	YL, YP, LA, AG, EY, IE, IEY, NP, ET, PE	ACE inhibitor, neuropeptide, α-glucosidase inhibitor, DPP-IV inhibitor
LEPDHRVESEGGL	1437.53	0.23	0.10	Glycinin	GL, GG, EG, VE, SE, EP, EG, ES, HR	ACE inhibitor, stimulating vasoactive substance release, α-glucosidase inhibitor, DPP-IV inhibitor
VFDGELRRGQL	1289.46	0.36	0.31	Glycinin	VF, GQ, GE, DG, RR, RG, LR, EL, QL	ACE inhibitor, antioxidant, neuropeptide, DPP-IV inhibitor
NNQLDQTPRVF	1331.45	0.32	0.26	Glycinin	VF, PR, TP, DQ, NN, NQ, QL, QT	ACE inhibitor, DPP-IV inhibitor
LLVVPQNFVVA	1198.47	0.22	0.30	Glycinin	VP, NF, PQ, LV, LL, VA, VV, NF, QN	ACE inhibitor, glucose uptake stimulation, DPP-IV inhibitor

YKNGIYSPHWNL	1491.67	0.65	0.09	Glycinin	IY, GI, NG, YK, PH, IYSPH, PHW, SP, WN, HW, YS	ACE inhibitor, antioxidant, DPP-IV inhibitor
GLHLPSYSPYPR	1386.57	0.67	0.11	Glycinin	LHLP, PYP, YPR, YP, PSY, GL, HL, NG, SY, LP, LH, LHL, PY, PS	ACE inhibitor, antioxidant, antiinflammation, α-glucosidase inhibitor, DPP-IV inhibitor
LVVPQNF	815.97	0.30	0.16	Glycinin	VP, NF, PQ, LV, VV, QN	ACE inhibitor, glucose uptake stimulation, DPP-IV inhibitor
LVIPPGVPY	954.18	0.34	0.11	Glycinin	IPP, PG, IP, VP, GV, PP, PY, PPG, VI	ACE inhibitor, antiinflammation, antiamnestic, α-amylase inhibitor, antithrombotic, regulating the stomach mucosal membrane activity, α-glucosidase inhibitor, DPP-IV inhibitor
YNPKAGRISTLNSLT	1634.85	0.37	0.20	Glycinin	AG, GR, KA, LN, ST, YN, SL, NP, LT, PK, RI, TL	ACE inhibitor, DPP-IV inhibitor
SKHFLAQSF	1064.21	0.51	0.09	Glycinin	LA, SF, FL, HF, KH, QS, SK	ACE inhibitor, DPP-IV inhibitor
FNEGDVLVIPPGVPY	1615.85	0.36	0.08	Glycinin	IPP, PG, IP, VP, GV, GD, EG, PP, FNE, PPG, VI	ACE inhibitor, antioxidant, antiinflammation, antiamnestic, α-amylase inhibitor, antithrombotic, regulating the stomach mucosal membrane activity, α-glucosidase inhibitor, DPP-IV inhibitor
NIARPSRADFYNPKAG	1776.97	0.28	0.19	Glycinin	FY, IA, RP, RA, AG, AR, KA, FYN, DF, YN, DFY, RADFY, ADF, NP, AD, PK, PS	ACE inhibitor, antioxidant, α-glucosidase inhibitor, DPP-IV inhibitor
LVVPQNFVVA	1085.31	0.20	0.25	Glycinin	VP, NF, PQ, LV, VA, VV, QN	ACE inhibitor, glucose uptake stimulation, DPP-IV inhibitor
WNSQHPEL	1010.07	0.50	0.10	Glycinin	HP, EL, PEL, WN, QH, PE	ACE inhibitor, antioxidant, α-glucosidase inhibitor, DPP-IV inhibitor
SVISPKWQEQ	1201.34	0.20	0.10	Glycinin	KW, SP, WQ, PK, QE, SV, VI	ACE inhibitor, DPP-IV inhibitor
SGFSKHFLAQSFNTNEDIAE	2242.39	0.27	0.07	Glycinin	IA, LA, GF, SG, SF, IAE, FL, AE, FN, HF, KH, NE, NT, QS, SK, TN	ACE inhibitor, DPP-IV inhibitor
DFYNPKAGRIST	1368.51	0.36	0.11	Glycinin	FY, AG, GR, KA, FYN, DF, ST, YN, DFY, NP, PK, RI	ACE inhibitor, DPP-IV inhibitor
PALRQFQLSA	1130.31	0.30	0.34	Glycinin	FQ, LR, PA, AL, QF, QL	ACE inhibitor, DPP-IV inhibitor
FRAIPSEV	918.06	0.23	0.10	Glycinin	AIP, IP, RA, FR, AI, EV, SE, PS	ACE inhibitor, stimulating vasoactive substance release, DPP-IV inhibitor
DQTPRVF	861.95	0.51	0.20	Glycinin	VF, PR, TP, DQ, QT	ACE inhibitor, DPP-IV inhibitor

YLKDVF	783.92	0.41	0.13	Glycinin	VF, YL, KD, LK	ACE inhibitor, antioxidant, neuropeptide, DPP-IV inhibitor
ALEPDHRVESEGGL	1508.61	0.31	0.12	Glycinin	GL, GG, EG, VE, ALEP, SE, ALEPDHR, EP, AL, ES, HR	ACE inhibitor, antioxidant, stimulating vasoactive substance release, α-glucosidase inhibitor, DPP-IV inhibitor
RQFQLSA	848.96	0.25	0.26	Glycinin	FQ, QF, QL	ACE inhibitor, DPP-IV inhibitor
GIYSPHWN	973.06	0.73	0.06	Glycinin	IY, GI, PH, IYSPH, PHW, SP, WN, HW, YS	ACE inhibitor, antioxidant, DPP-IV inhibitor
ISLLDTSNF	1009.12	0.25	0.07	Glycinin	NF, LL, SL, TS	ACE inhibitor, glucose uptake stimulation, DPP-IV inhibitor
EGGSVLSGFSKHFLAQS	1750.93	0.53	0.08	Glycinin	LA, GF, GS, GG, SG, EG, VL, SVL, FL, HF, KH, QS, SK, SV	ACE inhibitor, antioxidant, glucose uptake stimulation, DPP-IV inhibitor
LKYEGNWGPL	1176.34	0.64	0.20	Glycinin	GP, GPL, PL, WG, EG, KY, YE, LK, NW	ACE inhibitor, antioxidant, antiamnestic, antithrombotic, regulating the stomach mucosal membrane activity, DPP-IV inhibitor
VLVIPPGVPYWTYNTGDE	2020.27	0.27	0.12	Glycinin	IPP, PG, YW, IP, VP, GV, GD, TG, PP, YN, TY, PY, PPG, WT, NT, VI	ACE inhibitor, antioxidant, antiinflammation, antiamnestic, α-amylase inhibitor, antithrombotic, glucose uptake stimulation, regulating the stomach mucosal membrane activity, α-glucosidase inhibitor, DPP-IV inhibitor
LDTSNFNNQL	1165.22	0.26	0.10	Glycinin	NF, FN, NN, NQ, QL, TS	ACE inhibitor, DPP-IV inhibitor
EDEDDEDEQIPSHPPRR	2064.07	0.29	0.08	Glycinin	PR, IP, SHP, RR, HP, DEQIPSHPPR, PP, PS, QI, SH	ACE inhibitor, antioxidant, α-glucosidase inhibitor, DPP-IV inhibitor
EGGLSVISPKWQEQQ	1685.85	0.20	0.11	Glycinin	KW, GL, GG, EG, SP, WQ, PK, QE, QQ, SV, VI	ACE inhibitor, DPP-IV inhibitor
VIPPGVPY	841.02	0.42	0.08	Glycinin	IPP, PG, IP, VP, GV, PP, PY, PPG, VI	ACE inhibitor, antiinflammation, antiamnestic, α-amylase inhibitor, antithrombotic, regulating the stomach mucosal membrane activity, α-glucosidase inhibitor, DPP-IV inhibitor
VIPPGVP	677.84	0.46	0.12	Glycinin	IPP, PG, IP, VP, GV, PP, PPG, VI	ACE inhibitor, antiinflammation, antiamnestic, α-amylase inhibitor, antithrombotic, regulating the stomach mucosal membrane activity, α-glucosidase inhibitor, DPP-IV inhibitor
FAFGINAENNQRNFLAGSKD	2213.39	0.23	0.12	Conglycinin	AF, LA, GI, AG, FG, GS, NF, AGS, KD, FA, FL, AE, IN, NA, NN, NQ, RN, SK	ACE inhibitor, antioxidant, DPP-IV inhibitor

RSRDPIYSNKLGKL	1646.91	0.61	0.27	Conglycin in	IY, GK, LG, NKL, KL, NK, DP, PI, YS	ACE inhibitor, antioxidant, DPP-IV inhibitor
LQRFNKRSQQLQNLRD	2044.30	0.28	0.57	Conglycin in	RF, KR, NK, LQ, LR, NLR, FN, NL, QL, QN, QQ	ACE inhibitor, DPP-IV inhibitor
YVVNPDNDENLRM	1578.72	0.29	0.13	Conglycin in	VNP, YV, LR, NLR, LRM, VV, NP, DN, ND, NL, RM, VN	ACE inhibitor, DPP-IV inhibitor
FEITPEKNPQLRDLDVF L	2174.48	0.39	0.15	Conglycin in	VF, EI, PQ, EK, TP, LR, VFL, KNPQLR, EITPEKNPQLR, NP, FL, QL, PE	ACE inhibitor, antioxidant, fatty acid synthase inhibitor, *α*-glucosidase inhibitor, DPP-IV inhibitor
SYNLQSGDALRVPAGT T	1749.90	0.21	0.25	Conglycin in	VP, AG, DA, GT, SG, GD, SY, LQ, YN, LR, YNL, NLQ, PA, AL, NL, QS, TT	ACE inhibitor, antioxidant, antiinflammation, DPP-IV inhibitor
PIYSNKLGKL	1132.37	0.67	0.18	Conglycin in	IY, GK, LG, NKL, KL, NK, PI, YS	ACE inhibitor, antioxidant, DPP-IV inhibitor
QSGDALRVPAGTT	1272.38	0.37	0.30	Conglycin in	VP, AG, DA, GT, SG, GD, LR, PA, AL, QS, TT	ACE inhibitor, DPP-IV inhibitor
VDMNEGALFLPHFNSK A	1890.14	0.45	0.11	Conglycin in	LF, GA, EG, KA, PH, DM, LP, PHF, AL, FN, HF, MN, NE, SK, VD	ACE inhibitor, antioxidant, DPP-IV inhibitor
LAFPGSAKDIENLIKSQ	1831.10	0.26	0.11	Conglycin in	PG, FP, AFP, AF, LA, GS, IE, LI, KD, LAF, KS, NL	ACE inhibitor, antioxidant, antiamnestic, antithrombotic, glucose uptake stimulation, regulating the stomach mucosal membrane activity, DPP-IV inhibitor
FKNQYGHVRVLQ	1488.71	0.22	0.27	Conglycin in	YG, GH, YR, LQ, VL, VR, HV, NQ, QY	ACE inhibitor, antiinflammation, glucose uptake stimulation, DPP-IV inhibitor
SVVDMNEGALFLPHFN S	1877.10	0.44	0.08	Conglycin in	LF, GA, EG, KA, PH, DM, LP, PHF, VV, AL, FN, HF, MN, NE, SV, VD	ACE inhibitor, antioxidant, DPP-IV inhibitor
FLSSTQAQQSYLQG	1557.68	0.21	0.11	Conglycin in	YL, QG, SY, LQ, TQ, ST, FL, QA, QQ, QS, TQ	ACE inhibitor, neuropeptide, DPP-IV inhibitor

GALFLPHFNS	1102.26	0.67	0.08	Conglycin in	LF, GA, PH, LP, PHF, FL, AL, FN, HF	ACE inhibitor, antioxidant, DPP-IV inhibitor
GALFLPHFNSKA	1301.51	0.77	0.14	Conglycin in	LF, GA, KA, PH, LP, PHF, FL, AL, FN, HF	ACE inhibitor, antioxidant, DPP-IV inhibitor
FLPHFNSKA	1060.22	0.63	0.10	Conglycin in	KA, PH, LP, PHF, FL, FN, HF	ACE inhibitor, antioxidant, DPP-IV inhibitor
LFLPHFNSKA	1173.38	0.55	0.13	Conglycin in	LF, KA, PH, LP, PHF, FL, FN, SK, HF	ACE inhibitor, antioxidant, DPP-IV inhibitor
LPHHADADYL	1151.24	0.33	0.10	Conglycin in	YL, DA, PH, DY, LP, PHH, LPHH, HH, HA, AD	ACE inhibitor, antioxidant, neuropeptide, α-glucosidase inhibitor, DPP-IV inhibitor
FAFGINAENNQ	1224.29	0.21	0.07	Conglycin in	AF, GI, FG, FA, AE, IN, NA, NN, NQ	ACE inhibitor, DPP-IV inhibitor
VVNPDNDENLRM	1415.54	0.36	0.15	Conglycin in	VNP, LR, NLR, LRM, VV, NP, DN, ND, NL, RM, VN	ACE inhibitor, DPP-IV inhibitor
NLQSGDALRVPAGTT	1499.64	0.26	0.32	Conglycin in	VP, AG, DA, GT, SG, GD, LQ, LR, NLQ, PA, AL, NL, QS, TT, RV	ACE inhibitor, antiinflammation, DPP-IV inhibitor
FLSSTQAQQSYLQGFSKN	2034.21	0.23	0.12	Conglycin in	YL, GF, QG, SY, LQ, TQ, ST, FL, QA, QQ, QS, SK	ACE inhibitor, neuropeptide, DPP-IV inhibitor
QREPRRHKNKNPF	1706.93	0.35	0.52	Conglycin in	PR, NK, RR, HK, RHK, EP, NP, PF, RH	ACE inhibitor, antioxidant, DPP-IV inhibitor
PHHADADYLI	1151.24	0.43	0.07	Conglycin in	YL, DA, PH, DY, LI, LP, PHH, HH, HA, AD	ACE inhibitor, antioxidant, neuropeptide, glucose uptake stimulation, α-glucosidase inhibitor, DPP-IV inhibitor
FEITPEKNPQLRDLDVF	2061.32	0.32	0.13	Conglycin in	VF, EI, PQ, EK, TP, LR, KNPQLR, EITPEKNPQLR, NP, QL, VF, PE	ACE inhibitor, fatty acid synthase inhibitor, α-glucosidase inhibitor, DPP-IV inhibitor
FNSKPNTLLLPHHADAD	1890.09	0.23	0.14	Conglycin in	LLP, DA, KP, PH, LP, LLL, LL, PHH, LLPH, LPHH, HH, LLPHH, HA, AD, FN, NT, PN, SK, TL	ACE inhibitor, antioxidant, stimulating vasoactive substance release, glucose uptake stimulation, α-glucosidase inhibitor, DPP-IV inhibitor

LAFPGSAKDIEN	1261.40	0.26	0.09	Conglycin in	PG, FP, AFP, AF, LA, GS, IE, KD, LAF	ACE inhibitor, antioxidant, antiamnestic, antithrombotic, regulating the stomach mucosal membrane activity, DPP-IV inhibitor
LAFPGSAKD	905.02	0.27	0.13	Conglycin in	PG, FP, AFP, AF, LA, GS, KD, LAF	ACE inhibitor, antioxidant, antiamnestic, antithrombotic, regulating the stomach mucosal membrane activity, DPP-IV inhibitor
RDLDVF	763.85	0.47	0.17	Conglycin in	VF	ACE inhibitor, DPP-IV inhibitor
SGDALRVPAGTT	1144.25	0.40	0.26	Conglycin in	VP, AG, DA, GT, SG, GD, LQ, LR, PA, AL, TT, RV	ACE inhibitor, DPP-IV inhibitor
LAFPGSAKDIENL	1374.56	0.42	0.11	Conglycin in	PG, FP, AFP, AF, LA, GS, IE, KD, LAF	ACE inhibitor, antioxidant, antiamnestic, antithrombotic, regulating the stomach mucosal membrane activity, DPP-IV inhibitor
LQSGDALRVPAGTT	1385.54	0.30	0.33	Conglycin in	VP, AG, DA, GT, SG, GD, LQ, LR, PA, AL, TT, RV	ACE inhibitor, DPP-IV inhibitor
KNPQLRDLDVF	1344.53	0.46	0.28	Conglycin in	VF, PQ, LR, KNPQLR, NP, QL	ACE inhibitor, fatty acid synthase inhibitor, DPP-IV inhibitor
GALFLPH	753.90	0.78	0.14	Conglycin in	LF, GA, PH, LP, FL, AL	ACE inhibitor, DPP-IV inhibitor
LAFPGSAK	789.93	0.36	0.17	Conglycin in	PG, FP, AFP, AF, LA, GS, LAF	ACE inhibitor, antioxidant, antiamnestic, antithrombotic, regulating the stomach mucosal membrane activity, DPP-IV inhibitor
NSKPNTLLLPH	1233.43	0.33	0.25	Conglycin in	LLP, KP, PH, LP, LLL, LL, LLPH, NT, PN, SK, TL	ACE inhibitor, antioxidant, stimulating vasoactive substance release, glucose uptake stimulation, DPP-IV inhibitor
LFLPH	625.77	0.72	0.14	Conglycin in	LF, PH, LP, FL	ACE inhibitor, DPP-IV inhibitor
LFLPHF	772.95	0.93	0.10	Conglycin in	LF, PH, LP, PHF, FL, HF	ACE inhibitor, antioxidant, DPP-IV inhibitor
FSKNIL	720.87	0.56	0.10	Conglycin in	IL, SK	ACE inhibitor, glucose uptake stimulation, DPP-IV inhibitor

AFGINAENNQRN	1347.41	0.20	0.12	Conglycinin	AF, GI, FG, AE, IN, NA, NN, NQ, RN	ACE inhibitor, DPP-IV inhibitor
FLAGSKDNVISQIPS	1575.78	0.21	0.08	Conglycinin	IP, LA, AG, GS, AGS, KD, FL, DN, NV, PS, QI, SK, VI	ACE inhibitor, antioxidant, DPP-IV inhibitor
YVVNPDNDENLRMIT	1792.98	0.22	0.12	Conglycinin	VNP, YV, LR, NLR, LRM, VV, NP, DN, NL, RM, VN	ACE inhibitor, DPP-IV inhibitor
LAFPGSAKDIENLIKS	1702.97	0.32	0.11	Conglycinin	PG, FP, AFP, AF, LA, GS, IE, LI, KD, LAF, KS, NL	ACE inhibitor, antioxidant, antiamnestic, antithrombotic, glucose uptake stimulation, regulating the stomach mucosal membrane activity, DPP-IV inhibitor
SKPNTLLLPHHADAD	1628.81	0.20	0.17	Conglycinin	LLP, DA, KP, PH, LP, LLL, LL, PHH, LLPH, LPHH, HH, LLPHH, HA, AD, FN, NT, PN, SK, TL	ACE inhibitor, antioxidant, stimulating vasoactive substance release, glucose uptake stimulation, α-glucosidase inhibitor, DPP-IV inhibitor
LFLPHFNS	974.13	0.54	0.08	Conglycinin	LF, PH, LP, PHF, FL, FN. HF	ACE inhibitor, antioxidant, DPP-IV inhibitor
KIIKLAIPVNKPGRYDD	1940.32	0.20	0.18	Conglycinin	PG, RY, AIP, IP, LA, GR, AI, KL, NK, KP, II, IKL, KI, PV, VN, YD	ACE inhibitor, antioxidant, antiamnestic, antithrombotic, glucose uptake stimulation, regulating the stomach mucosal membrane activity, DPP-IV inhibitor
LAFPGSAQDVERLLK	1643.90	0.34	0.33	Conglycinin	PG, RL, FP, AFP, AF, LA, GS, VE, ER, LL, LAK, QD, VE	ACE inhibitor, antioxidant, antiamnestic, antithrombotic, glucose uptake stimulation, regulating the stomach mucosal membrane activity, α-glucosidase inhibitor, DPP-IV inhibitor
LAFPGSAQDVERLLKK	1772.08	0.29	0.41	Conglycinin	PG, RL, FP, AFP, AF, LA, GS, VE, ER, LL, LAK, QD, VE	ACE inhibitor, antioxidant, antiamnestic, antithrombotic, glucose uptake stimulation, regulating the stomach mucosal membrane activity, α-glucosidase inhibitor, DPP-IV inhibitor
VDINEGALLLPHFNSKA	1838.09	0.45	0.13	Conglycinin	LLP, GA, EG, KA, PH, LP, LLL, LL, LLPH, PHF, AL, EG, FN, HF, IN, NE, SK, VD	ACE inhibitor, antioxidant, stimulating vasoactive substance release, glucose uptake stimulation, DPP-IV inhibitor
LAFGINAENNQRN	1460.57	0.20	0.14	Conglycinin	AF, LA, GI, FG, LAF, AE, IN, NA, NN, NQ, RN	ACE inhibitor, antioxidant, DPP-IV inhibitor

LQRFNKRSPQLENLRD	2014.27	0.33	0.47	Conglycinin	RF, KR, NK, LQ, PQ, LR, NLR, SP, FN, NL, QL	ACE inhibitor, DPP-IV inhibitor
FQSKPNTILLPHHADAD	1904.11	0.20	0.11	Conglycinin	LLP, DA, KP, PH, FQ, IL, LP, LL, PHH, LLPH, LPHH, HH, LLPHH, HA, AD, FN, NT, PN, SK, QS, TI	ACE inhibitor, antioxidant, glucose uptake stimulation, *α*-glucosidase inhibitor, DPP-IV inhibitor
INEGALLLPHFNS	1424.62	0.36	0.09	Conglycinin	LLP, GA, EG, PH, LP, LLL, LL, LLPH, PHF, AL, EG, FN, HF, IN, NE	ACE inhibitor, antioxidant, stimulating vasoactive substance release, glucose uptake stimulation, DPP-IV inhibitor
INEGALLLPHFNSKA	1623.87	0.52	0.13	Conglycinin	LLP, GA, EG, PH, LP, LLL, LL, LLPH, PHF, AL, EG, FN, HF, IN, NE, SK	ACE inhibitor, antioxidant, stimulating vasoactive substance release, glucose uptake stimulation, DPP-IV inhibitor
IKLAIPVNKPG	1149.44	0.32	0.23	Conglycinin	PG, AIP, IP, LA, AI, KL, NK, KP, IKL, PV, VN	ACE inhibitor, antioxidant, antiamnestic, antithrombotic, regulating the stomach mucosal membrane activity, DPP-IV inhibitor
SRNPIYSNNFG	1268.35	0.56	0.07	Conglycinin	IY, FG, NF, NP, NN, PI, RN, YS	ACE inhibitor, antioxidant, DPP-IV inhibitor
LAFGINAENNQRNFL	1720.91	0.40	0.14	Conglycinin	AF, LA, GI, FG, NF, LAF, AE, IN, NA, NN, NQ, RN	ACE inhibitor, antioxidant, DPP-IV inhibitor
LVNPHDHQNLKIIKL	1782.12	0.36	0.19	Conglycinin	KL, PH, VNP, LV, II, VNPHDHQN, LVNPHDHQN, HDH, PHD, IKL, LK, NP, HD, KI, NL, QN, VN	ACE inhibitor, antioxidant, glucose uptake stimulation, DPP-IV inhibitor
DRDSYNLHPGDAQRIPAGTT	2184.31	0.20	0.16	Conglycinin	PG, IPA, IP, AG, DA, GT, GD, HP, LHPGDAQR, YN, YNL, DR, LH, PA, NL, RI, SY, TT	ACE inhibitor, antioxidant, antiamnestic, antithrombotic, regulating the stomach mucosal membrane activity, DPP-IV inhibitor
INEGALLLPH	1076.26	0.33	0.14	Conglycinin	LLP, GA, EG, PH, LP, LLL, LL, LLPH, AL, EG, IN, NE	ACE inhibitor, antioxidant, stimulating vasoactive substance release, glucose uptake stimulation, DPP-IV inhibitor
LAFPGSAQDVERLLKKQRE	2185.51	0.21	0.45	Conglycinin	PG, RL, FP, AFP, AF, LA, GS, VE, ER, LL, LAK, QD, VE	ACE inhibitor, antioxidant, antiamnestic, antithrombotic, glucose uptake stimulation, regulating the stomach mucosal membrane activity, *α*-glucosidase inhibitor, DPP-IV inhibitor

LSSTQAQQSYLQGFSHNILE	2251.44	0.26	0.09	Conglycinin	YL, GF, QG, SY, LQ, TQ, IL, ST, QA, QQ, QS, SH	ACE inhibitor, neuropeptide, glucose uptake stimulation, DPP-IV inhibitor
IKLAIPVNKPGRYDDF	1846.16	0.31	0.14	Conglycinin	PG, RY, AIP, IP, LA, GR, AI, KL, NK, KP, DF, IKL, PV, VN	ACE inhibitor, antioxidant, antiamnestic, antithrombotic, regulating the stomach mucosal membrane activity, DPP-IV inhibitor
LAFPGSAQDVERL	1402.57	0.43	0.19	Conglycinin	PG, RL, FP, AFP, AF, LA, GS, VE, ER, LL, LAK, QD, VE	ACE inhibitor, antioxidant, antiamnestic, antithrombotic, regulating the stomach mucosal membrane activity, α-glucosidase inhibitor, DPP-IV inhibitor
AIPVNKPGRYDD	1344.49	0.27	0.13	Conglycinin	PG, RY, AIP, IP, GR, AI, NK, KP, PY, PV, VN	ACE inhibitor, antioxidant, antiamnestic, antithrombotic, regulating the stomach mucosal membrane activity, DPP-IV inhibitor
LSSVDINEGALLLPHFNSKA	2125.41	0.61	0.12	Conglycinin	LLP, GA, EG, KA, PH, LP, LLL, LL, LLPH, PHF, AL, EG, IN, NE, SV, VD	ACE inhibitor, antioxidant, stimulating vasoactive substance release, glucose uptake stimulation, DPP-IV inhibitor
QSKPNTILLPH	1247.46	0.38	0.16	Conglycinin	LLP, KP, PH, IL, LP, LL, LLPH, NT, PN, QS, SK	ACE inhibitor, antioxidant, glucose uptake stimulation, DPP-IV inhibitor
QGFSHNILETSF	1379.49	0.49	0.06	Conglycinin	QF, QG, SF, IL, ET, GF, SH, TS	ACE inhibitor, glucose uptake stimulation, DPP-IV inhibitor
PIYSNNFG	910.98	0.70	0.05	Conglycinin	IY, FG, NF, NN, PI, YS	ACE inhibitor, antioxidant, DPP-IV inhibitor
LAIPVNKPGRYD	1342.56	0.27	0.19	Conglycinin	PG, RY, AIP, IP, LA, GR, AI, NK, KP, PY, PV, VN, YD	ACE inhibitor, antioxidant, antiamnestic, antithrombotic, regulating the stomach mucosal membrane activity, DPP-IV inhibitor
ILLPHHADADFLL	1474.72	0.50	0.12	Conglycinin	LLP, DA, PH, DF, IL, LP, LL, PHH, LLPH, LPHH, HH, LLPHH, HA, AD	ACE inhibitor, antioxidant, glucose uptake stimulation, α-glucosidase inhibitor, DPP-IV inhibitor
YLVNPHDHQNLKI	1590.80	0.27	0.12	Conglycinin	YL, PH, VNP, LV, VNPHDHQN, LVNPHDHQN, HDH, PHD, LK, NP, HD, KI, NL, PH, QN, VN	ACE inhibitor, antioxidant, neuropeptide, glucose uptake stimulation, DPP-IV inhibitor
LFVLSGRAIL	1088.36	0.40	0.27	Conglycinin	LF, RA, GR, AI, SG, IL, VL, LFV	ACE inhibitor, antioxidant, glucose uptake stimulation, DPP-IV inhibitor

LAFGINAENNQRNF	1607.75	0.35	0.12	Conglycinin	AF, LA, GI, FG, NF, LAF, AE, IN, NA, NN, NQ, RN	ACE inhibitor, antioxidant, DPP-IV inhibitor
YLVNPHDHQNL	1349.47	0.29	0.10	Conglycinin	YL, PH, VNP, LV, VNPHDHQN, LVNPHDHQN, HDH, PHD, NP, HD, NL, QN, VN	ACE inhibitor, antioxidant, neuropeptide, glucose uptake stimulation, DPP-IV inhibitor
LQRFNKRSPQLENL	1743.00	0.37	0.43	Conglycinin	RF, KR, NK, LQ, PQ, SP, FN, NL, QL	ACE inhibitor, DPP-IV inhibitor
NLHPGDAQRIPAGTT	1547.69	0.25	0.20	Conglycinin	PG, IPA, IP, AG, DA, GT, GD, HP, LHPGDAQR, LH, PA, NL, RI, TT	ACE inhibitor, antioxidant, antiamnestic, antithrombotic, regulating the stomach mucosal membrane activity, DPP-IV inhibitor
LAFGINAENNQRNFLAG	1849.04	0.33	0.15	Conglycinin	AF, LA, GI, AG, FG, NF, LAF, AE, IN, NA, NN, NQ, RN	ACE inhibitor, antioxidant, DPP-IV inhibitor
LRSSNSFQTL	1152.27	0.32	0.15	Conglycinin	SF, FQ, LR, QT, TL	ACE inhibitor, DPP-IV inhibitor
SSVDINEGALLLPHFNS	1813.00	0.57	0.08	Conglycinin	LLP, GA, EG, PH, LP, LLL, LL, LLPH, PHF, AL, EG, IN, NE, SV, VD	ACE inhibitor, antioxidant, stimulating vasoactive substance release, glucose uptake stimulation, DPP-IV inhibitor
LQRFNKRSPQLEN	1629.84	0.25	0.37	Conglycinin	RF, KR, NK, LQ, PQ, SP, FN, QL	ACE inhibitor, DPP-IV inhibitor
LQRFNKRSPQLENLR	1899.19	0.44	0.57	Conglycinin	RF, KR, NK, LQ, PQ, LR, NLR, SP, FN, QL	ACE inhibitor, DPP-IV inhibitor
FVLSGRAIL	975.20	0.37	0.21	Conglycinin	RA, GI, AI, SG, IL, VL	ACE inhibitor, glucose uptake stimulation, DPP-IV inhibitor
LAFPGSAQDV	1004.11	0.23	0.10	Conglycinin	PG, RL, FP, AFP, AF, LA, GS, LAK, QD	ACE inhibitor, antioxidant, antiamnestic, antithrombotic, regulating the stomach mucosal membrane activity, DPP-IV inhibitor
FVLSGRA	748.88	0.37	0.26	Conglycinin	RA, GR, SG, VL	ACE inhibitor, glucose uptake stimulation, DPP-IV inhibitor
SFHSEFEEIN	1238.28	0.22	0.04	Conglycinin	SF, EI, EF, EE, SE, HS, IN	ACE inhibitor, hypolipidemic, stimulating vasoactive substance release, DPP-IV inhibitor

LAFPGSAQDVERLL	1515.73	0.41	0.23	Conglycinin	PG, RL, FP, AFP, AF, LA, GS, VE, ER, LL, LAK, QD, VE	ACE inhibitor, antioxidant, antiamnestic, antithrombotic, glucose uptake stimulation, regulating the stomach mucosal membrane activity, *α*-glucosidase inhibitor, DPP-IV inhibitor
LAFPGSAQDVE	1133.22	0.20	0.09	Conglycinin	PG, FP, AFP, AF, LA, GS, VE, LAF, QD, VE	ACE inhibitor, antioxidant, antiamnestic, antithrombotic, regulating the stomach mucosal membrane activity, *α*-glucosidase inhibitor, DPP-IV inhibitor
AIPVNKPGRYDDF	1491.67	0.52	0.11	Conglycinin	PG, RY, AIP, IP, GR, AI, NK, KP, PY, PV, VN, YD	ACE inhibitor, antioxidant, antiamnestic, antithrombotic, regulating the stomach mucosal membrane activity, DPP-IV inhibitor
SPQLENLRD	1071.16	0.26	0.18	Conglycinin	PQ, LR, NLR, SP, NL, QL	ACE inhibitor, DPP-IV inhibitor
LFVLSGRA	862.04	0.33	0.35	Conglycinin	LF, RA, GR, SG, VL, LFV	ACE inhibitor, antioxidant, glucose uptake stimulation, DPP-IV inhibitor
SSVDINEGALLLPH	1464.64	0.57	0.09	Conglycinin	LLP, GA, EG, PH, LP, LLL, LL, LLPH, AL, EG, IN, NE, SV, VD	ACE inhibitor, antioxidant, stimulating vasoactive substance release, glucose uptake stimulation, DPP-IV inhibitor
YRIVQF	824.98	0.37	0.12	Conglycinin	IVQ, IV, YR, QF, RI, VQ	ACE inhibitor, neuropeptide, glucose uptake stimulation, DPP-IV inhibitor
LAFPGSAQD	904.97	0.25	0.09	Conglycinin	PG, FP, AFP, AF, LA, GS, VE, LAF, QD	ACE inhibitor, antioxidant, antiamnestic, antithrombotic, regulating the stomach mucosal membrane activity, DPP-IV inhibitor
VLSGRAIL	828.02	0.22	0.31	Conglycinin	RA, GR, AI, SG, IL, VL	ACE inhibitor, glucose uptake stimulation, DPP-IV inhibitor
FENQNGRIRL	1246.39	0.46	0.21	Conglycinin	RL, IR, GR, NG, NQ, QN	ACE inhibitor, antioxidant, DPP-IV inhibitor
EPFNLRSRNPI	1342.52	0.40	0.16	Conglycinin	LR, NLR, EP, NP, FN, NL, PF, PI, RN	ACE inhibitor, DPP-IV inhibitor
VREDENNPF	1119.16	0.22	0.10	Conglycinin	VR, NP, NN, PF	ACE inhibitor, DPP-IV inhibitor

RSRNPIYSNNFG	1424.54	0.37	0.10	Conglycin in	IY, FG, NF, NP, NN, PI, RN, YS	ACE inhibitor, antioxidant, DPP-IV inhibitor
LVNPHDHQNLKI	1427.63	0.26	0.14	Conglycin in	PH, VNP, LV, VNPHDHQN, LVNPHDHQN, HDH, PHD, LK, NP, HD, KI, NL, QN, VN	ACE inhibitor, antioxidant, glucose uptake stimulation, DPP-IV inhibitor
LFENQNGRIR	1246.39	0.36	0.21	Conglycin in	IR, LF, GR, NG, NQ, QN	ACE inhibitor, antioxidant, DPP-IV inhibitor
LSSVDINEGALLLPHFN S	1926.16	0.43	0.09	Conglycin in	LLP, GA, EG, PH, LP, LLL, LL, LLPH, PHF, AL, EG, FN, IN, NE, SV, VD	ACE inhibitor, antioxidant, stimulating vasoactive substance release, glucose uptake stimulation, DPP-IV inhibitor
PFVVNATSNLN	1175.31	0.20	0.14	Conglycin in	LN, VV, AT, NA, NL, PF, TS	ACE inhibitor, DPP-IV inhibitor
SSVDINEGALLLPHFNS	1813.00	0.57	0.08	Conglycin in	LLP, GA, EG, PH, LP, LLL, LL, LLPH, PHF, AL, EG, FN, IN, NE, SV, VD	ACE inhibitor, antioxidant, stimulating vasoactive substance release, glucose uptake stimulation, DPP-IV inhibitor
SQQQLQDSHQKIRHFN EGDV	2394.55	0.22	0.11	Glycinin	IR, GD, QK, LQ, EG, FNE, RHF, EG, FN, HF, KI, NE, QD, QL, QQ, RH, SH	ACE inhibitor, antioxidant, DPP-IV inhibitor
PALRQFGLS	988.15	0.47	0.27	Glycinin	GL, FG, LR, PA, AL, QF	ACE inhibitor, DPP-IV inhibitor
KYQGNSGPLVNP	1273.41	0.31	0.13	Glycinin	GP, GPL, PL, QG, SG, KY, VNP, SGP, LV, NP, VN	ACE inhibitor, antiamnestic, antithrombotic, glucose uptake stimulation, DPP-IV inhibitor
NSLTLPALRQFGLSAQ	1715.97	0.27	0.36	Glycinin	GL, FG, LR, LP, SL, PA, AL, LT, QF, TL	ACE inhibitor, DPP-IV inhibitor
NSLTLPALRQFGLS	1516.76	0.46	0.33	Glycinin	GL, FG, LR, LP, SL, PA, AL, LT, QF, TL	ACE inhibitor, DPP-IV inhibitor
QLKYQGNSGPLVNP	1514.70	0.39	0.17	Glycinin	GP, GPL, PL, QG, SG, KY, VNP, SGP, LV, NP, QL, VN, YQ	ACE inhibitor, antioxidant, antiamnestic, antithrombotic, glucose uptake stimulation, regulating the stomach mucosal membrane activity, DPP-IV inhibitor
IIVVQGKGAIGF	1201.47	0.36	0.09	Glycinin	GF, IG, GA, KG, GK, QG, AI, IV, II, VV, VQ	ACE inhibitor, glucose uptake stimulation, DPP-IV inhibitor
PALRQFGLSAQ	1187.36	0.26	0.31	Glycinin	GL, FG, LR, PA, AL, QF	ACE inhibitor, DPP-IV inhibitor
LAGNPDIEHPET	1292.37	0.26	0.08	Glycinin	LA, AG, IE, HP, NP, EH, ET	ACE inhibitor, *α*-glucosidase inhibitor, DPP-IV inhibitor
LTLPALRQFGLS	1315.58	0.38	0.42	Glycinin	GL, FG, LR, LP, PA, AL, LT, QF, TL	ACE inhibitor, DPP-IV inhibitor

NNQLDQNPRV	1197.27	0.25	0.25	Glycinin	PR, NPR, NP, DQ, NN, NQ, QL, QN	ACE inhibitor, DPP-IV inhibitor
VRQLKYQGNSGPLVNP	1770.02	0.27	0.29	Glycinin	GP, GPL, PL, QG, SG, VR, KY, VNP, SGP, LV, NP, QL, VN, YQ	ACE inhibitor, antioxidant, antiamnestic, antithrombotic, glucose uptake stimulation, DPP-IV inhibitor
LKYQGNSGPLVNP	1386.57	0.31	0.16	Glycinin	GP, GPL, PL, QG, SG, KY, VNP, SGP, LV, NP, VN, YQ	ACE inhibitor, antioxidant, antiamnestic, antithrombotic, glucose uptake stimulation, regulating the stomach mucosal membrane activity, DPP-IV inhibitor
NSLTLPALRQFGLSA	1587.84	0.37	0.35	Glycinin	GL, FG, LR, LP, SL, PA, AL, LT, QF, TL	ACE inhibitor, DPP-IV inhibitor
FYLAGNPDIEHPET	1602.72	0.26	0.07	Glycinin	YL, FY, LA, AG, IE, HP, EH, ET, PE	ACE inhibitor, neuropeptide, α-glucosidase inhibitor, DPP-IV inhibitor
PALRQFGLSA	1059.23	0.37	0.30	Glycinin	GL, FG, LR, PA, AL, QF	ACE inhibitor, DPP-IV inhibitor
TLNRNGSHLPSYLPYPQ	1957.18	0.22	0.14	Glycinin	PYP, YL, YP, PSY, HL, GS, NG, SY, LN, PQ, GSH, LPYP, PYPQ, NG, NR, PS, RN, SH, TL	ACE inhibitor, antioxidant, antiinflammation, neuropeptide, α-glucosidase inhibitor, DPP-IV inhibitor
NRNGSHLPSYLPYPQ	1742.91	0.28	0.11	Glycinin	PYP, YL, YP, PSY, HL, GS, NG, SY, PQ, GSH, LPYP, PYPQ, NG, NR, PS, RN, SH, TL	ACE inhibitor, antioxidant, antiinflammation, neuropeptide, α-glucosidase inhibitor, DPP-IV inhibitor
PALRQFG	787.92	0.70	0.30	Glycinin	FG, LR, PA, AL, QF	ACE inhibitor, DPP-IV inhibitor
PALRQFGLSAQY	1350.54	0.27	0.25	Glycinin	GL, FG, LR, PA, AL, QY, QF	ACE inhibitor, DPP-IV inhibitor
YLAGNPDIEHPET	1455.54	0.20	0.07	Glycinin	YL, LA, AG, IE, HP, NP, EH, ET, PE	ACE inhibitor, neuropeptide, α-glucosidase inhibitor, DPP-IV inhibitor
AGNPDIEHPET	1179.21	0.28	0.06	Glycinin	AG, IE, HP, NP, EH, ET, PE	ACE inhibitor, α-glucosidase inhibitor, DPP-IV inhibitor
LTLPALRQFGLSAQ	1514.79	0.21	0.44	Glycinin	GL, FG, LR, PA, AL, LT, QF, TL	ACE inhibitor, DPP-IV inhibitor
IIVVQGKGAIGFA	1272.55	0.33	0.10	Glycinin	GF, IG, GA, KG, GK, QG, AI, IV, II, FA, VV, VQ	ACE inhibitor, glucose uptake stimulation, DPP-IV inhibitor
IIVVQGKGAIGFAF	1419.73	0.52	0.09	Glycinin	AF, GF, IG, GA, KG, GK, QG, AI, IV, II, FA, VV, VQ	ACE inhibitor, glucose uptake stimulation, DPP-IV inhibitor
PALRQFGL	901.08	0.78	0.38	Glycinin	GL, FG, LR, PA, AL, QF	ACE inhibitor, DPP-IV inhibitor

RQLKYQGNSGPLVNP	1670.89	0.28	0.27	Glycinin	GP, GPL, PL, QG, SG, KY, VNP, SGP, LV, NP, VN, YQ	ACE inhibitor, antioxidant, antiamnestic, antithrombotic, glucose uptake stimulation, regulating the stomach mucosal membrane activity, DPP-IV inhibitor
DVLVIPLGV	924.15	0.26	0.18	Glycinin	PLG, PL, IG, GV, LG, LGV, PLG, VL, LV, VI	ACE inhibitor, opioid, glucose uptake stimulation, DPP-IV inhibitor
NRNGSHLPSYLPYP	1614.78	0.35	0.11	Glycinin	PYP, YL, YP, PSY, HL, GS, NG, SY, PQ, GSH, LPYP, PYPQ, NG, NR, PS, RN, SH, TL	ACE inhibitor, antioxidant, antiinflammation, neuropeptide, α-glucosidase inhibitor, DPP-IV inhibitor
NGSHLPSYLPYPQ	1472.62	0.49	0.08	Glycinin	PYP, YL, YP, PSY, HL, GS, NG, SY, PQ, GSH, LPYP, PYPQ, NG, NR, PS, RN, SH, TL	ACE inhibitor, antioxidant, antiinflammation, neuropeptide, α-glucosidase inhibitor, DPP-IV inhibitor
YIKDVF	783.92	0.39	0.07	Glycinin	VF, KD, YI	ACE inhibitor, DPP-IV inhibitor
NSLTLPALRQFG	1316.52	0.47	0.34	Glycinin	FG, LR, LP, SL, PA, AL, LT, QF, TL	ACE inhibitor, DPP-IV inhibitor
IDTNSFQNQLDQMPR	1806.97	0.20	0.11	Glycinin	PR, SF, FQ, MP, DQ, FQ, NQ, QL, QN, TN	ACE inhibitor, DPP-IV inhibitor
SGFAPEFLEHAFVVDRQ IV	2161.44	0.48	0.10	Glycinin	FAP, AF, AP, GF, SG, EF, DR, IV, FA, VV, HA, FL, DR, EH, QI, VD, PE, EF	ACE inhibitor, hypolipidemic, glucose uptake stimulation, α-glucosidase inhibitor, DPP-IV inhibitor
QNQLDQMPR	1129.26	0.48	0.25	Glycinin	PR, MP, DQ, NQ, QL, QN	ACE inhibitor, DPP-IV inhibitor
SAQFGSLRKNAMF	1456.68	0.82	0.22	Glycinin	MF, FG, GS, LR, SLR, SL, NA, QF, RK	ACE inhibitor, DPP-IV inhibitor
QNQLDQMPRRF	1432.62	0.74	0.34	Glycinin	RF, PR, RR, MP, DQ, NQ, QL, QN	ACE inhibitor, DPP-IV inhibitor
SAQFGSLRKNAM	1309.51	0.61	0.26	Glycinin	FG, GS, LR, SLR, SL, NA, QF, RK	ACE inhibitor, DPP-IV inhibitor
LKLSAQFGSLRKNAM	1664.00	0.51	0.42	Glycinin	LKL, FG, GS, KL, LR, SLR, SL, LK, NA, QF, RK	ACE inhibitor, antioxidant, DPP-IV inhibitor
IDTNSFQNQLDQMPRRF	2110.33	0.37	0.15	Glycinin	PR, PR, SF, RR, FQ, MP, DQ, NQ, QL, QN, TN	ACE inhibitor, DPP-IV inhibitor
SGFAPEFLEHA	1204.30	0.53	0.07	Glycinin	FAP, AP, GF, SG, EF, FA, HA, FL, EH, PE	ACE inhibitor, hypolipidemic, α-glucosidase inhibitor, DPP-IV inhibitor
IDTNSFQNQL	1179.25	0.21	0.07	Glycinin	SF, FQ, NQ, QL, QN, TN	ACE inhibitor, DPP-IV inhibitor
YHFREGDLI	1149.27	0.24	0.08	Glycinin	FR, GD, EG, YH, LI, HF	ACE inhibitor, glucose uptake stimulation, DPP-IV inhibitor
IYHFREGDL	1149.27	0.32	0.08	Glycinin	IY, FR, GD, EG, YH, HF	ACE inhibitor, antioxidant, DPP-IV inhibitor
EEKGAIVTVKGGL	1300.52	0.26	0.24	Glycinin	VK, GA, GL, KG, GG, AI, EK, IV, EE, TV, VK, VT	ACE inhibitor, stimulating vasoactive substance release, glucose uptake stimulation, DPP-IV inhibitor

PFSFLVPPKESQR	1531.77	0.46	0.15	Glycinin	VPP, VP, PPK, SF, PP, KE, LV, FL, ES, KE, PF, PK	ACE inhibitor, antiinflammation, antithrombotic, glucose uptake stimulation, α-glucosidase inhibitor, DPP-IV inhibitor
VIQQTFNL	962.11	0.29	0.12	Glycinin	TF, FN, IQ, NL, QQ, QT, VI	ACE inhibitor, DPP-IV inhibitor
IYIQQGSGIFG	1182.34	0.45	0.05	Glycinin	IY, IF, GI, FG, GS, QG, SG, IFG, IQ, QQ, YI	ACE inhibitor, antioxidant, DPP-IV inhibitor
RRQQARQVKNNNPFSF	1990.21	0.35	0.44	Glycinin	VK, SF, RR, AR, NP, NN, PF, QA, QQ, QV	ACE inhibitor, DPP-IV inhibitor
FQNQLDQMPRR	1432.62	0.66	0.33	Glycinin	PR, RR, FQ, MP, DQ, NQ, QL, QN	ACE inhibitor, DPP-IV inhibitor
LKLSAQFGS	950.10	0.24	0.15	Glycinin	LKL, FG, GS, KL, LK, QF	ACE inhibitor, antioxidant, DPP-IV inhibitor

[a]From PeptideRanker. [b]From CPPpred. [c]From ExPASy ProtParam. [d]From BIOPEP.

Table S7. Potential bioactive peptides in IPP-potato protein.

Peptide sequence	MW	PeptideRanker score[a]	CPPpred score[b]	Parent protein	Potential bioactive peptides[d]	Biological function[d]
LIVPQN	682.82	0.15	0.14	Globulin/ Legumin	VP, PQ, IV, LI, QN	ACE inhibitor, glucose uptake stimulation, DPP-IV inhibitor
RQGQLIVVPQN	1251.45	0.13	0.30	Globulin	VP, GQ, QG, PQ, IV, LI, VV, QL, QN	ACE inhibitor, neuropeptide, glucose uptake stimulation, DPP-IV inhibitor

[a]From PeptideRanker. [b]From CPPpred. [c]From ExPASy ProtParam. [d]From BIOPEP.
